# Second life and recycling: Energy and environmental sustainability perspectives for high-performance lithium-ion batteries

**DOI:** 10.1126/sciadv.abi7633

**Published:** 2021-11-05

**Authors:** Yanqiu Tao, Christopher D. Rahn, Lynden A. Archer, Fengqi You

**Affiliations:** 1 Robert Frederick Smith School of Chemical and Biomolecular Engineering, Cornell University, Ithaca, NY 14853, USA.; 2 Department of Mechanical Engineering, The Pennsylvania State University, University Park, PA 16802, USA.; 3 Department of Materials Science and Engineering, Cornell University, Ithaca, NY 14853, USA.

## Abstract

Second life and recycling of retired automotive lithium-ion batteries (LIBs) have drawn growing attention, as large volumes of LIBs will retire in the coming decade. Here, we illustrate how battery chemistry, use, and recycling can influence the energy and environmental sustainability of LIBs. We find that LIBs with higher specific energy show better life cycle environmental performances, but their environmental benefits from second life application are less pronounced. Direct cathode recycling is found to be the most effective in reducing life cycle environmental impacts, while hydrometallurgical recycling provides limited sustainability benefits for high-performance LIBs. Battery design with less aluminum and alternative anode materials, such as silicon-based anode, could enable more sustainable LIB recycling. Compared to directly recycling LIBs after their electric vehicle use, carbon footprint and energy use of LIBs recycled after their second life can be reduced by 8 to 17% and 2 to 6%, respectively.

## Teaser

Sustainability of reusing Li-ion batteries is hindered by high nickel content and is susceptible to use and recycling choices.

## INTRODUCTION

Owing to the rapid growth of the electric vehicle (EV) market since 2010 and the increasing need for massive electrochemical energy storage, the demand for lithium-ion batteries (LIBs) is expected to double by 2025 and quadruple by 2030 (*1*). As a consequence, global demands of critical materials used in LIBs, such as lithium and cobalt, are expected to grow at similar rates, leading to increased supply risk (*1*, *2*). To be specific, the global demands for lithium and cobalt are expected to increase around 10-fold from 2018 to 2030, surpassing the current supply (*3*, *4*). Concerns about lithium depletion have been extensively addressed in previous studies by showing that lithium does not face major supply risk in the mid-term future (*5*, *6*), but cobalt supply could be of great risk. Cobalt is produced mainly as the by-product of nickel and copper. Specifically, cobalt produced from copper mining is mostly geographically concentrated in Congo, and most cobalt refining facilities are located in China (*7*, *8*). Because of this by-product dependence and spatial distribution information, cobalt supply could be disrupted by the government policies or sociopolitical instabilities of these regions. As the market share of nickel-rich cathodes increases, Class 1 nickel, which is required for LIB cathode production, may also face supply chain challenges in the near future due to limited processing capacity (*9*). In addition, the scale of retired LIBs is expected to proliferate in the coming decade (*10*). All of these aspects contribute to the growing concerns on the resource depletion and environmental impacts resulting from the coming boom in retired LIBs (*2*).

The most effective approach to improving the sustainability of LIBs is to avoid the usage of critical materials, according to the waste management hierarchy that ranks the waste management approaches from the most to the least environmentally favorable (*2*, *5*). Along this line, both research and market interests shift toward low-cobalt LIBs and no-cobalt alternatives because of the concerns on cobalt supply and potential supply chain disruption, as well as the resulting price volatility and uncertainty (*1*, *3*, *7*, *11*). Notably, although the substitution of cobalt with manganese and nickel can increase the energy density and reduce the cost of LIBs, it sacrifices the structural stability and electrical conductivity of cathodes (*11*). The waste management hierarchy ranks the reuse of LIBs, such as the reuse as energy storage systems (ESSs) after automotive use, as the second ideal way to improve the sustainability of LIBs. Such a “second life” approach for automotive LIBs may improve both emission reduction benefits and economic performance (*12*, *13*). Nevertheless, according to existing material flow analysis, the second use of LIBs delays the recirculation of valuable metals, whose supply chains can become more vulnerable to disruption given their existing supply risks, compared to the case of direct recycling after automotive use (*14*, *15*). Therefore, there are trade-offs among the environmental benefits, economic values, and resource optimization. Existing literature on cascaded use (first use and second use) of LIBs focused on their technical and economic feasibility, as well as economic impacts on the global EV market (*14*, *16*, *17*). Previous life cycle assessment (LCA) studies on second life applications of LIBs mainly focused on only one type of battery chemistry [lithium iron phosphate (LFP), lithium manganese oxide (LMO), or LMO/lithium nickel manganese cobalt oxide (NMC)] (*12*, *18*–*23*). While multiple battery chemistries were considered by few studies (*12*, *24*, *25*), their environmental implications have not been explicitly investigated. Another less desirable strategy for retired LIB management integrates recycling, energy recovery, and disposal. Currently, both the pyrometallurgical and hydrometallurgical processes have been implemented at the laboratory, pilot, or commercial scale to recycle materials from waste LIBs (*26*). Because of the lack of available data, most existing LCA studies excluded or simplified the end-of-life (EOL) phase from the scope of their study (*27*–*31*). In particular, the environmental benefits of recycling could be overestimated because of missing critical steps and essential materials in existing LCA studies. Underestimation is also possible if the complete recovery of all cathode active materials, metals, and energy, or the enhancement of the recovery rates based on promising experimental data, is not considered. There is only limited systematic investigation on the trade-offs between the second life application and recycling of different types of automotive LIBs from the energy and environmental sustainability perspectives (*32*).

**Fig. 1.**
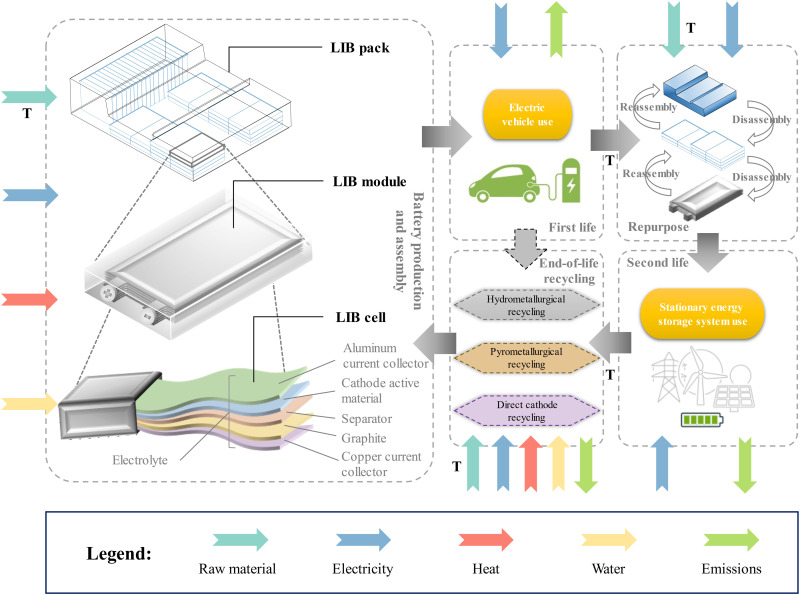
System boundary of LIB life cycle with second life and three EOL alternatives, including hydrometallurgical, pyrometallurgical, and direct cathode recycling. Transportation is abbreviated as T.

To fill the aforementioned knowledge gap, we aim to investigate the environmental benefits of second life and recycling approaches of automotive LIBs with different battery chemistries and to identify the environmental hotspots throughout their complete life cycles, emphasizing the maximum material and energy recovery. Specifically, a comprehensive list of environmental indicators (*33*), including carbon footprint and cumulative energy demand (CED), is examined for seven representative and promising automotive LIBs. Decisions to be made focus on the battery chemistry, use scenario, and EOL scenario. The currently commercial LIBs include LFP, three types of NMC (NMC333, NMC532, and NMC622), LMO/NMC532, and lithium nickel cobalt aluminum oxide (NCA); the prospective LIBs include the high-nickel and low-cobalt NMC811. For a fair comparison, a 52–kilowatt-hour (kWh) pack energy capacity is set for all types of LIBs (*34*). Moreover, to investigate the environmental benefits of second life adoption, two LIB use scenarios are proposed, as depicted in Fig. 1. The first one recycles the LIBs directly after automotive use, and the other one considers the second life application using LIBs retired from automotive use before LIB recycling. The global electricity demand is expected to grow at 2.1% per year until 2040 (*35*), and the requirement for power system flexibility becomes more stringent. Therefore, stationary ESS, as the fastest growing technology for enhancing power system flexibility, is considered as the second life application for retired automotive LIBs in this study.

We follow the existing approach to set the functional unit as the delivery of 1-kWh electricity over the life cycle of LIBs (*20*, *24*, *25*). Notably, the unit CED based on this functional unit is essentially the inverse of the energy return on investment, an important metric to measure the net energy profitability (*36*–*38*). Three popular EOL scenarios are assessed and compared, including hydrometallurgical, pyrometallurgical, and direct cathode recycling. These EOL scenarios are designed and optimized to achieve maximum material and energy recovery based on state-of-the-art experimental data (*39*–*42*). The pyrometallurgical recycling of LFP is disregarded because of the lack of valuable metals that are easily recyclable, such as nickel and cobalt. The temporal and spatial variations of the power grid are considered in the sensitivity analyses for the whole life cycle of LIBs. Specifically, the life cycle carbon footprint and CED for LIBs produced in each year from 2020 to 2050 are calculated according to the projected energy sources of electricity production for both the United States and China. Besides, environmental hotspots are identified to gain insights into the potential scale-up of laboratory-scale recycling technologies based on the state-of-the-art experimental results and the industrial-scale energy use and material consumption data (*43*). Key results and insights into benchwork, industry, and policy-makers are summarized in the following section.

### Use phase and EOL scenarios

**Fig. 2.**
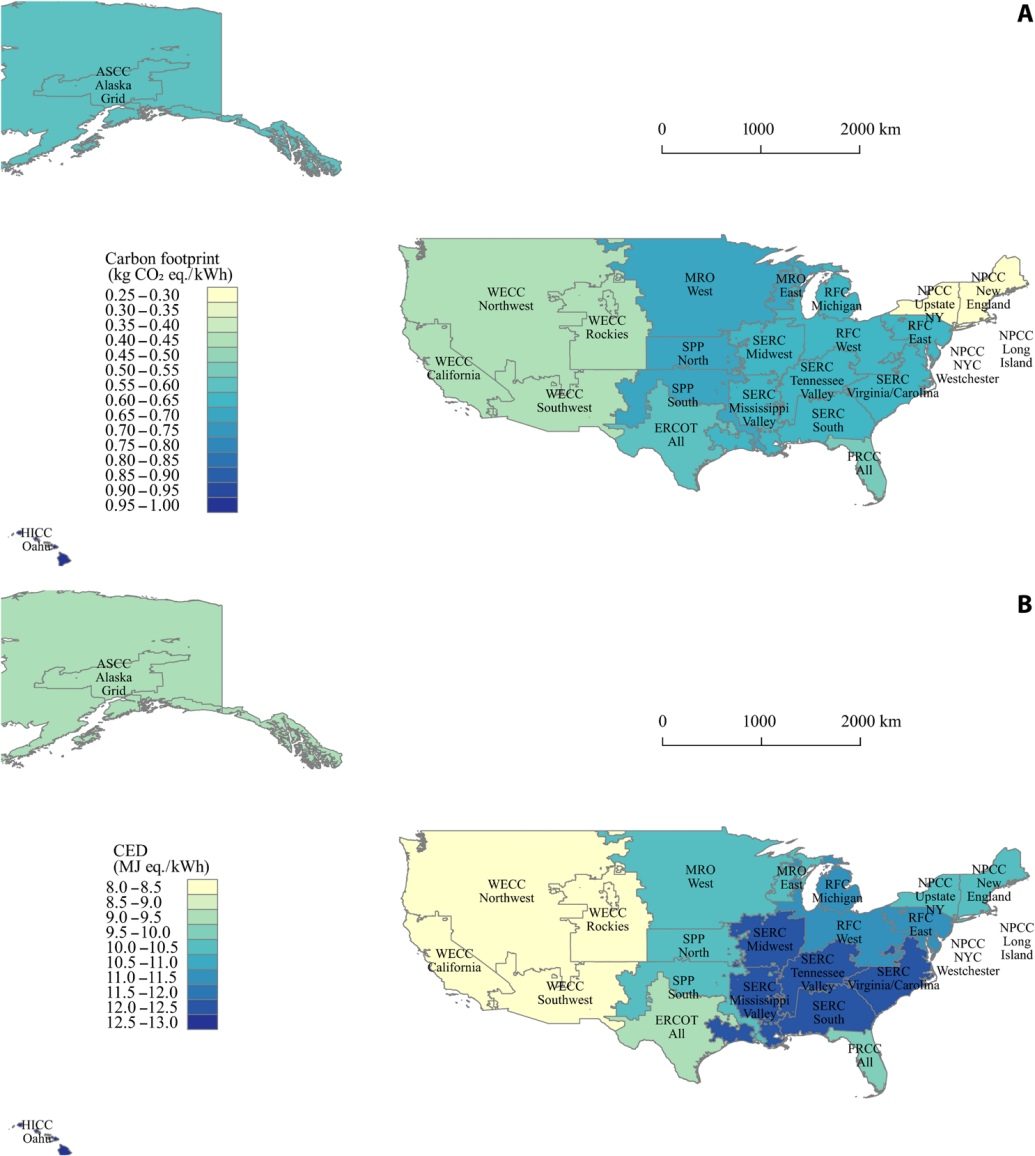
Carbon footprint and CED of power grid across U.S. independent system operators. (**A**) Carbon footprint. (**B**) CED. WECC, Western Electricity Coordinating Council; MRO, Midwest Reliability Organization; SERC, Southeastern Electric Reliability Council; ERCOT, Electric Reliability Organization of Texas; FRCC, Florida Reliability Coordinating Council; RFC, Reliability First Corporation; ASCC, Alaska Systems Coordinating Council; HICC, Hawaiian Islands Coordinating Council; SPP, Southwest Power Pool.

The size of the retired automotive LIB stockpile was expected to increase exponentially by 2025 (*44*), so it is crucial to introduce sustainable solutions, such as LIB reuse and recycling, to address the waste management challenges. In this study, two use scenarios are assessed: The first one is an 8-year EV use scenario, and the other one is the cascaded use scenario with a 10-year second life in stationary ESS after the 8-year EV use. To avoid confusion, the use phase for the EV use scenario refers to the LIBs used only in EV, and the use phase for the cascaded use scenario refers to LIB’s first life in EV and second life in the stationary ESS. LIB cells may fail during EV use because of extreme cycling or temperature conditions. In this study, no failure rate of LIB cells during EV use is considered, following the estimation of an existing work (*19*). Given the gap in long-term empirical data of battery degradation and lifetime distribution coefficients, the static lifetime of LIBs in EV and ESS is determined following previous literature (*45*, *46*). LIBs have a lifetime of 8 years in EV according to current calendar life warranty periods provided by most original equipment manufacturers (OEMs). We consider a lifetime of reused LIBs in ESS as 10 years following the most common assumption (*20*, *45*), but the lifetime of reused LIBs in ESS is highly uncertain. To address the uncertainty, we conduct a sensitivity analysis on the lifetime of LIBs with a range of 5 to 12 years for EV use and a range of 2 to 20 years for ESS use (*18*, *22*, *24*, *30*, *45*). Notably, the sensitivity analysis on the lifetime also addresses the uncertainty in the electricity consumption during EV use and ESS use. The results are presented in Discussion. All LIBs reach 80% of initial energy storage capacity at the end of their first life and 65% at the end of their second life. Fifty-five kilometers of EV use on a daily basis is considered following the 160934-kilometer warranty provided by most OEMs. Electricity delivery during EV use is determined by the OEMs’ tread life warranty, energy consumption per kilometer, roundtrip efficiency, and the electricity mix (*12*, *21*, *34*). For the stationary ESS use, electricity delivery during the ESS use is determined by the average daily electricity delivery, roundtrip efficiency, and the power grid (*12*, *21*). Daily discharge of 150 kWh on average is considered for a repurposed 450-kWh LIB pack, according to a previous study (*21*). Roundtrip efficiency of LIBs is considered to be 95% during EV use and 91% during stationary ESS use (*12*). New York State (NYS) is considered as the baseline location for the electricity generation throughout the life cycle of LIBs, because the Northeast Power Coordinating Council (NPCC) is the least carbon-intensive power grid in the United States, as shown in Fig. 2. Geospatial variation in the power grid may lead to large differences in analysis results of life cycle carbon footprint and CED of LIBs but would not result in diverse conclusions in the sustainability of second life and various recycling methods for different LIBs. More details of the use phase parameters can be found in table S1.

EOL of LIBs involves dismantling, material production, energy generation, incineration, combustion, waste sludge treatment, and energy and material recovery. The investigated EOL methods differ in the way that they recover energy and materials. To be specific, hydrometallurgical recycling recovers metals using aqueous chemistry and typically involves leaching, solvent extraction, and precipitation; direct cathode recycling directly recovers the cathode active materials through electrolyte extraction; pyrometallurgical recycling, as the most mature recycling method for LIBs, recovers metals in the form of alloy by a three-stage smelting process. Subsequent treatments, including a series of leaching, precipitation, and washing processes, are needed to obtain raw materials for producing the ready-to-use battery-grade cathode active materials. Given the fact that current recycling processes are not efficient enough for high-value metal recovery (*44*), all three recycling methods are optimized to recover as much cathode active materials as possible using the best-available laboratory-scale recycling procedures and experimental data, as depicted in figs. S4 to S6. The life cycle inventories (LCIs) of the three EOL scenarios are detailed in tables S10 to S18. The environmental impacts associated with energy and material recovery are considered as avoided burdens and are reported as reductions in emissions and CED, and the system boundary is expanded to a “cradle-to-cradle” counterpart.

### Key results and insights into benchwork, industry, and policy-makers

1) The maximized material and energy recovery can hardly offset the carbon footprint and CED from the intensive use of energy and chemicals during recycling processes, whereas it can largely eliminate by up to 68% of life cycle environmental impacts from other impact categories.

2) LIBs with higher specific energy density show better environmental performances, but their environmental benefits from second life application are less pronounced.

3) Compared to directly recycling LIBs after their EV use, life cycle carbon footprint and CED of LIBs recycled after their second life can be reduced by 8 to 17% and 2 to 6%, respectively, varying across battery chemistries and recycling methods.

4) Recycling methods and use scenarios are more impactful on the energy and environmental sustainability of LIBs, compared to the battery technologies.

5) The effects of battery chemistry and recycling methods on the life cycle carbon footprint and CED are negligible compared to the penetration of renewables in the power grid, with a reduction in carbon footprint in China (28.5%) twice as large as that in the United States (20%), although the absolute life cycle carbon footprint of LIBs in China is also twice of that in the United States.

6) Direct cathode recycling is the most environmentally favorable technology of LIB recycling, in concordance with previous findings (*31*, *47*).

7) Because the *N*-methyl-2-pyrrolidone (NMP) production and recovery are highly detrimental to the environment, greener aqueous binders should be further researched and developed for additional environmental benefits of both producing and recycling LIBs.

8) Carbon-intensive graphite and carbon black should be separated and recycled from the spent LIBs instead of being combusted to alleviate climate change.

9) Energy-intensive processes such as relithiation should be coupled with other exothermic processes to reduce energy demand.

10) Industrial recycling processes should be optimized to avoid the excessive use of environmentally expensive chemicals.

11) Battery design with less aluminum and alternative anode materials, such as silicon-based anode, could enable more sustainable pyrometallurgical recycling of LIBs.

12) Waste LIB sorting would become critical in improving the environmental sustainability of LIB recycling.

The importance of LIB design for recycling has been highlighted by previous literature (*2*, *48*). Standardized battery design with a simple disassembly mechanism, such as cell-to-pack technology, can help tackle the challenges in automation and robotic disassembly and improve recycling efficiency. With automated disassembly, rather than shredding, LIB recycling can get rid of many complicated separation processes, which would result in lower yield and product purity (*48*). Moreover, substituting polyvinylidene fluoride (PVDF) binder with aqueous binders not only provides environmental benefits but also can simplify the material recovery and improve the economic feasibility of LIB recycling (*2*, *48*). With optimized LIB design for automated disassembly and recycling, materials with higher purity and yield could be recovered with the aid of less energy and chemical inputs, hence producing further economic and environmental benefits (*49*).

## RESULTS

### Environmental impact reduction benefits of introducing second life

**Fig. 3.**
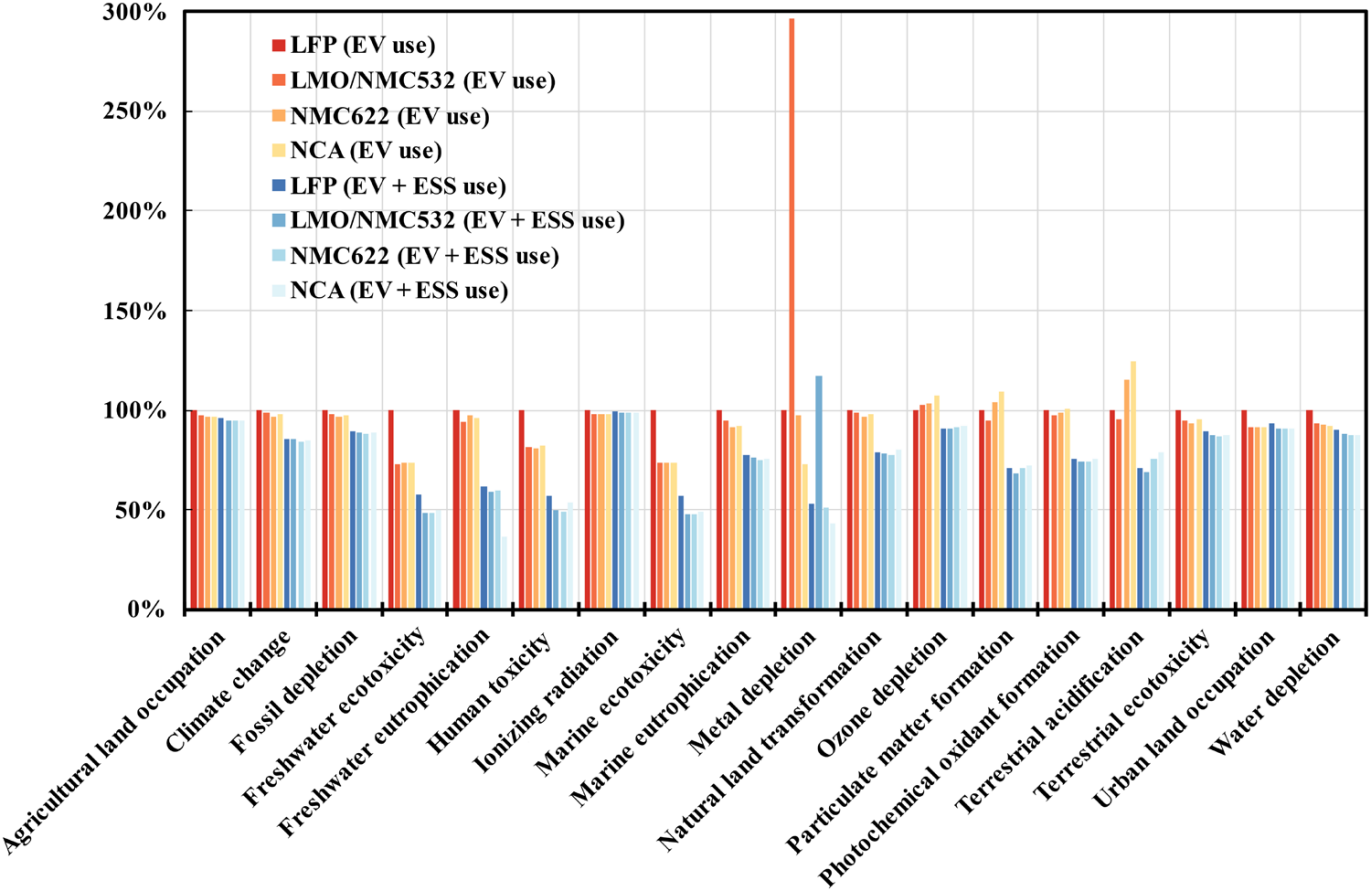
Comparison of environmental impacts between different use scenarios for LFP, LMO/NMC532, NMC622, and NCA LIBs. The environmental impacts of different recycling methods are averaged. Red and blue colors indicate life cycle environmental impacts associated with EV use scenario and cascaded use scenario, respectively. Darker color indicates lower pack energy density. For all 18 impact categories, the LFP LIB is defined as the reference for normalization.

Figure 3 presents the normalized life cycle environmental impacts of two use scenarios, EV use scenario and cascaded use scenario, across four types of LIBs representing the widely used cathode chemistry technologies (LFP, LMO/NMC532, NMC622, and NCA). For all 18 impact categories, the LFP LIB is defined as the reference for normalization. Adding second life greatly reduces environmental impacts, while the reductions in different impact categories vary substantially. In particular, the freshwater ecotoxicity, freshwater eutrophication, human toxicity, marine ecotoxicity, metal depletion, particular matter formation, and terrestrial acidification of all four types of LIBs reduce on average by more than 30%. These large reductions can be attributed to the difference of around three times in life cycle electricity delivery across the two use scenarios, coupled with the relatively minor contribution of electricity use to these impact categories. From the perspective of life cycle electricity delivery, the use phase results in the same environmental impacts for both use scenarios. Thus, the environmental benefits of second life application are larger when electricity use accounts for a lower proportion of environmental impacts. For other impact categories, using second life achieves less reduction benefits because their environmental impacts are dominated by electricity production. Discussion on the environmental profile of the NPCC power grid can be found in the Supplementary Materials. In general, the environmental profile of electricity use is determined by the power grid, considering the mix of different energy sources, electricity losses, and construction of distribution, transmission, and transformation networks, suggesting that it is possible to substantially reduce the environmental impacts of other impact categories by upgrading the power grid (*50*, *51*). The environmental impacts of natural land transformation for all types of LIBs are negative, as there is more land transformation to mineral extraction sites for metal production (*33*).

The results also suggest that LIBs with higher energy density show better environmental performances in most impact categories, but they benefit less from the second life application. As the specific energy density increases, the LIB production tends to be more environmentally friendly because of less material and energy input. Moreover, less material input for LIB production reduces recycling efforts. The recovery of cathode active materials for LIBs with higher energy density also avoids more environmental burdens. Thus, there is less potential for mitigating the environmental impacts of LIBs with higher energy density. Exceptions exist. For example, NCA LIBs perform the worst in three impact categories, including ozone depletion, particulate matter formation, and terrestrial acidification, because of the highest nickel content. The environmental impacts of high-nickel LIBs can be further deteriorated if nickel is produced from the Norilsk Nickel plant in Russia because of the uncontrolled SO_2_ emissions (*52*). In addition, the LMO recovery discards the leached ionic manganese from the spent cathode active materials and uses Mn_2_O_3_ as the manganese source of LMO, which deteriorates LMO/NMC532 LIB’s performance in metal depletion.

### Environmental impacts of battery recycling methods

**Fig. 4.**
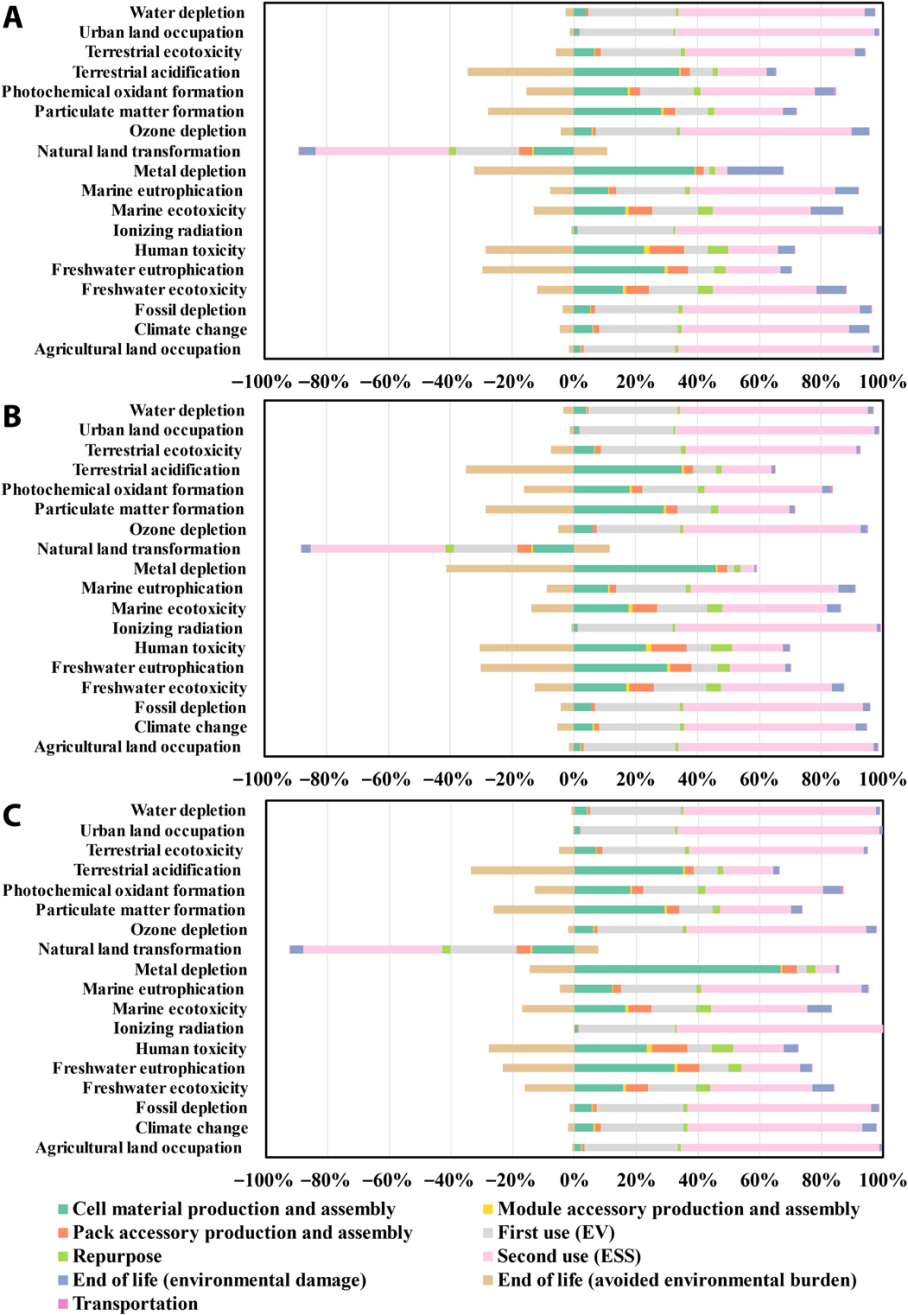
Comparison of full-spectrum environmental profiles for LMO/NMC532 LIBs across different recycling methods. Full-spectrum environmental profiles for LMO/NMC532 LIBs subjected to second life and recycled by (**A**) hydrometallurgical recycling, (**B**) direct cathode recycling, and (**C**) pyrometallurgical recycling on a percentage scale. Different colors in the stacked bars indicate different life cycle stages of LMO/NMC532 LIBs.

Figure 4 and figs. S13 to S18 depict the environmental profiles of LIBs for all impact categories on a percentage basis. The environmental impacts of each category are divided into different life cycle stages, with the use phase disaggregated into EV use and stationary ESS use to better understand their independent environmental impacts. Similarly, the EOL phase is disaggregated into two portions: One includes steps associated with environmental damages, and the other is responsible for the environmental burdens avoided from material and energy recovery.

All recycling methods are beneficial in most impact categories, namely, avoids more environmental burdens from material and energy recovery than causing environmental impacts from the intensive consumption of energy and chemicals. An exception to all types of LIBs is the net environmental burdens in ozone depletion of hydrometallurgical and pyrometallurgical recycling, which can be mostly explained by the direct and indirect methane emission from reagent production. Because of the recovery of less environmentally expensive cathode active materials and usages of reagents, such as citric acid and Mn_2_O_3_ for LMO recovery and H_3_PO_4_ for LFP recovery, hydrometallurgical recycling of LMO/NMC532 and LFP results in net environmental burdens in several other impact categories. More discussion is provided in the “Environmental hotspots” section. In terms of natural land transformation, the positive environmental impacts of material and energy recovery are attributable to the land transformation from the mineral extraction site induced by metal recovery. Among the three recycling methods, direct cathode recycling is the most environmentally friendly regardless of battery chemistry for three reasons. First, material recovery of direct cathode recycling and hydrometallurgical recycling avoids comparable environmental impacts, but energy and materials used in hydrometallurgical recycling result in much higher environmental impacts than those used in direct cathode recycling. Second, compared to other recycling methods, pyrometallurgical recycling of LMO/NMC532, NMC, and NCA LIBs recovers much less valuable metal (96% of Ni and 62% of Co), generates a large quantity of nonrecyclable aluminum and lithium in slag from the smelting process, and uses large doses of environmentally expensive reductants. Moreover, other volatile LIB components, including the separator, electrolyte, binder, graphite, and carbon black, are combusted and evaporated in the furnace. Third, the absence of cobalt and nickel in LMO suggests no recovery of valuable metals, and much fewer reductants are needed accordingly. However, the use of citric acid (leachate) and Mn_2_O_3_ (manganese source) causes notably higher environmental impacts in several impact categories than the LMO avoided from material recovery in hydrometallurgical recycling of LMO/NMC532.

### Potential of mitigating climate change and energy demand

**Fig. 5.**
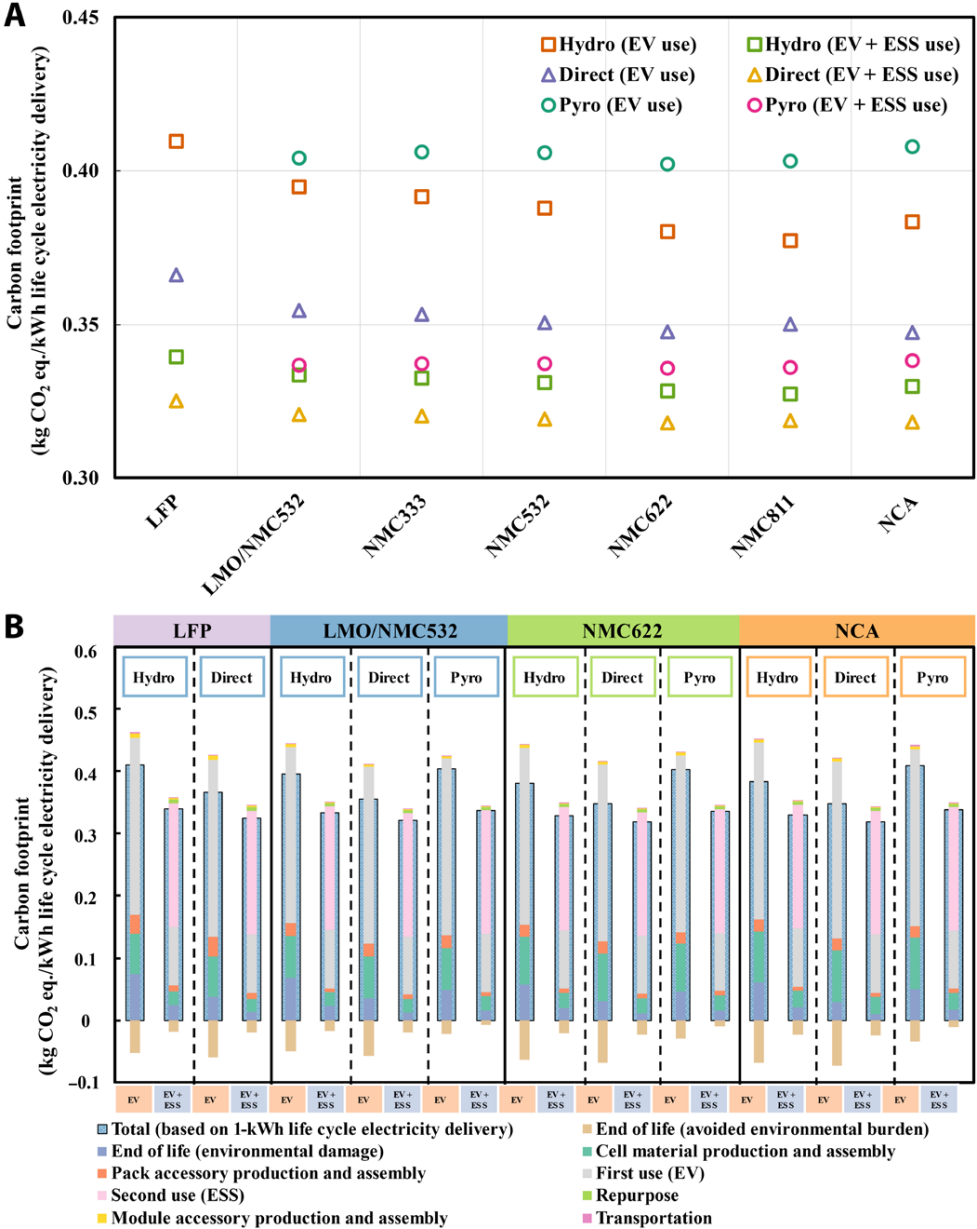
Overview of carbon footprint for the LFP, LMO/NMC532, NMC333, NMC532, NMC622, NMC811, and NCA LIBs with different EOL scenarios. (**A**) Life cycle carbon footprint for the seven types of LIBs with different EOL scenarios. (**B**) Breakdowns of the carbon footprint for LFP, LMO/NMC532, NMC622, and NCA LIBs with different EOL scenarios. The stacked bar plot represents the breakdowns of carbon footprint per kilowatt-hour life cycle electricity delivered to the stage level. Different colors indicate different stages throughout LIB’s life cycle, as stated in the legend. The hydrometallurgical, direct cathode, and pyrometallurgical recycling are abbreviated as hydro, direct, and pyro.

Carbon footprint and CED are two important metrics to evaluate the climate change mitigation potential and energy performance of introducing second life and recycling into batteries’ life cycle. Adding second life reduces the carbon footprint by 8 to 17% and the CED by 2 to 6%, depending on the specific battery chemistry and recycling method. Keeping the recycling method and use scenario fixed, increased nickel content and decreased cobalt content of LIBs tend to shift their life cycle carbon footprint and CED downward (Fig. 5A and fig. S7A) because of less material and energy required for both production and recycling. However, as the nickel content continues to rise in LIBs, that is, NMC811 and NCA, the environmental impacts of cathode active materials increase. This counterintuitive result is presumably due to (i) more electricity consumption for calcination of materials rich in nickel; (ii) usage of more carbon- and energy-intensive lithium source of LiOH instead of Li_2_CO_3_; (iii) the increasing nickel content does not only replace cobalt content, which is relatively more carbon and energy intensive, but also replaces the relatively abundant and environmentally benign manganese content. The carbon footprint and CED of NMC811 with pyrometallurgical and direct cathode recycling are slightly higher than that of NMC622, mainly because of the employment of around twice the amount of PVDF used in other LIBs. NCA has the highest nickel content, but the usage of carbon- and energy-intensive HCl in hydrometallurgical and pyrometallurgical recycling of NCA, instead of H_2_SO_4_ for other types of LIBs, leads to increases in both carbon footprint and CED. This result suggests that the most environmentally friendly recycling option for the cathode active materials is not only to pursue the least cobalt content, and careful life cycle environmental evaluation in production and recycling processes is needed before any generous incentive or subsidies are given.

Recycling methods and use scenarios are more impactful on the carbon footprint and CED of LIBs, compared to the battery technologies. Among the three EOL scenarios, direct cathode recycling remains the least carbon and energy intensive for all LIBs, while the maximized material recovery of hydrometallurgical and pyrometallurgical recycling can hardly offset the carbon footprint and CED from the intensive use of energy and chemicals during the recycling processes. Pyrometallurgical recycling of LMO/NMC532 LIBs and hydrometallurgical recycling of LFP LIBs even result in a net positive carbon footprint and CED. Moreover, hydrometallurgical recycling of LMO/NMC532 LIBs and pyrometallurgical recycling of NMC622 and NCA LIBs all result in non-negligible carbon burdens, although they are energy saving. As cascaded use accounts for a larger portion of life cycle environmental impacts and needs more material and energy inputs for repurposing, the second life application of LIBs could hinder the environmental benefits of LIB recycling. This result illustrates the environmental trade-off between second life application and recycling of LIBs.

Advanced LIB technologies with high specific energy density do not necessarily demonstrate better potentials for mitigating climate change and energy demand, especially when the material and energy inputs for the LIB production and recycling are highly carbon and energy intensive. The development of green recycling processes with higher material recovery rates, lower energy requirement, and utilization of less environmentally expensive materials is critical to improving the potential of mitigating environmental impacts. Moreover, their potentials for mitigating climate change and energy demand are confined by the penetration of renewable electricity. Therefore, it is essential to increase the share of renewable energy in the local power grid. To promote the decarbonization of the LIB supply chain and renewable energy generation of LIB manufacturing, the European Union (EU) policy-makers aim to regulate the LIBs traded on the EU market (*53*). In the next sections, we will discuss how and to what extent we can further reduce the carbon footprint and CED of all types of LIBs with different recycling methods.

### Environmental hotspots

**Fig. 6.**
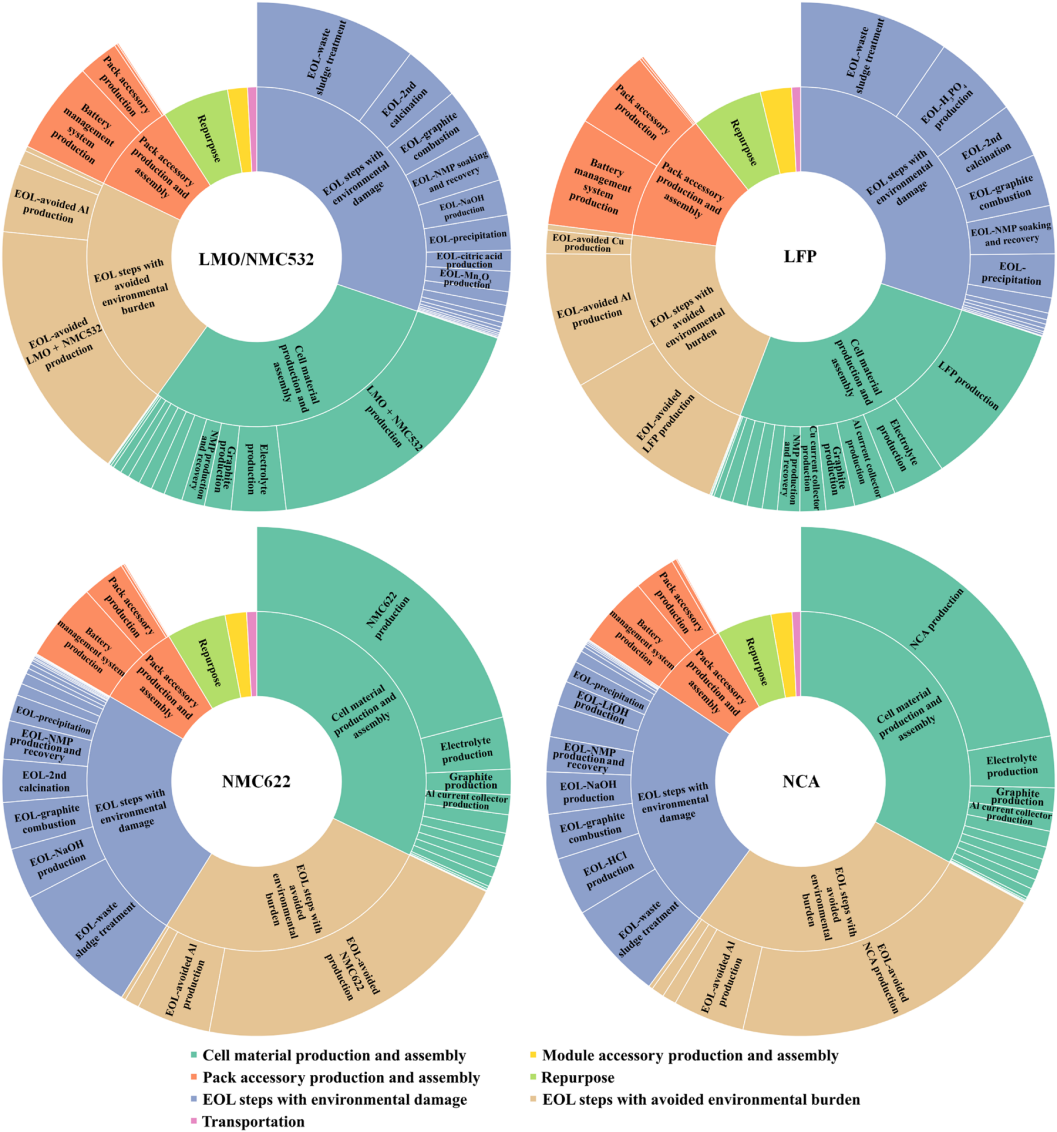
Carbon footprint hotspots of LFP, LMO/NMC532, NMC622, and NCA LIBs with hydrometallurgical recycling. The surrounding sunburst charts represent the hierarchical results of the carbon footprint from the life cycle stages to the process level. The inner circle represents the upper-level stages, while the outer circle represents the lower-level processes of each stage. The colors of stages and their corresponding processes are consistent, and the value of each process and stage is proportional to the angle of concentric circles. Moreover, starting from the top, the shares of the carbon footprint for stages become smaller in a clockwise order; within each stage, the shares of the carbon footprint for lower-level processes become smaller in the same manner.

Impacts of different life cycle stages of reused automotive LIBs on carbon footprint and CED have been explicated in the previous section. To further decipher environmental hotspots embedded in each stage, sunburst charts representing hierarchical results of carbon footprint and CED are depicted in Fig. 6 and figs. S8 to S12. Sunburst charts reveal the contributions of lower-level processes within upper-level life cycle stages. Absolute values of the negative carbon footprint and CED that resulted from material and energy recovery are used for comparison among different life cycle stages. In addition, the use phase is identified as an overriding life cycle stage in terms of carbon footprint and CED, so it is not discussed in this section.

For the cell material production and assembly stage, cathode active material is the predominant factor of the carbon footprint for LMO/NMC532 (60%), NMC622 (65%), and NCA (67%) LIBs. On the contrary, LFP production accounts for only 41% of the carbon footprint associated with this stage. This contrast can be attributed to the high carbon footprint associated with NiSO_4_ and CoSO_4_ production, high heat and energy demand, and heavy use of the precipitant (NaOH) during the NMC and NCA production.

The battery management system (BMS) is the main contributor to the carbon footprint of LIB pack accessory production. Production of BMS and other pack accessories, including compression plates and straps, module interconnects, and a trilayer jacket, together is responsible for nearly all the carbon footprint associated with this stage. The dominant role of BMS and other pack accessories can be attributed to the production of printed wiring boards and aluminum-made outer and inner layers of the battery jackets, respectively.

The roles of EOL steps in carbon footprint depend on the battery chemistry and the specificities of EOL scenarios. First, material and energy recovery during hydrometallurgical and direct cathode recycling reduces slightly less carbon footprint than the amount added by the cell material production and assembly stage. On the contrary, pyrometallurgical recycling is deficient in material recovery because it retrieves nickel as Ni(OH)_2_ and recovers cobalt in the form of ionic solutions. Moreover, it only recovers aluminum during the dismantling of the LIB pack but does not recover lithium and aluminum from the subsequent smelting step. This may present a challenge as the EU proposed to mandate the recycling of valuable metals (*53*). Specifically, in descending order, recovery of cathode active material, aluminum, and LiPF_6_ constitutes the vast majority of carbon footprint reduced by direct cathode recycling; recovery of cathode active material and aluminum dominates the carbon footprint reduction for hydrometallurgical recycling; Ni(OH)_2_ recovery of pyrometallurgical recycling is the major source to reduce carbon footprint. Second, other than direct cathode recycling, hydrometallurgical recycling of NMC622 and NCA generates less greenhouse gas emissions than avoided from their material and energy recovery. Among these greenhouse gas–emitting steps, waste sludge treatment is the most influential one for both hydrometallurgical and pyrometallurgical recycling. This is because they both adopt hydrometallurgical steps, such as leaching, solvent extraction, and precipitation, that eventually discharge a large amount of waste solvent sludge. Instead, liquid CO_2_, which consumes plenty of electricity, is much more energy intensive than other energy-consuming EOL steps for direct cathode recycling. Moreover, graphite combustion remains one of the most influential steps for all three recycling methods, which suggests a need to suppress graphite combustion to further mitigate carbon footprint. Soaking and recovery of the binder solvent NMP are also a major carbon footprint and CED contributor for both hydrometallurgical and direct cathode recycling. This can be mainly attributed to the need for steam and wastewater treatment for NMP recovery. To further reduce the carbon footprint and CED of battery recycling, especially for hydrometallurgical and direct cathode recycling, research and development on replacing or avoiding the step of binder solvent recovery are highly recommended. Last, according to the battery chemistry, different leaching agents and precipitants with a variety of reaction conditions are selected to recover the cathode active materials for hydrometallurgical and pyrometallurgical recycling, resulting in multiple levels of carbon burden. It is worth mentioning that these EOL steps’ contributions to the carbon footprint and CED are relatively comparable, and none of them are dominant.

**Fig. 7.**
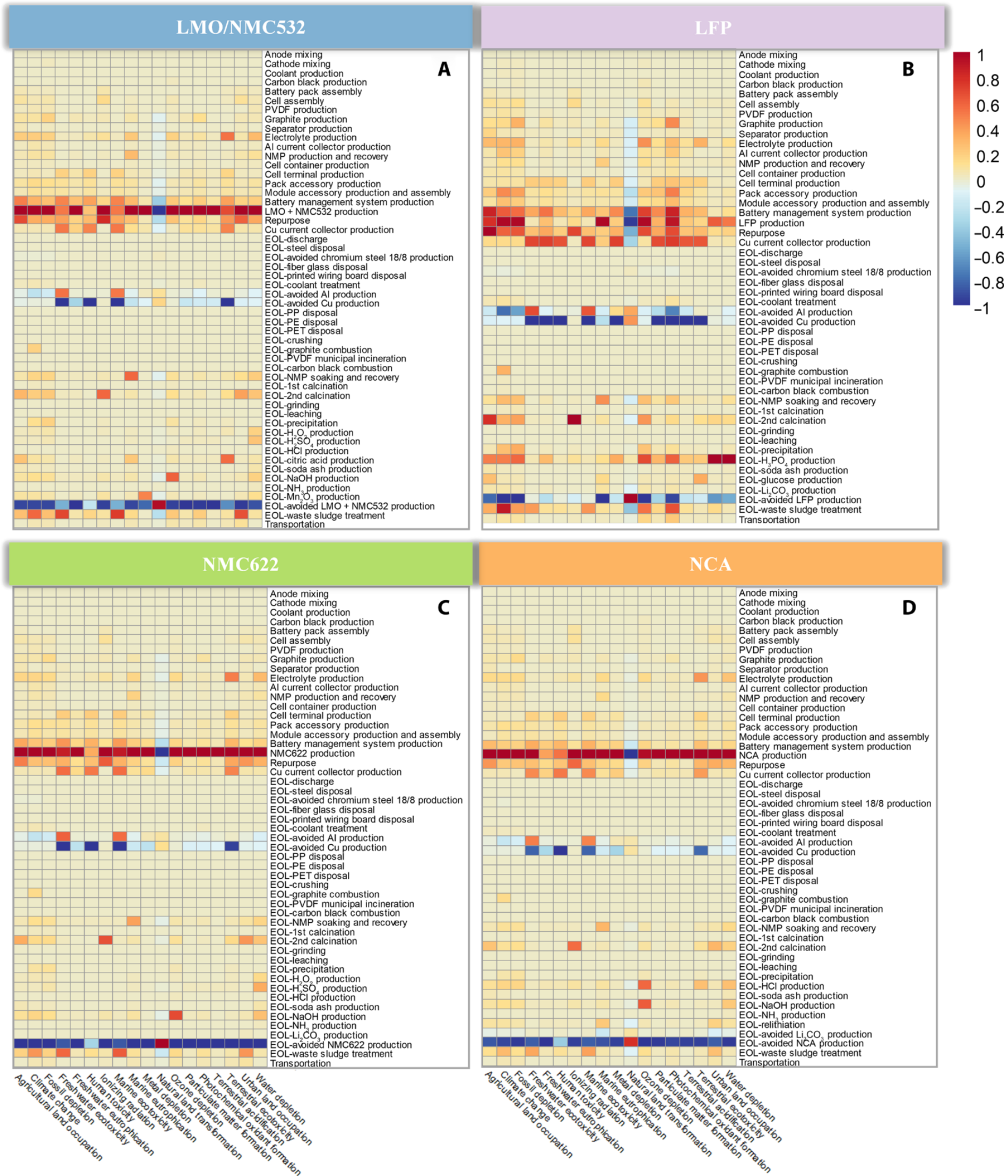
Full-spectrum environmental hotspots for LFP, LMO/NMC532, NMC622, and NCA LIBs with hydrometallurgical recycling. (**A**) Full-spectrum environmental hotspots for LMO/NMC532 with hydrometallurgical recycling. (**B**) Full-spectrum environmental hotspots for LFP with hydrometallurgical recycling. (**C**) Full-spectrum environmental hotspots for NMC622 with hydrometallurgical recycling. (**D**) Full-spectrum environmental hotspots for NCA with hydrometallurgical recycling. Use phases are excluded from the system boundary. Colors represent values corresponding to the environmental impacts of each process under each impact category. The values are normalized using the min-max normalization method and vary according to the colors presented on the color bar. In particular, the red color represents positive values and suggests damage to the environment; the blue color represents negative values and indicates an avoidance of environmental burden. Moreover, the darker color along each column implies more environmental impacts on the corresponding impact category. PET, polyethylene terephthalate; PP, polypropylene; PE, polyethylene.

To explicitly identify the environmental hotspots across the full spectrum of impact categories, we aggregate the normalized LCI data to process level and visualize them using a heatmap, as shown in Fig. 7 and figs. S19 and S20. For all types of LIBs, recovery of cathode active material, Ni(OH)_2_, and metals are major contributors to alleviate environmental impacts. Notably, LFP, LMO/NMC532, and Ni(OH)_2_ recovery is not as environmentally valuable as NMC and NCA recovery in all environmental impact categories, suggesting the necessity of waste LIB sorting by battery chemistry before the recycling. In addition, disassembling LIBs into constituents of cathode, anode, and casing for the hydrometallurgical and direct cathode recycling approaches prevents the subsequent anode-cathode separation and diminishes cathode active material loss and contamination. On the other hand, manual disassembly is labor intensive and could potentially cause hazards through thermal runaways and toxic chemicals (*2*). On the contrary, the direct comminution of LIB cells is labor saving but mixes waste streams in the black mass, complicates the downstream processing of metal recovery, lowers the product purity, and results in more environmental impacts (*47*, *48*). LiPF_6_ recovery of direct cathode recycling is another moderate contributor to reduce environmental impacts from the categories of agricultural land occupation, climate change, fossil depletion, ionizing radiation, marine eutrophication, ozone depletion, terrestrial ecotoxicity, and water depletion. The result also suggests that cathode active material production is the major contributor to environmental impacts.

Environmental hotspots are specific to recycling methods. For both hydrometallurgical and pyrometallurgical recycling of LMO/NMC532, NMC, and NCA, the production of leaching agent and precipitant is more impactful in contributing to ozone depletion, terrestrial ecotoxicity, and water depletion than to other impact categories. However, for hydrometallurgical recycling of LFP, the production of H_3_PO_4_ as a leaching agent accounts for more than 10% of life cycle metal and water depletion. Because of the large electricity consumption, liquid CO_2_ production of direct cathode recycling for different second life LIBs contributes substantially to climate change (23 to 35%), fossil depletion (38 to 50%), ionizing radiation (89 to 92%), ozone depletion (39 to 55%), and urban land occupation (59 to 81%) associated with the EOL steps causing environmental damages.

Notably, environmental hotspots are not always extensively distributed across the impact categories. For example, copper recovery is not comparable with aluminum recovery in terms of climate change and fossil depletion. However, copper recovery can largely reduce environmental impacts through the categories of freshwater ecotoxicity, freshwater eutrophication, human toxicity, marine ecotoxicity, metal depletion, and terrestrial ecotoxicity; in addition to fossil depletion, aluminum recovery leads to much environmental burden on freshwater and marine ecotoxicity; NMP soaking and recovery contribute to a large portion of the marine eutrophication, although it only makes a minor contribution to most of the impact categories.

### Temporal and geographical variability

Previous results show that environmental impacts associated with the use phase are overwhelming and unavoidable. Because electricity consumption is the only process in the use phase that causes damages to the environment, an effective approach to minimizing the environmental impacts of the use phase is to make the electricity production less carbon intensive. To test the sensitivity of second life LIB’s environmental performance to the temporal and spatial variability of electricity production, we establish a prospective LCA model by integrating the projected electricity production for 2020–2050 from the U.S. Energy Information Administration (EIA) on the basis of the reference static LCA (*54*, *55*). In particular, the year-specific environmental impacts of electricity production are based on the projected proportion of energy sources, as shown in fig. S33. Moreover, the United States and China are selected for this sensitivity analysis because these two largest LIB manufacturers and consumers contribute to a combined total of 88% of global LIB production capacity and 62% of global EV stock (*56*–*58*). Notably, only the environmental profile of electricity production is altered for the manufacturing of cathode active materials and LIBs, use phase, and recycling processes according to the geographical and temporal variation in the power grid. The supply chains of raw materials for the cathode active materials, other LIB components, and material inputs for the recycling processes are consistent with those in the baseline case.

**Fig. 8.**
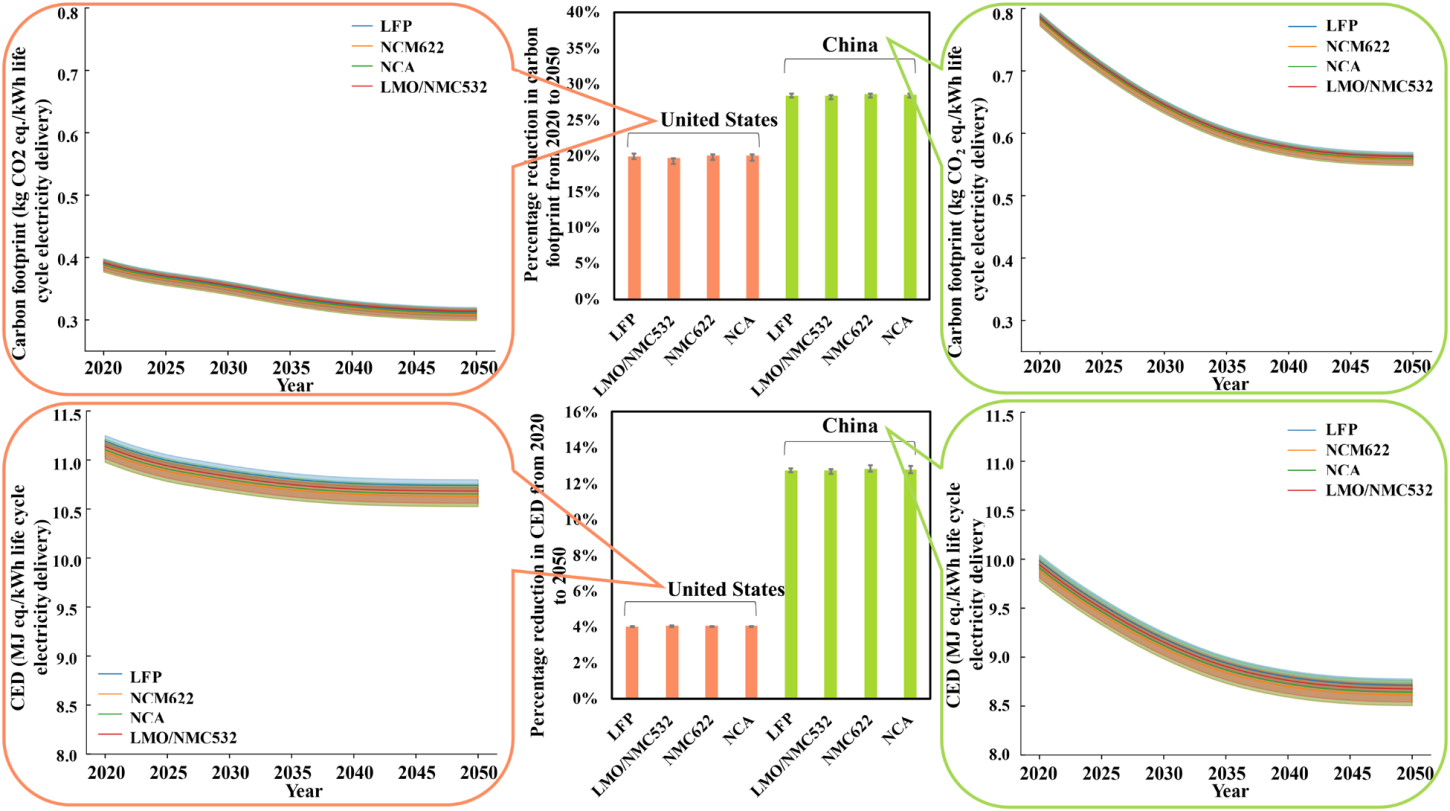
Sensitivity analysis of temporal and spatial variations in electricity generation from 2020 to 2050 in the United States and China. The horizontal axis represents the year when the LIBs are produced. The vertical axis represents the life cycle carbon footprint and CED for LIBs produced each year. Notably, the carbon footprint and CED of electricity production depend on the starting year of each life cycle stage.

Figure 8 demonstrates the life cycle carbon footprint and CED for the LFP, LMO/NMC532, NMC622, and NCA LIBs produced each year. Compared to the electricity generation in 2020, greenhouse gas emission in 2050 is reduced by 20% for LIBs produced, consumed, and recycled in the United States and by 28.5% for those in China. This difference in carbon footprint mitigation potential is attributed to the critical difference in energy sources of electricity generation in 2020 for the United States and China. To be specific, coal, which is relatively carbon intensive, accounts for 62% of electricity generation in China in 2020, and its share decreases to 30% in 2050 by projection (*54*). On the contrary, coal-fired power generation only accounts for 24% of the total electricity production in the United States in 2020 and will decrease to 12% in 2050 (*55*). The share of renewable sources in China will increase substantially from 36% in 2020 to 63% in 2050. Similarly, the share of renewable sources in the United States increases from 40 to 56% during 2020–2050. This suggests that for regions without a strong penetration of renewables in the power grid, energy system decarbonization has a great potential to substantially cut down LIB’s life cycle carbon footprint. Natural gas, which is a relatively clean energy source, will remain the leading energy source in the United States for the next three decades, accounting for 38 to 32% of the electricity production from 2020 to 2050, according to EIA’s projection (*55*). Although electricity production in China shows greater climate change mitigation potential in the next three decades, the life cycle carbon footprint of LIBs in China would remain higher than that in the United States from 2020 to 2050. This trend is consistent with a recent study, although they projected the carbon footprint for only the use phase in 2030 (*59*).

Similarly, the results show that LIBs produced, consumed, and recycled in China lead to more energy saving than those in the United States for the next three decades, although the CED for average electricity production in the United States is 12% higher than that in China for 2020. This is mainly attributed to the higher CED required for coal-fired electricity production in the U.S. CED of generating 1-kWh electricity, and it is directly related to the energy efficiency of the power plants. Existing studies and government data suggest that the average energy efficiency of China’s coal-fired power plants surpasses the average energy efficiency of coal-fired power plants in the United States (*60*, *61*). While the average energy efficiency of other energy sources, including natural gas, wind, geothermal, solar photovoltaics, and hydropower, in the United States is higher than those in China, the resulting decrease in CED cannot offset the increase in CED caused by the relatively lower energy efficiency of coal-fired electricity. Also, the projected reduction of coal-fired electricity in the United States is less than that in China for 2020–2050. As a consequence, the gap of CED for average electricity generation between the United States and China is further widening, varying from 12% in 2020 to 23% in 2050.

The shaded areas in Fig. 8 represent the variation of recycling methods, suggesting that the effect of both battery chemistry and recycling method on life cycle carbon footprint and CED is negligible relative to the impact of renewable penetration in the power grid. Although the best-available projection for 2020–2050 from the U.S. EIA is integrated into this study, we are aware that the current results may be conservative, and the potential of mitigating climate change and energy demand for 2050 can be even greater, given the recent ambitious climate policies of the United States and China.

## DISCUSSION

Toward the urgent need of prolonging the driving range of EV, the nickel-rich low-cobalt cathode is at the forefront of achieving higher energy density and reducing the supply risk of cobalt (*3*, *62*). Previous studies showed that the increase of nickel content would trigger severe capacity fading and thermal safety hazards (*63*). Nevertheless, a recent study has demonstrated promising performances of single-crystal NMC532: mild capacity fading and outstanding lifetimes of more than 1.6 million kilometers (*3*, *64*, *65*). There is still room for improvement of the production cost, specific capacity, and rate capability for these new-generation, single-crystal high-nickel LIBs. Moreover, because of the requirement of high temperature, above 930°C for 12 hours, for production (*65*), more inferior environmental performance is expected as compared to the results in this study (Figs. 3 and 5). Our results show that the strategy of substituting cobalt with nickel tends to improve the environmental performance of LIBs, but this benefit is very susceptible to the choice of recycling method and use scenario. Moreover, the environmental impacts of global nickel production are hindered by the uncontrolled SO_2_ emissions from the Norilsk Nickel plant in Russia (*52*). In addition, substituting cobalt with nickel may pose a 60-fold increase in nickel demand by 2030 and up to 190 folds by 2050, compared with the 2017 values, and further investigation on the nickel supply is required (*45*). Owing to its low specific energy density, the production of LFP LIBs is found to be the most detrimental to the environment, despite its cobalt- and nickel-free characteristics. The recent revolution in cell-to-pack technology could narrow the gap between the battery pack energy density of LFP and its NMC/NCA counterparts and subsequently lower the demand in material and energy for packing by optimizing the design and assembly of LIB cells (*66*, *67*). Because of the reduced material and energy input, the environmental impacts of LIB manufacturing can be mitigated. Nevertheless, the cell-to-pack technology can also facilitate the automation of disassembly and consequently improve the recycling efficiency (*48*).

LIBs retain a rather high energy storage capacity after their first life in EV, so the resources used for battery production are not fully exploited if they are sent to EOL directly after EV use. However, by reusing automotive LIBs in less demanding second life applications, the recovery and recirculation of valuable metals can be delayed for many years, leading to increasing supply risks (*14*, *15*). With second life, less reduction of carbon footprint and CED can be achieved by the high-nickel NMC and NCA compared to the widely used LFP. Uncertainty in the lifetime of EV use and ESS use does not affect this conclusion despite their strong impact on the life cycle carbon footprint and CED of LIBs (figs. S24 to S28). It should be noted that this conclusion is premised on the basis of the same pack energy capacity for a fair comparison across battery chemistries. Future LIBs may have higher pack energy capacities (up to 100 kWh per EV) (*68*). With a higher pack energy capacity, LIBs show worse environmental performances due to more resources consumed for LIB production and recycling. The environmental benefits of high-capacity LIBs from the second life application are more prominent than those of the lower-capacity ones. However, with the same pack energy capacity, the findings of this study remain the same for high-capacity LIBs. Moreover, the second life application of LIBs hinders the environmental benefits of recycling, as it contributes to a larger portion of life cycle environmental impacts and requires additional resources for repurposing. Sensitivity analysis results on use parameters suggest a great potential to further reduce carbon footprint and CED of reused LIBs. Even with a rather conservative transition toward more than 50% penetration of renewable energy sources into the power grid, the carbon footprint of second life LIBs can be reduced by 20% in the United States and by 28.5% in China. As the power grid transitions to all-renewable energy sources, substantial environmental impacts can be further reduced for LIBs. For the sake of climate change and energy demand, direct cathode recycling should be the fate of waste LIBs, although it has less ideal recovery rates of materials, as shown in figs. S21 to S23.

### Implications on LIB recycling

Direct cathode recycling is a strong candidate for enhancing the sustainability of LIBs and promoting the circular economy, as illustrated by existing studies (*31*, *47*, *69*). Through modeling of maximized material recovery, our results show that direct cathode recycling is even more environmentally favorable compared to the existing literature (fig. S2) (*31*). Considering the increasing demand for LIBs and a potential shortage of cobalt in 30 years (*1*, *8*), deployment of direct cathode recycling with a 95% recovery rate of cathode active materials could largely mitigate the risk of metal depletion and relieve the pressure of metal supply on the global market. For this reason, it is crucial to gain more insights into its scalability and potentials for improvement. First, the electrolyte extraction efficiency for liquid CO_2_ with additives of propylene carbonate and acetonitrile should be improved. Moreover, the current electrolyte extraction technology can be replaced by less energy-intensive and more environmentally friendly alternatives. The combination of acutely toxic, irritating binder solvent NMP and mutagenic binder PVDF could be replaced by greener alternatives, which resonates with previous studies. For example, the combinations of aqueous binders and corresponding binder solvents (i.e., water) have the properties of being fluorine-free, ease of disposal, and availability from renewable resources (*70*, *71*). Moreover, water is used as the binder solvent that does not need to be recovered, so the environmental burdens caused by solvent recovery can be avoided. Although the field of aqueous binders is rather unexplored, previous studies show promising results on the enhanced electrochemical performance of LIBs (*72*–*74*). Furthermore, the combustion of graphite and carbon black takes a great share of carbon burdens caused by the EOL phase. Recycling graphite from waste LIBs at the laboratory scale has been assessed and could be further explored and scaled up (*75*, *76*). Last, the energy-intensive hydrothermal and annealing process can be coupled with other exothermic processes to reduce the energy demand. However, because of the rapid evolutions in cathode chemistry of LIBs and the delayed recycling processes by decades, the scale-up of direct cathode recycling could be impeded by its limited flexibility to generate the state-of-the-art cathode active materials (*2*, *47*). The prerequisite of waste LIB sorting also renders direct cathode recycling less attractive to the recyclers (*2*, *44*).

For pyrometallurgical recycling, maximized material and energy recovery of LIBs cannot offset the carbon footprint caused by the intensive use of energy and chemicals. Thus, battery design with less aluminum use and alternative anode materials, such as silicon-based anode, could enable more sustainable pyrometallurgical recycling of LIBs. In addition, further research is required to study the substitution of the current environmentally detrimental leaching agents and precipitants with green alternatives that would not decrease the high recovery rates. Hydrometallurgical recycling induces environmental burdens for battery chemistry with less valuable metal utilization (e.g., LFP and LMO/NMC). Thus, waste LIBs should be carefully sorted. Waste LIB sorting by battery chemistry can also benefit the environmental sustainability of pyrometallurgical and hydrometallurgical recycling by avoiding excessive use of environmentally expensive chemicals (*77*).

## MATERIALS AND METHODS

### Goal and scope definition

The “cradle-to-grave” LCA study in this work investigates the carbon footprint, CED, and full-spectrum environmental impacts associated with the production, consumption, and EOL of seven automotive LIBs, namely, LFP, LMO/NMC532, NMC333, NMC532, NMC622, NMC811, and NCA, after second life in stationary ESS. The designed specific energy densities in the BatPac model are 177, 229, 234, 243, 255, 265, and 262 Wh/kg for LFP, LMO/NMC532, NMC333, NMC532, NMC622, NMC811, and NCA LIB packs, respectively (*34*). The life cycle stages within the scope of this study are listed as below:

1) Cell material production and assembly

2) Module accessory production and assembly

3) Pack accessory production and assembly

4) Use phase (EV use or EV + ESS use)

5) Repurpose (accompanied with the cascaded use scenario)

6) EOL phase (can be divided into EOL steps with environmental damage and EOL steps with environmental burden)

7) Transportation

The functional unit of 1-kWh life cycle electricity delivery is used to quantify the environmental impact based on the life cycle energy provision of LIBs. Working parameters of LIBs for both EV and stationary ESS use are provided in table S1. Other than transportation, the whole life cycle of the LIBs, including production, EV use, repurpose, stationary ESS use, and recycling, is assumed to be located in the NYS in 2018 under the baseline case, without considering the temporal and spatial variations in the power grid. For this reason, the environmental impacts associated with electricity consumption remain constant in the baseline case, and the environmental profile of electricity production is determined by the energy sources of the NPCC in 2018. The carbon footprint and CED of electricity generation in other areas of the United States can be found in Fig. 1. Besides, under the cascaded use scenario, LIBs undergo the repurpose processes, which dismantle the LIB packs to the module level, change a part of the components (such as antifreeze agents, LIB pack casing, and module interconnects), test the cells, and reassemble 450-kWh LIB packs for stationary ESS use.

Because one of the primary objectives of this study is to investigate the environmental benefits of the second life for different LIBs, two use scenarios are considered, as shown in Fig. 2. The first one is the EV use scenario, of which the system boundary involves stages of cell material production and assembly, module accessory production and assembly, pack accessory production and assembly, EV use, and EOL recycling. The other one is the cascaded use scenario, which has a system boundary, including stationary ESS use (second use) and repurpose, in addition to all the stages of the aforementioned EV use scenario. Transportations are also included in both system boundaries and are estimated from existing studies, as shown in table S23 (*21*, *31*). Another goal of this study is to evaluate the environmental impacts of various recycling methods, so three EOL scenarios, including hydrometallurgical, pyrometallurgical, and direct cathode recycling, are systematically analyzed and compared. It is worth mentioning that only hydrometallurgical and direct cathode recycling are adopted for LFP because of the lack of valuable metals that are recyclable using pyrometallurgical recycling. Moreover, hydrometallurgical and direct cathode recycling are closed-loop recycling processes, which recover the cathode active materials from the spent LIBs. On the contrary, pyrometallurgical recycling is an open-loop recycling process as it recovers nickel as Ni(OH)_2_ and cobalt as a salt, both for reentering in the battery supply chain (*78*). The environmental impact of the open-loop pyrometallurgical process is equivalent to its closed-loop counterpart because replacing the cobalt source of the cathode active material production with the recycled cobalt salt would result in only the avoided environmental burden from the recycled cobalt salt. More details about use scenarios and EOL scenarios are provided in the next sections and the Supplementary Materials.

For the computation of the full-spectrum environmental impacts, 18 ReCiPe midpoint indicators from the hierarchist perspective are adopted to examine the severity of the environmental impact categories (*33*). These indicators account for agricultural land occupation, climate change, fossil depletion, freshwater ecotoxicity, freshwater eutrophication, human toxicity, ionizing radiation, marine ecotoxicity, marine eutrophication, metal depletion, natural land transformation, ozone depletion, particulate matter formation, photochemical oxidant formation, terrestrial acidification, terrestrial ecotoxicity, urban land occupation, and water depletion. This ReCiPe model is frequently used in LCA studies on LIBs (*20*, *25*, *29*, *79*).

### LCI analysis

During the LCI analysis phase of LCA, energy and material flows are quantified and compiled across all life cycle stages of the LIBs. Within the production stages, LCIs of an EV battery pack are presented in tables S2 to S6. LCIs of a stationary ESS LIB pack after automotive use are provided in table S7. The EOL stage involves three EOL scenarios that correspond to LCIs summarized in tables S10 and S11, S12 and S13, and S15 and S16. Because the LCIs of cathode active material production are unavailable in the existing LCI database, their manufacturing routes are extracted from the literature, as shown in fig. S3, and LCIs are established and compiled by modeling the detailed manufacturing processes, as shown in tables S9 and S14.

### Life cycle impact assessment method

In this study, carbon footprint, CED, and ReCiPe impact categories are selected to demonstrate and compare the life cycle greenhouse gas emissions, energy consumption, and full-spectrum environmental impacts, respectively. In the life cycle impact assessment stage of LCA, LCIs are computed on the basis of the functional unit through characterization factors to quantify their environmental impacts for each impact category. We collect most of the characterization factors from Ecoinvent, and lists of these characterization factors can be found in table S25 (*80*). However, characterization factors for some processes, such as LFP, NMC, and NCA production, are inaccessible from the Ecoinvent database. CoSO_4_ and Ni(OH)_2_, which are raw materials of cathode active materials (for cobalt-containing LIBs and nickel-metal hydride batteries), do not have readily available LCI data either. Then, we need to construct the LCI from the upstream processes [i.e., Ni(OH)_2_, CoSO_4,_ NMC, and NCA production estimated from upstream energy and material inputs detailed in the Supplementary Materials].

### Sensitivity analysis

Sensitivity analysis is performed to evaluate the key assumptions of electricity generation and EOL scenarios. According to the results shown in Fig. 5 and fig. S7, the use phase is the leading factor of the life cycle carbon footprint and CED. Moreover, the use phase is the main contributor for most of the environmental impact categories, as shown in Fig. 4. We also conclude that the full-spectrum environmental impact profiles of LIBs are largely affected by the energy sources of electricity generation and the characteristics of those energy sources. To assess the temporal and spatial variation in electricity production, we integrate the projected power grid of the United States and China from 2020 to 2050 into our model. The United States and China are chosen as they are two countries with the largest production capacities of automotive LIBs and the largest EV markets in the Eastern and Western Hemisphere (*54*–*56*). The environmental impacts associated with 1-kWh electricity generated in each year are computed as a weighted sum of the unit environmental impacts for electricity production from various energy sources. The weights are the shares of different energy sources. The unit environmental impact for electricity production from each energy source in each location is obtained from the Ecoinvent database (*80*). The electricity generation by energy source from 2020 to 2050 in the United States and China is presented on a percentage basis in fig. S33. The manufacturing of cathode active materials and LIBs, use phase, and recycling processes are considered to be in the United States and China, while the supply chains of raw materials for the cathode active materials, other LIB components, and material inputs for the recycling processes are consistent with those in the baseline case. LIBs produced in each year from 2020 to 2050 are used by EVs for 8 years, according to the current calendar life warranty periods provided by OEMs. After retiring from EV use, LIBs are repurposed to start their second life. After the 10-year second life, whose lifetime is considered on the basis of the most common assumption from existing literature, LIBs are disposed of and recycled. For example, a LIB pack can be produced in 2020, repurposed in 2028, and recycled in 2037, with the first life in 2020–2027 and the second life in 2028–2037. It is worth mentioning that all the other assumptions related to battery parameters, LIB production, repurpose, and LIB recycling, remain unchanged. Sensitivity analyses on other battery parameters, including LIB lifetime, roundtrip efficiency, and energy consumption rate in EV, are conducted separately, and the results are presented in figs. S24 to S28, S30, and S31. In addition, we do not consider the technology development of batteries and power grid across time, such as the increase in charge-discharge efficiency and transmission efficiency, the transition toward novel materials, and the improvement of power generation technologies and energy consumption during EV use (*30*). On the basis of the functional unit of 1-kWh life cycle electricity delivery, life cycle carbon footprint and CED are calculated for LFP, LMO/NMC532, NMC622, and NCA LIBs over the period of 2020–2050. Note that the energy sources of electricity generation vary in the 18-year life cycle of LIBs, and we assume that the power grid will remain invariant after 2050 because of the lack of projected power grid data for both countries after 2050.

The investigated parameters regarding EOL scenarios are considered according to the carbon and energy hotspots identified in Fig. 6 and figs. S8 to S12. In particular, the following parameters are included for all three EOL scenarios: the recovery rate of chromium steel 18/8, aluminum, copper, and graphite. The recovery rate of cathode active materials and NMP is evaluated for hydrometallurgical and direct cathode recycling. Specifically, the recovery rate of LiPF_6_ is assessed for direct cathode recycling; the recovery rate of goethite, cobalt, and Ni(OH)_2_ is investigated for pyrometallurgical recycling; and the recovery rate of Mn_2_O_3_ and citric acid is evaluated exclusively for hydrometallurgical recycling of LMO/NMC532. Ranges of parameters are presented in tables S19 to S22, and impacts of these parameters are shown in figs. S21 to S23. The results of sensitivity analyses are discussed in the Supplementary Materials.

### Min-max normalization

To intuitively present the life cycle environmental impacts across each impact category, we adopt the min-max normalization method to process data. For a set of data points *X*_1_, *X*_2_, …, *X_n_* (i.e., environmental impacts of all processes for each category), this normalization method linearly maps each data point to the range of 0 to 1 according to Eq. 1, where *X*′, *X*_a_, *X*_max_, and X_min_ represent each data point after normalization, each data point before normalization, the minimum of the dataset, and the maximum of the dataset, separately

$$X\prime =\frac{X_{a}-X_{\text{min}}}{X_{\text{max}}-X_{\text{min}}}$$ (1)

Nevertheless, the environmental impacts associated with the avoided environmental burden in the EOL phase are negatively signed. Hence, the magnitude of both negative and positive values should be shown on the same basis, while the orientation of environmental favorability (i.e., the negative signs) for each process is preserved. However, Eq. 1 is not able to preserve negative signs. To address this issue, we first take the absolute values of the negative numbers, then apply min-max normalization according to Eq. 1, and lastly change the sign of those that were negative to negative.

## Supplementary Materials

### This PDF file includes:

Supplementary Text Figs. S1 to S33 Tables S1 to S25 References
