# Supplementary material for: Second life and recycling: Energy and environmental sustainability perspectives for high-performance lithium-ion batteries
Source: Sci Adv. 2021 Nov 5;7(45):eabi7633. doi: 10.1126/sciadv.abi7633 (PMC8570603; doi:10.1126/sciadv.abi7633)
Supplement: Supplementary file 1 — Supplementary Text Figs. S1 to S33 Tables S1 to S25 References [file sciadv.abi7633_sm.pdf]

Science Advances
AAAS

# Supplementary Materials for

## Second life and recycling: Energy and environmental sustainability perspectives for high-performance lithium-ion batteries

Yanqiu Tao, Christopher D. Rahn, Lynden A. Archer, Fengqi You*

*Corresponding author. Email: fengqi.you@cornell.edu

Published 5 November 2021, *Sci. Adv.* **7**, eabi7633 (2021)
DOI: 10.1126/sciadv.abi7633

**This PDF file includes:**

Supplementary Text
Figs. S1 to S33
Tables S1 to S25
References

## Supplementary Text

### Comparison with results from existing literature

Fig. S1. summarizes carbon footprint results from existing life cycle assessment (LCA) studies on reused automotive lithium-ion batteries (LIBs) and compares them with the carbon footprint results from this work. As most existing work on LIB's second life did not assess energy performance or investigate a comprehensive list of impact categories, the results of cumulative energy demand (CED) and full-spectrum environmental impacts are not comparable. It is noticeable that there are only a few LCA studies on second life adoption of retired automotive LIBs. Neither of these works included direct cathode recycling as an alternative recycling method nor considered LIBs other than lithium manganese oxide (LMO) and lithium iron phosphate (LFP). Some of them excluded LIB production, electric vehicle (EV) use, and end-of-life (EOL) recycling from the system boundary (*12, 24*). Besides, several studies simplified the EOL recycling by omitting critical steps or using the unit process for LIB treatment from the Ecoinvent database (*18, 20, 21*). Hence it could result in large uncertainty in their results.

The life cycle carbon footprint calculated in this study is slightly higher than those from the previous literature (columns in Fig. S1). These variabilities can be mainly attributed to differences in the choice of system specifications related to the use phase, such as choice of electricity mix (Ahmadi. et al. (*20*) and Faria et al. (*18*)), modeling of life cycle energy consumption, or only the energy loss due to battery weight and charge-discharge efficiency (Richa et al. (*21*)), and amount of electricity delivery during second life (all three studies). Ahmadi. et al. (*20*) and Faria et al. (*18*) investigated the life cycle carbon footprint of reusing LFP LIBs. For example, due to the high penetration of renewable energy sources in the chosen Ontario grid mix (*20*) and French grid mix (*18*), the carbon footprint associated with the use phase is only around 0.14 and 0.06 kg $CO_2$ eq. per life cycle electricity delivery, respectively, which is much lower than that in the baseline scenario of this study (0.27 kg $CO_2$ per life cycle electricity delivery for the Northeast Power Coordinating Council, or NPCC). If the same assumption of the electricity grid is applied, they should produce a higher life cycle carbon footprint than this study.

Due to the inconsistencies in the assumptions related to the use phase and the substantial impacts of the use phase on the life cycle carbon footprint, we recalculate the life cycle carbon footprint by excluding the use phase from the system boundary (red circles in Fig. S1). Besides the difference in battery chemistries, the assumptions for both the LIB use in stationary energy storage systems (ESS) and the EOL recycling could contribute to the difference in the carbon footprint results. First, Richa et al. (*21*) assumed a 50% cell conversion rate for the repurposing of retired EV LIB pack, while a 100% cell conversion rate is considered in this study. Under the same assumption of 100% cell conversion rate, the carbon footprint allocated to the LIB production and recycling in Richa et al. (*21*) could be reduced by more than half. Second, the EOL recycling in Faria et al. (*18*) and Richa et al. (*21*) were highly simplified. For example, in Richa et al. (*21*), neither a metallurgical charge comprising iron and slag formers (including limestone, sand, slag, and coke) or leaching agents (such as HCl and $H_2SO_4$) was used in the pyrometallurgical recycling. With respect to the hydrometallurgical recycling in Richa et al. (*21*), materials for essential steps, such as N-Methyl-2-Pyrrolidone (NMP), material and energy input for NMP soaking and recovery (i.e. nitrogen, steam, and water), and precipitant for lithium carbonate ($Li_2CO_3$), are missing. This could lead to an underestimated carbon footprint

associated with EOL recycling. On the other hand, Richa et al. (*21*) considered only the avoided burden from steel, aluminum, and copper recycling and neglected the recovery of critical metals, which could on the contrary lead to overestimated carbon footprints. Faria et al. (*18*) and Ahmadi. et al. (*20*) estimated the carbon footprint of hydrometallurgical recycling based on data from the Ecoinvent database. Since Faria et al. (*18*) and Ahmadi. et al. (*20*) did not provide details about the EOL recycling of LIBs, we will not discuss its carbon footprint. Lastly, the assumed life cycle electricity delivery in this study is higher. If all other assumptions are the same, higher life cycle electricity delivery will cause a lower life cycle carbon footprint.

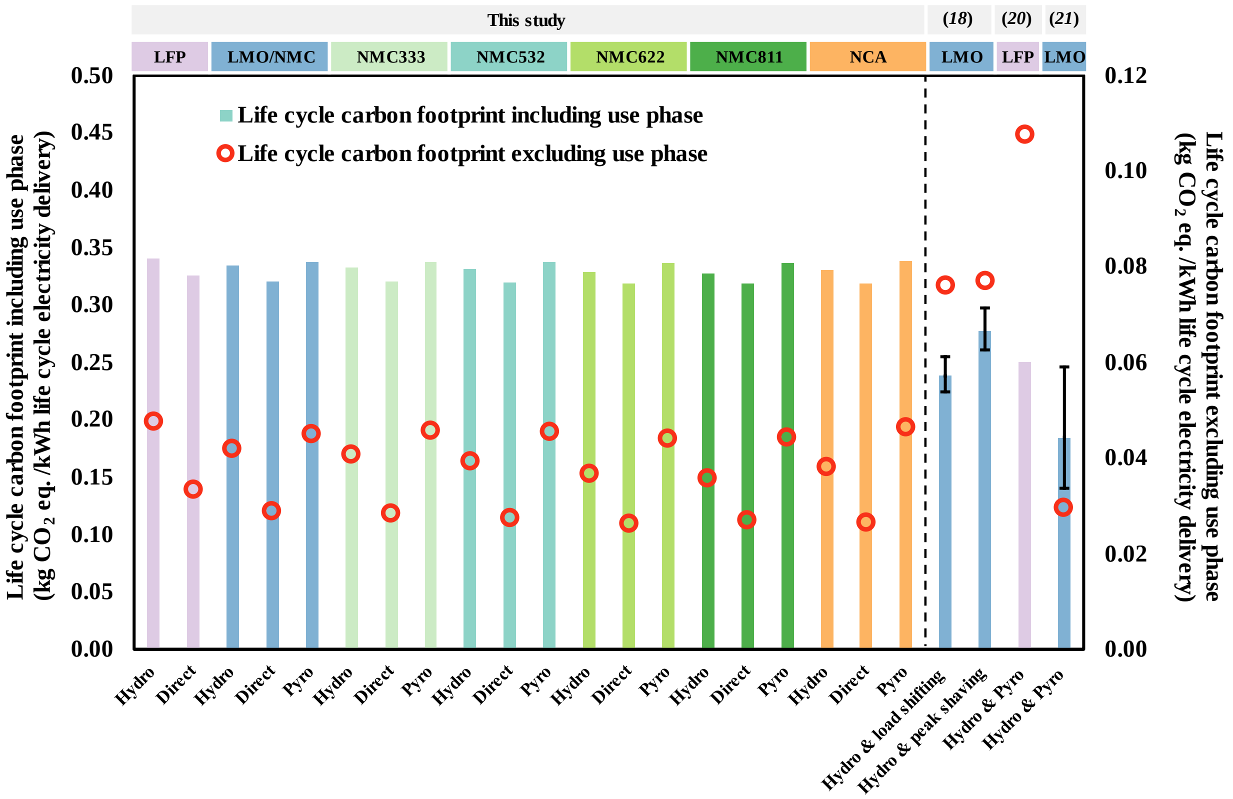


**Fig. S1. Comparison of LCA studies on LIBs second life adoption of retired automotive LIBs.**

The carbon footprint and CED resulted from battery recycling methods are summarized and compared in Fig. S2. There are two most common approaches used in EOL models: the avoided burden approach and the recycled content approach (*81*). The avoided burden approach assumes that the recycled material or energy can offset the same amount of primary material and energy production. The recycled content approach assumes that no credits are given for material or energy recycling and no burdens are given for secondary material usage. Accordingly, the environmental impacts resulted from the recycled content approach are nonnegative and the environmental impacts resulted from the avoided burden approach can be either negative or positive. For a fair comparison, only the results of the avoided burden approaches are compared. Among all studies with the avoided burden assumption, the process designs of recycling in Dunn et al. (*82*) are obtained from the GREET model which is also the basis of process flows in this analysis. We do not compare our study with Dunn et al. (*82*) because the battery chemistries are different. The carbon mitigation potential of hydrometallurgical and direct cathode recycling for NMC622 and lithium nickel cobalt aluminum oxide (NCA) LIBs in this study is better than that from Ciez et al. (*31*). This is because the "best-available" laboratory procedures to achieve the maximum material recovery rate were not fully considered by Ciez et al. (*31*) and other LCA studies on LIB recycling. On the contrary, the carbon footprint of pyrometallurgical recycling for NCA in this study is higher than that from Ciez et al. (*31*). A potential reason could be the omission of some essential steps of pyrometallurgical recycling in Ciez et al. (*31*) and other works. For example, the steps of reducing alloy generated from smelting into recyclable products such as iron, copper, and nickel hydroxide ($Ni(OH)_2$). In addition, the emitted flue gas from electrolyte combustion and blast furnace gas from coking and alloy formation is often neglected by existing LCA studies on battery recycling. Therefore, the environmental benefits of pyrometallurgical recycling are often overestimated.

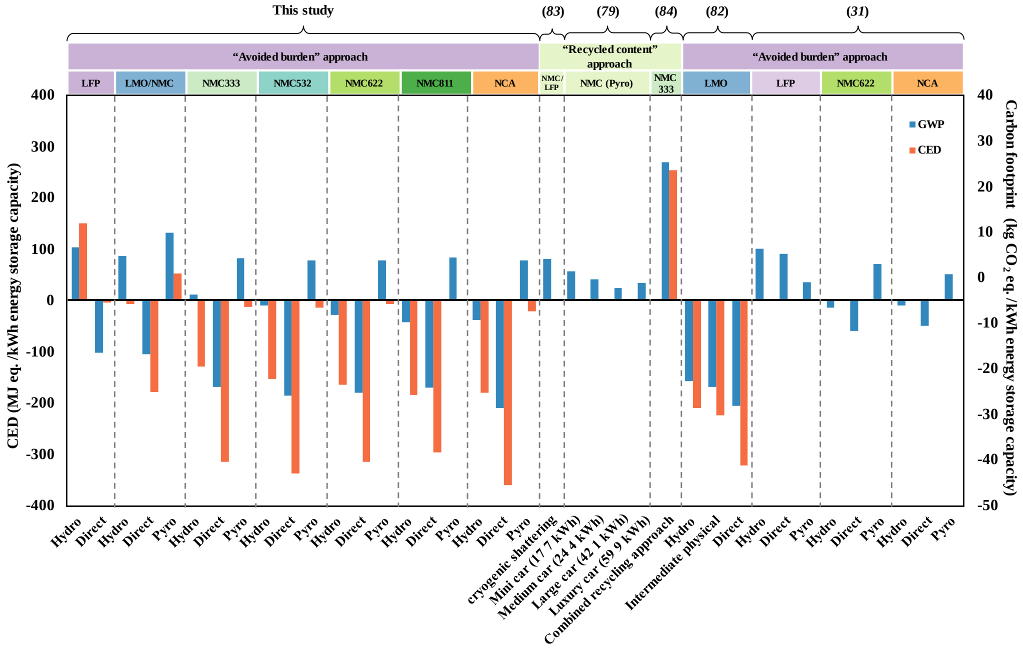


**Fig. S2. Comparison of LCA studies on carbon footprint and CED resulted from battery recycling.**

## Battery Parameters

Battery parameters, such as energy consumption rate in electric vehicles, specific energy density, and energy storage capacity, are extracted from the BatPac model (*34*). Other battery parameters, including residual battery capacity after EV use and after stationary ESS use, roundtrip charge/discharge efficiency for EV use and stationary ESS use, and daily delivery of electricity from single stationary ESS battery packs are extracted from the previous studies (*20*, *21*, *85*) and presented in Table S1. Electric vehicle use is set to be 55 km per day over an 8-year timeframe for both of the use scenarios, and the battery energy capacity decreases from 100% to 80%. Starting from an initial discharging efficiency of 80%, 150-kWh delivery on a daily basis for 10 years with an EOL discharging efficiency of 65% is considered for the ESS use (*86*). The lifetime of EV battery packs and stationery ESS battery packs are considered following the most common assumptions (*20*, *45*, *46*, *87*).

**Table S1. Battery parameters of LIBs.**

| Battery parameter | LFP | LMO/ NMC532 | NMC 333 | NMC 532 | NMC 622 | NMC 811 | NCA |
|---|---|---|---|---|---|---|---|
| Initial battery capacity | | | | 100% | | | |
| Residual battery capacity after EV use (*85*) | | | | 80% | | | |
| Residual battery capacity after ESS use (*21*) | | | | 65% | | | |
| Lifetime of EV battery packs (year) (*45*) | | | | 8 | | | |
| Lifetime of stationary ESS battery packs (year) (*45*) | | | | 10 | | | |
| Daily discharge of electricity of single stationary ESS battery pack (kWh) (*21*) | | | | 150 | | | |
| Daily traveled distance (km) (*21*) | | | | 55 | | | |
| EV energy consumption rate (kWh/km) (*34*) | | | | 0.155 | | | |
| Battery pack energy density (Wh/kg) (*34*) | 177 | 229 | 234 | 243 | 255 | 265 | 262 |
| Roundtrip efficiency during EV use (*12*) | | | | 95% | | | |
| Roundtrip efficiency during ESS use (*12*) | | | | 91% | | | |
| Electricity delivery during EV use | | | | 269259 | | | |
| Electricity delivery during ESS use | | | | 547500 | | | |
| Battery pack energy storage capacity (kWh) (*34*) | | | | 52 | | | |

## Life Cycle Inventory

The industry-level data for LIB battery production is not easily accessible to the public and is usually used with low transparency to protect confidentiality (*79*). Hence in this paper, the inventory of the battery pack is based on secondary data from the BatPac model, as provided in Tables S2–S6 (*34*). The inventory of a stationary ESS battery pack is presented in Table S7. It is worth mentioning that the environmental impacts associated with cathode active material of LMO are obtainable directly through the Ecoinvent database, where the avoided environmental impacts associated with $Ni(OH)_2$ recovery and cathode active material production for LFP, NMC, and NCA are inaccessible. Instead, the data on upstream materials and energy for producing $Ni(OH)_2$ and LFP are sourced from Majeau-Bettez et al. (*29*), and the cathode active materials of NMC and NCA are acquired from the GREET model (*88, 89*). The process models of cathode active materials are detailed in the next section.

**Table S2. Material input and output of single LIB cell (*34*).**

| Input | Component (g) | LFP | LMO/ NMC532 | NMC333 | NMC532 | NMC622 | NMC811 | NCA |
|---|---|---|---|---|---|---|---|---|
| Cathode | Cathode active material | 448.08 | 430.80 | 387.67 | 374.35 | 326.80 | 277.40 | 299.71 |
| | Conductor: Carbon black | 9.33 | 8.98 | 8.08 | 7.80 | 6.81 | 15.41 | 6.24 |
| | Binder: Polyvinylidene difluoride (PVDF) | 9.33 | 8.98 | 8.08 | 7.80 | 6.81 | 15.41 | 6.24 |
| | Binder solvent: NMP | 223.92 | 215.52 | 193.92 | 187.20 | 163.44 | 369.84 | 149.76 |
| | Current collector: Aluminium foil | 29.04 | 11.75 | 13.73 | 12.43 | 13.32 | 12.35 | 13.32 |
| Anode | Anode active material: Graphite | 235.14 | 200.34 | 210.14 | 201.21 | 201.36 | 208.67 | 204.73 |
| | Binder: PVDF | 4.80 | 4.09 | 4.20 | 4.11 | 4.11 | 4.26 | 4.18 |
| | Binder solvent: NMP | 115.20 | 98.16 | 100.80 | 98.64 | 98.64 | 102.24 | 100.32 |
| | Current collector: copper foil | 68.28 | 28.49 | 32.97 | 29.93 | 31.99 | 29.66 | 31.99 |
| Electrolyte | Electrolyte salt: $LiPF_6$ | 37.03 | 37.03 | 37.03 | 37.03 | 37.03 | 37.03 | 37.03 |
| | Electrolyte solvent: Ethylene carbonate (EC) | 51.57 | 24.27 | 22.95 | 20.43 | 19.59 | 19.23 | 18.33 |
| | Electrolyte solvent: Dimethyl carbonate (DMC) | 51.57 | 24.27 | 22.95 | 20.43 | 19.59 | 19.23 | 18.33 |
| Separator | Microporous membrane: Polypropylene (PP) | 7.36 | 2.91 | 3.42 | 3.84 | 3.32 | 3.75 | 3.33 |
| | Microporous membrane: Polyethylene (PE) | 1.84 | 0.73 | 0.86 | 0.96 | 0.83 | 0.94 | 0.83 |
| Positive terminal assembly | Tab: aluminium | 6.30 | 3.40 | 3.90 | 3.70 | 4.00 | 3.90 | 4.20 |
| Negative terminal assembly | Tab: copper | 20.70 | 11.10 | 13.00 | 12.30 | 13.30 | 12.80 | 13.90 |
| Cell container | Aluminium | 10.30 | 5.38 | 6.20 | 5.83 | 6.36 | 6.15 | 6.62 |
| | Polyethylene terephthalate (PET) | 1.60 | 0.84 | 0.96 | 0.91 | 0.99 | 0.96 | 1.03 |
| | PP | 0.69 | 0.36 | 0.41 | 0.39 | 0.42 | 0.41 | 0.44 |
| Output | Single LIB cell | 999.72 | 809.97 | 782.43 | 749.14 | 701.86 | 676.99 | 675.45 |
| | Recovered NMP | -332.34 | -307.41 | -288.83 | -280.12 | -256.84 | -462.64 | -245.08 |

## Table S3. Energy input and output of single LIB cell production (*21, 27*).

| Process | Energy component | LFP | LMO/ NMC532 | NMC333 | NMC532 | NMC622 | NMC811 | NCA |
|---|---|---|---|---|---|---|---|---|
| Cathode mixing, coating, and coiling | Electricity (kWh) | 7.47E-04 | 6.94E-04 | 6.29E-04 | 6.06E-04 | 5.33E-04 | 4.90E-04 | 4.90E-04 |
| Cathode drying | Heat (MJ) | 4.25E-01 | 3.81E-01 | 3.65E-01 | 3.52E-01 | 3.08E-01 | 5.83E-01 | 2.82E-01 |
| Anode mixing, coating, and coiling | Electricity (kWh) | 5.44E-04 | 4.12E-04 | 4.37E-04 | 4.16E-04 | 4.21E-04 | 4.29E-04 | 4.26E-04 |
| Anode drying | Heat (MJ) | 2.17E-01 | 1.81E-01 | 1.88E-01 | 1.82E-01 | 1.83E-01 | 1.89E-01 | 1.86E-01 |
| Drying of cathode, anode, and separator | Heat (MJ) | 1.11E-01 | 6.64E-02 | 9.21E-02 | 8.87E-02 | 8.17E-02 | 7.99E-02 | 7.81E-02 |
| Calendaring of cathode, anode, and separator | Electricity (kWh) | 2.08E-03 | 1.79E-03 | 1.72E-03 | 1.65E-03 | 1.53E-03 | 1.47E-03 | 1.46E-03 |
| Charging | Electricity (kWh) | 1.52E-01 | 1.52E-01 | 1.52E-01 | 1.52E-01 | 1.52E-01 | 1.52E-01 | 1.52E-01 |
| Dry room operation | Electricity (kWh) | 2.44E-01 | 1.98E-01 | 1.91E-01 | 1.83E-01 | 1.71E-01 | 1.65E-01 | 1.65E-01 |
| | Heat (MJ) | 1.65E+00 | 1.33E+00 | 1.29E+00 | 1.23E+00 | 1.16E+00 | 1.11E+00 | 1.11E+00 |
| Output | Single LIB cell (g) | 999.72 | 809.97 | 782.43 | 749.14 | 701.86 | 676.99 | 675.45 |

## Table S4. Material input and output of recovering 1 kg NMP (*21*).

| Input | | Amount |
|---|---|---|
| Material | NMP (g) | 1020.41 |
| | Decarbonised water (g) | 83297.91 |
| | Nitrogen (g) | 14.70 |
| Energy | Steam (g) | 1528.84 |
| | Electricity (kWh) | 0.05 |
| Output | NMP (g) | 1000.00 |

## Table S5. Material input and output of a single LIB module (*34*).

| Input | Component | LFP | LMO/NMC532 | NMC333 | NMC532 | NMC622 | NMC811 | NCA |
|---|---|---|---|---|---|---|---|---|
| Module | LIB cells (Number) | 12 | 12 | 12 | 12 | 12 | 12 | 12 |
| | Module casing: Aluminium (kg) | 0.22 | 0.14 | 0.15 | 0.15 | 0.16 | 0.15 | 0.16 |
| | Cooling plates: Aluminium (kg) | 0.22 | 0.10 | 0.12 | 0.11 | 0.13 | 0.12 | 0.13 |
| | Module terminals: Copper (kg) | 0.06 | 0.053 | 0.056 | 0.055 | 0.055 | 0.055 | 0.055 |
| Output | Single LIB module (kg) | 12.49 | 10.01 | 9.72 | 9.31 | 8.76 | 8.45 | 8.45 |

Material input and output of single Electric Vehicle LIB pack

The battery management system (BMS) is assumed to be 3% of the total mass of one battery pack (*29*). The energy input and output of the battery pack assembling processes, including material mixing, drying, NMP recovery, calendering, dry room operation, single-cell charge, welding, and testing, are estimated from Richa et al. (*21*).

**Table S6. Material input and output of single EV LIB pack.**

| Input | Component | LFP | LMO/ NMC532 | NMC333 | NMC532 | NMC622 | NMC811 | NCA |
|---|---|---|---|---|---|---|---|---|
| Module | LIB module (Number) (*34*) | 20 | 20 | 20 | 20 | 20 | 20 | 20 |
| Battery pack accessories | Outer layer of battery jacket: Aluminium (kg) (*34*) | 11.69 | 4.53 | 4.93 | 4.77 | 4.99 | 4.89 | 5.12 |
| | Sandwiched layer of battery jacket: Fibre glass (kg) (*34*) | 17.23 | 10.42 | 11.35 | 10.98 | 11.50 | 11.25 | 11.78 |
| | Module compression plates: Steel (kg) (*34*)<br>Straps: Steel (kg) (*34*) | 1.24 | 0.57 | 0.68 | 0.63 | 0.70 | 0.66 | 0.73 |
| | Module interconnects: Copper (kg) (*34*) | 1.46 | 1.28 | 1.34 | 1.32 | 1.32 | 1.32 | 1.32 |
| BMS | Performance monitoring: Printed wiring board (kg) (*29*) | 0.89 | 0.69 | 0.67 | 0.65 | 0.62 | 0.60 | 0.60 |
| | Wire: Copper (kg) (*29*) | 4.46 | 3.43 | 3.37 | 3.23 | 3.08 | 2.98 | 2.99 |
| | Corrosion resistance: Chromium steel 18/8 (kg) (*29*) | 3.57 | 2.75 | 2.70 | 2.59 | 2.46 | 2.38 | 2.39 |
| Coolant | Ethylene glycol (kg) (*34*) | 3.60 | 2.46 | 2.68 | 2.59 | 2.71 | 2.65 | 2.78 |
| | Deionised water (kg) (*34*) | 3.60 | 2.46 | 2.68 | 2.59 | 2.71 | 2.65 | 2.78 |
| Energy | Welding: Electricity (kWh) (*21, 79*) | 0.12 | 0.092 | 0.09 | 0.086 | 0.082 | 0.079 | 0.08 |
| | Test and activation: Electricity (kWh) (*21, 27*) | 36.37 | 36.37 | 36.39 | 36.37 | 36.40 | 36.40 | 36.38 |
| Output | Single EV LIB pack (kg) | 297.59 | 228.82 | 224.81 | 215.44 | 205.25 | 198.34 | 199.52 |

Material input and output of single Stationary Energy Storage Systems LIB pack

After retiring from the electric vehicle, battery cells will be tested to validate their residual energy capacity and this process consumes electricity (*21*). Life-extending battery cells are assumed to be repurposed into new battery packs with an energy storage capacity of 450 kWh, aiming for their second use in stationary ESS (*21*). All battery module components and part of battery pack components such as module compression plates and steep straps are reused, while coolant, BMS, module interconnects, and a tri-layer battery jacket is replaced. The replaced components will be sent to the EOL phase. Notably, BMS is assumed to be 1.5% of the total mass of one repurposed battery pack, based on the fact that BMS of battery packs designed for stationary ESS is not as demanding as those for EV, and thus less advanced printed wiring board design and less complicated wiring will be applied (*21*). Lastly, a cabinet made of mild steel sheet is used as the pack casing, instead of using the tri-layer jacket.

## Table S7. Material input and output of single 450 kWh stationary ESS LIB pack.

| Input | | Component | LFP | LMO/ NMC532 | NMC 333 | NMC 532 | NMC 622 | NMC 811 | NCA |
|---|---|---|---|---|---|---|---|---|---|
| EV LIB pack | Module | LIB cell (Number) | 2596 | 2596 | 2596 | 2596 | 2596 | 2596 | 2596 |
| | | Module casing: Aluminium (kg) | 47.16 | 29.85 | 33.10 | 31.80 | 33.75 | 32.67 | 34.61 |
| | | Cooling plates: Aluminium (kg) | 47.16 | 22.07 | 26.39 | 24.66 | 27.04 | 25.74 | 28.34 |
| | | Module terminals: Copper (kg) | 12.98 | 11.47 | 12.11 | 11.90 | 11.90 | 11.90 | 11.90 |
| | Battery pack accessories | Outer layer of battery jacket: Aluminium (kg) | 126.39 | 48.95 | 53.35 | 51.58 | 54.03 | 52.84 | 55.36 |
| | | Sandwiched layer of battery jacket: Fiber glass (kg) | 186.42 | 112.69 | 122.82 | 118.73 | 124.37 | 121.64 | 127.43 |
| | | Module compression plates: Steel (kg)<br>Straps: Steel (kg) | 13.37 | 6.11 | 7.32 | 6.83 | 7.52 | 7.18 | 7.92 |
| | | Module interconnects: Copper (kg) | 15.79 | 13.85 | 14.49 | 14.28 | 14.28 | 14.28 | 14.28 |
| | BMS | Performance monitoring: Printed wiring board (kg) | 9.66 | 7.43 | 7.30 | 6.99 | 6.66 | 6.44 | 6.47 |
| | | Wire: Copper (kg) | 48.28 | 37.13 | 36.48 | 34.96 | 33.30 | 32.18 | 32.37 |
| | | Corrosion resistance: Chromium steel 18/8 (kg) | 38.63 | 29.70 | 29.18 | 27.96 | 26.64 | 25.74 | 25.90 |
| | Coolant | Ethylene glycol (kg) | 38.89 | 26.55 | 28.99 | 27.96 | 29.31 | 28.66 | 30.07 |
| | | Deionised water (kg) | 38.89 | 26.55 | 28.99 | 27.96 | 29.31 | 28.66 | 30.07 |
| Battery pack accessories | | Module interconnects: Copper (kg) (*21*) | 15.79 | 13.85 | 14.49 | 14.28 | 14.28 | 14.28 | 14.28 |
| | | Steel cabinet: Mild steel (kg) (*21*) | 232.11 | 184.99 | 180.32 | 172.68 | 163.18 | 157.51 | 157.85 |
| BMS | | Performance monitoring: Printed wiring board (kg) (*21*, *29*) | 4.63 | 3.69 | 3.60 | 3.45 | 3.26 | 3.14 | 3.15 |
| | | Wire: Copper (kg) (*21*, *29*) | 23.16 | 18.46 | 17.99 | 17.23 | 16.28 | 15.72 | 15.75 |
| | | Corrosion resistance: Chromium steel 18/8 (kg) (*21*, *29*) | 18.53 | 14.77 | 14.39 | 13.78 | 13.03 | 12.57 | 12.60 |
| Coolant | | Ethylene glycol (kg) (*34*) | 38.89 | 26.55 | 28.99 | 27.96 | 29.31 | 28.66 | 30.07 |
| | | Deionised water (kg) (*34*) | 38.89 | 26.55 | 28.99 | 27.96 | 29.31 | 28.66 | 30.07 |
| Energy | | Welding: Electricity (kWh) (*21*, *79*) | 1.24 | 0.98 | 0.96 | 0.92 | 0.87 | 0.84 | 0.84 |
| | | Test and activation: Electricity (kWh) (*21*, *27*) | 4811.96 | 4811.96 | 4811.96 | 4811.96 | 4811.96 | 4811.96 | 4811.96 |
| Output | | ESS LIB pack (kg) | 3087.93 | 2461.03 | 2398.90 | 2297.31 | 2170.89 | 2095.49 | 2100.00 |
| Output waste | | Outer layer of battery jacket: Aluminium (kg) | 126.39 | 48.95 | 53.35 | 51.58 | 54.03 | 52.84 | 55.36 |
| | | Sandwiched layer of battery jacket: Fibre glass (kg) | 186.42 | 112.69 | 122.82 | 118.73 | 124.37 | 121.64 | 127.43 |
| | | Module interconnects: Copper (kg) | 15.79 | 13.85 | 14.49 | 14.28 | 14.28 | 14.28 | 14.28 |
| | | Performance monitoring: Printed wiring board (kg) | 9.66 | 7.43 | 7.30 | 6.99 | 6.66 | 6.44 | 6.47 |
| | | Wire: Copper (kg) | 48.28 | 37.13 | 36.48 | 34.96 | 33.30 | 32.18 | 32.37 |
| | | Corrosion resistance: Chromium steel 18/8 (kg) | 38.63 | 29.70 | 29.18 | 27.96 | 26.64 | 25.74 | 25.90 |
| | | Coolant (kg) | 77.77 | 53.11 | 57.98 | 55.92 | 58.63 | 57.33 | 60.14 |

## Cathode Active Material Production

The production of cathode active material for NMC and NCA LIBs requires material inputs of lithium source, transit metal sources, deionized water, ammonium hydroxide ($NH_4OH$), and sodium hydroxide (NaOH), energy inputs, and waste sludge treatment. In addition to the main products of NMC and NCA, sodium sulphate ($Na_2SO_4$) crystal is produced.

Fig. S3 depicts the process flow for NMC or NCA cathode active material production: First, nickel sulfate ($NiSO_4$), cobalt sulfate ($CoSO_4$), manganese sulfate ($MnSO_4$), or aluminum sulfate ($Al_2(SO_4)_3$) solutions in the case of NCA production, are dissolved and mixed. Then, 20% $NH_4OH$ and 48% NaOH are added to the mixed solution and heated to 50 °C, and the NMC or NCA precursor ($Ni_xMn_yCo_z(OH)_2$ or $Ni_{0.8}Co_{0.15}Al_{0.05}(OH)_2$) are subsequently precipitated as shown in Eqs. (1)–(5). Next, the NMC and NCA precursors are filtered out, washed with deionized water, and dried. The yields of products are assumed as 100% given that the amount of $NH_4OH$ and NaOH is slightly more than the amount calculated based on stoichiometry. The filtrate is further processed in an ammonia stripping tower to recover excess ammonia. After ammonia stripping, the processed filtrate is evaporated to recover $Na_2SO_4$ crystal (i.e., sodium and sulfate removal) and effluent is sent to the waste sludge treatment. Next, the assumed lithium source which is $Li_2CO_3$ for NMC33, NMC532, and NMC622, and lithium hydroxide (LiOH) for NMC811 and NCA as shown in Eqs. (3)–(5), respectively, are mixed with the dried cathode active material precursors and go through two-stage calcination (over 1000 °C) together over 12 hours to produce the cathode active materials. Particularly, the calcination process for NCA production requires oxygen input, while air ventilation is sufficient in the case of NMC production (*88*, *89*). Later, the product is crushed and sieved. In the end, the production is finalized with metal detection and demagnetization processes and preparation of ready-to-use NMC or NCA powder (*89*). Hydrothermal synthesis under 150–200 °C for 5 hours is assumed to be used to synthesize LFP (*29*). It is worth mentioning that some of the characterization factors for $NiSO_4$ are available from an analysis based on primary data covering 52% of global nickel production (*90*). However, due to the incomplete geographical scope of the Nickel Institute analysis and the inaccessible characterization factors for some midpoint indicators adopted in this study, we collect the LCIA result of $NiSO_4$ from the Ecoinvent database.

Heat and electricity are exclusively consumed to power the precursor production and the calcination process, respectively. Heat production from natural gas burned at the industrial furnace is required for precipitation, ammonia stripping, and evaporation, among which the most demanding process is precipitation, followed by evaporation. Since energy demand for calcination of materials rich in nickel (Ni) is the most intensive, calcination for NCA and NMC811 is assumed to consume more electricity (8 kWh/kg cathode active materials produced) compared to that of the less Ni-containing NMC622, NMC532, and NMC333 (7 kWh/kg cathode active materials produced). Table S8 details the bill of materials and energy.

$$xNiSO_4 + yMnSO_4 + zCoSO_4 + 2NaOH \rightarrow Ni_xMn_yCo_z(OH)_2 + Na_2SO_4 \quad (1)$$

$$NiSO_4 + CoSO_4 + Al_2(SO_4)_3 + NaOH \rightarrow Ni_{0.8}Co_{0.15}Al_{0.05}(OH)_2 + Na_2SO_4 \quad (2)$$

$$Ni_xMn_yCo_z(OH)_2 + 0.5Li_2CO_3 + 0.25O_2 + H_2O \rightarrow LiNi_xMn_yCo_zO_2 + 2H_2O + 0.5CO_2 \quad (3)$$

$$Ni_{0.8}Mn_{0.1}Co_{0.1}(OH)_2 + LiOH \cdot H_2O + 0.25O_2 \rightarrow LiNi_{0.8}Mn_{0.1}Co_{0.1}O_2 + 2.5H_2O \quad (4)$$

$$Ni_{0.8}Co_{0.15}Al_{0.05}(OH)_2 + LiOH \cdot H_2O + 0.25O_2 \rightarrow LiNi_{0.8}Co_{0.15}Al_{0.05}O_2 + 2.5H_2O \quad (5)$$

$$3LiOH + H_3PO_4 + FeSO_4 \rightarrow LiFePO_4 + Li_2SO_4 + 3H_2O \quad (6)$$

The cradle-to-gate LCI data for $CoSO_4$ and $CoCl_2$ production is obtained from the GREET model following the mass allocation, as shown in Table S8 (*91*). The amount of P204 and P507 are considered negligible as suggested by the GREET model and thus not included in the LCI (*91*).

**Table S8. Material input and output of $CoSO_4$ and $CoCl_2$ production (*91*).**

| **Input** | Unit | Quantity for $CoSO_4$ production | Quantity for $CoCl_2$ production |
|---|---|---|---|
| Diesel | MJ | 8.65 | 10.33 |
| Electricity | kWh | 1.9 | 8.16 |
| Natural gas | MJ | 10.77 | 12.85 |
| Water | kg | 34.33 | 40.98 |
| Sulfur | kg | 0.27 | 0.33 |
| Limestone | kg | 0.38 | 0.45 |
| Lime | kg | 0.14 | 0.17 |
| NaOH | kg | 1.056 | 1.26 |
| MgO | kg | 0.45 | 0.53 |
| $H_2SO_4$ | kg | 0.98 | 1.17 |
| HCl | kg | 0.54 | 0.64 |
| Kerosene | kg | 0.018 | 0.021 |
| $Na_2S_5O_5$ | kg | 0.03 | 0.036 |
| $NH_4HCO_3$ | kg | 0.22 | 0.26 |
| $Na_2CO_3$ | kg | 0.034 | 0.04 |
| PM10 | kg | 0.021 | 0.025 |
| PM2.5 | kg | 0.0022 | 0.0026 |
| $SO_2$ | kg | 0.0025 | 0.003 |
| Output ($CoSO_4$) | kg | 1.00 | 1.00 |

**Table S9. Material input and output of cathode active material production for LFP, NMC333, NMC532, NMC622, NMC811, and NCA LIBs.**

| Input | Component | LFP (*29*) | NMC333 (*89*) | NMC532 (*89*) | NMC622 (*89*) | NMC811 (*89*) | NCA (*88*) |
|---|---|---|---|---|---|---|---|
| Lithium source | $Li_2CO_3$ (kg) | - | 0.38 | 0.38 | 0.38 | - | - |
| | LiOH (kg) | 0.46 | - | - | - | 0.25 | 0.25 |
| Precursor | $MnSO_4$ (kg) | - | 0.50 | 0.49 | 0.33 | 0.15 | - |
| | $NiSO_4$ (kg) | - | 0.51 | 0.84 | 1.01 | 1.27 | 1.29 |
| | $CoSO_4$ (kg) | - | 0.51 | 0.34 | 0.34 | 0.16 | 0.25 |
| | $Al_2(SO_4)_3$ (kg) | - | - | - | - | - | 0.086 |
| | $FeSO_4$ (kg) | 0.96 | - | - | - | - | - |
| | $H_3PO_4$ (kg) | 0.62 | - | - | - | - | - |
| | NaOH (kg) | - | 0.87 | 0.87 | 0.87 | 0.87 | 0.84 |
| | Ammonia ($NH_3$) | - | 0.06 | 0.06 | 0.06 | 0.06 | 0.35 |
| Oxygen | Oxygen (kg) | - | - | - | - | - | 0.04 |
| Water | Decarbonised Water (kg) | 46 | 0.64 | 0.64 | 0.64 | 0.64 | 0.64 |
| Energy | Electricity (kWh) | - | 6.35 | 6.35 | 6.35 | 7.26 | 7.26 |
| | Heat (MJ) | 15 | 38.67 | 38.67 | 38.67 | 38.67 | 38.67 |
| Output | Cathode active material (kg) | 1.00 | 1.00 | 1.00 | 1.00 | 1.00 | 1.00 |
| Emitted (kg) | $CO_2$ | - | 0.23 | 0.23 | 0.23 | - | - |
| Avoided | $Na_2SO_4$ (kg) | - | 1.55 | 1.55 | 1.54 | 1.54 | 1.48 |

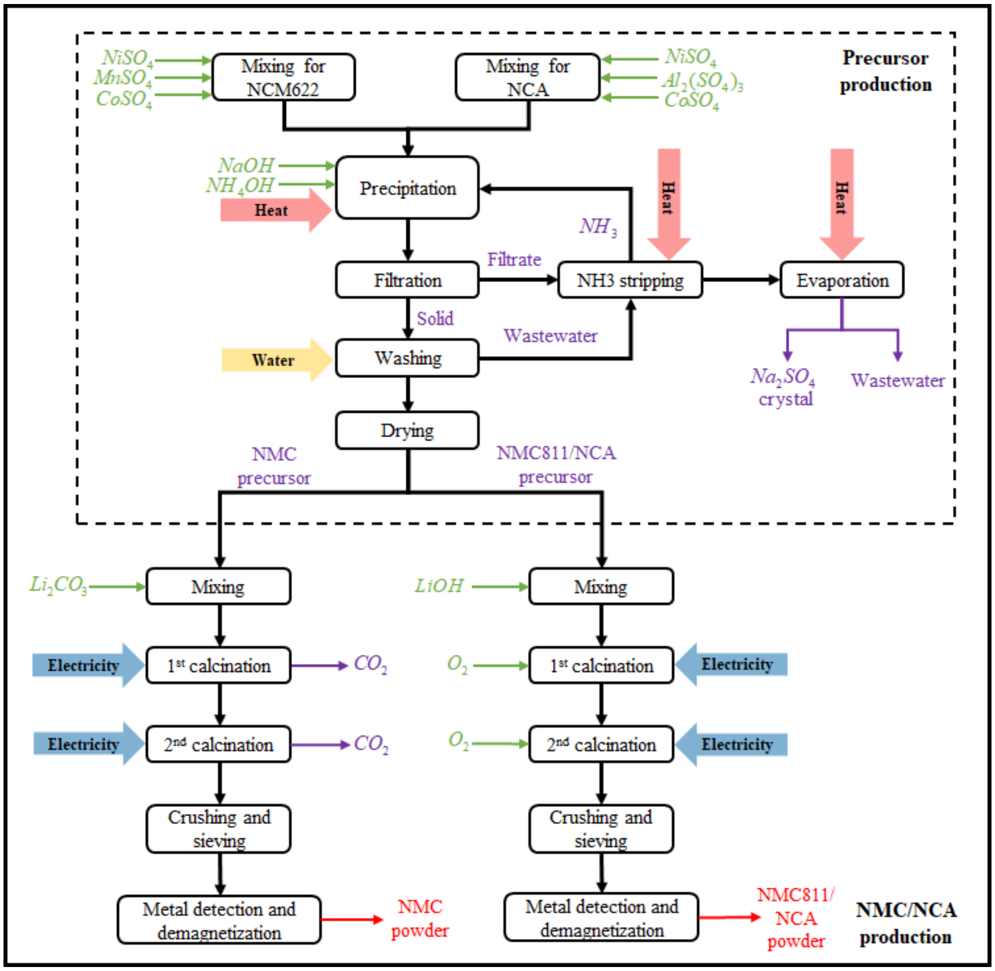


**Fig. S3. Process diagram of active material production for NMC and NCA LIBs.**

**Recycling Processes**

Three EOL scenarios, namely hydrometallurgical, pyrometallurgical and direct cathode recycling, are investigated and the processes are depicted in Figs. S4–S6. Notably, for the EOL scenarios of hydrometallurgical recycling and direct cathode recycling, the spent LIB pack is first discharged and dismantled to the cell level (i.e., all battery module and pack components, including BMS, coolant, terminals, interconnects, thermal systems, and casing were manually disassembled and disposed of accordingly), while the spent LIB pack is dismantled to the module level for pyrometallurgical recycling. Then, the battery cells are further dismantled to the cathode, anode, electrolyte, separator, positive and negative terminal assemblies, and cell container. In this step, the plastics (PP, PE, and PET) constituting the separator and cell container are landfilled. The metals such as aluminum (composing current collector for the cathode, positive terminal assembly, cell container, inner and outer layer of battery pack jacket, module casing, and cooling plate) and copper (composing current collector for the anode, negative terminal assembly, interconnects, module terminals, the wires in BMS) are assumed to be recycled to produce (including collection, sorting, cleaning, pressing and re-melting for aluminum, and collection, refining and wastewater management for copper, respectively) with a loss rate of 5%, 1.3%, and 8%, respectively. The waste steel is landfilled, waste chromium steel is recycled, and waste fiber glass is sent to the municipal incinerator. The printed wiring boards are weighted, separated, and shredded into electronic scraps that are ready for further metallurgical treatment (*92*). The coolant is incinerated as hazardous waste. After disassembly, cathode, anode, and electrolyte are left for all three EOL scenarios. In addition, plastics (PP, PE, and PET), aluminum and copper, are left for the scenario of pyrometallurgical recycling. Due to the lack of information, the waste sludge treatment of the EOL phase of spent LIBs is replaced by treatment of sludge from steel rolling, given that sludge from steel rolling resembles sludge from the treatment of spent LIBs.

Hydrometallurgical Recycling

Hydrometallurgical recycling aims at recovering metals using aqueous chemistry, which involves steps of leaching, solvent extraction, and precipitation. Fig. S4 depicts the process design of hydrometallurgical recycling. Tables S9–S10 detail the bill of materials and energy. Under this scenario, the leftovers are soaked in NMP at 100 °C for one hour and crushed in a planetary ball mill to dissolve the binder PVDF and separate the active materials from the current collectors in the cathode and anode, respectively (*93*). The NMP soaking needs inputs of NMP, deionized water, nitrogen, and heat. The crushing step requires electricity input. The amount of NMP needed is assumed to be the same as the sum of the weight of active materials, graphite, carbon black, and PVDF (*93*). The heat needed to support NMP soaking ($Q_{soaking}$) is calculated as shown in Eq. (7), where $eff_{ng}$, $c_p^i$, $m_i$ and ΔT represent the efficiency of natural gas boilers (60%), the specific heat for each soaked material i (2100, 900, 385 J/(kg·°C) for NMP, aluminum, and copper, active materials for both cathode and anode), the mass of each soaked material i, and the elevated temperature (75 °C in premise of an assumed room temperature of 25 °C).

$$Q_{\text{soaking}} = \frac{\sum_i c_p^i \cdot m_i \cdot \Delta T}{eff_{\text{ng}}}, i \in \{\text{Al,Cu,NMP, PVDF, graphite, carbon black}\} \tag{7}$$

Later, solids such as graphite, carbon black, copper, and aluminum are filtered out. The carbon black and graphite are completely combusted to $CO_2$. The copper and aluminum are recycled, and the filtrate is calcined at around 700 °C with inputs of electricity and heat. During the calcination, NMP vapor is recovered for the following reasons: (1) large environmental impacts if NMP vapor is emitted (2) costly expense of NMP (3) easy NMP recovery approach by cooling down to or below room temperature due to its high boiling point (204 °C) and low vapor pressure. To be specific, the NMP vapor can be cooled down using deionized water paired up with a nitrogen stripping system to remove the dissolved oxygen (*21, 94*). The high boiling point and low vapor pressure ensure the stability of NMP at room temperature (*95*). A recovery rate of 98% is assumed for NMP recovery (*96*). The PVDF is burned out and is assumed to be treated the same as municipal incineration. Next, the cathode active material is ground, and the leaching process is used to extract the valuable metals from the aged lithium-metal-oxides using heat, hydrogen peroxide ($H_2O_2$), and a variety of acids. Specifically, $H_2O_2$ and citric acid (10% more input than calculated based on stoichiometry and a recovery rate of 90%) are used for leaching lithium (Li) ions from aged LMO; 1 vol.% $H_2O_2$, 1 M sulfuric acid ($H_2SO_4$) is used for leaching Ni, cobalt (Co), and manganese (Mn) ions from aged NMC at 40 °C with a stirring speed of 400 rpm and solid-to-liquid (S:L) ratio of 40 g/L for one hour (*97*). Moreover, a minor amount of hydrochloric acid (HCl) is added. $H_2O_2$ and 4 M HCl are used for leaching Ni, Co, and aluminum (Al) ions from aged NCA at 80 °C with an S:L ratio of 100 g/L for 80 minutes. 0.5 M phosphoric acid solution is used for leaching $FePO_4$ and Li at room temperature with an S:L ratio of 25 g/L under continuous stirring for 1 hour. The leaching efficiency is 95% of $FePO_4$ for LFP, 95% of Li for LMO, 99.7% of Li, Ni, Co, Mn for NMC, and 80% of Li, 99.8% of Ni, 95.6% of Co, and 99.5% of Al for NCA, respectively. After leaching, filtration and reflux heating at 85 °C for 9 hours are performed to obtain $FePO_4 \cdot 2H_2O$.

In the precipitation step, sodium carbonate ($Na_2CO_3$) is added to the leachate of aged cathode active materials to recover $Li_2CO_3$. In addition to $Na_2CO_3$, NaOH and $NH_4OH$ are added to the

leachate of aged LMO/NMC532, NMC, or NCA to precipitate their precursors and $Li_2CO_3$. Inputs of heat, electricity and deionized water are required for precipitation. Then, manganese (III) oxide ($Mn_2O_3$) goes through the second calcination together with $Li_2CO_3$ to produce cathode active materials of LMO and emit $CO_2$. Flows of oxygen and nitrogen are also introduced to the kiln. NMC and NCA go through the second calcination with $Li_2CO_3$ and LiOH, respectively. $FePO_4·2H_2O$ is mixed with $Li_2CO_3$ (molar ratio of 1:1.05) and glucose (20 wt.% of LFP product) in water. The slurry is dried, milled, and heated at 350 ℃ for 4 hours and at 650 ℃ for 9 hours in a nitrogen atmosphere. The second calcination of NMC emits $CO_2$. In the final step, the cathode active materials are crushed, sieved, and undergone metal detection and demagnetization to produce cathode active material powder.

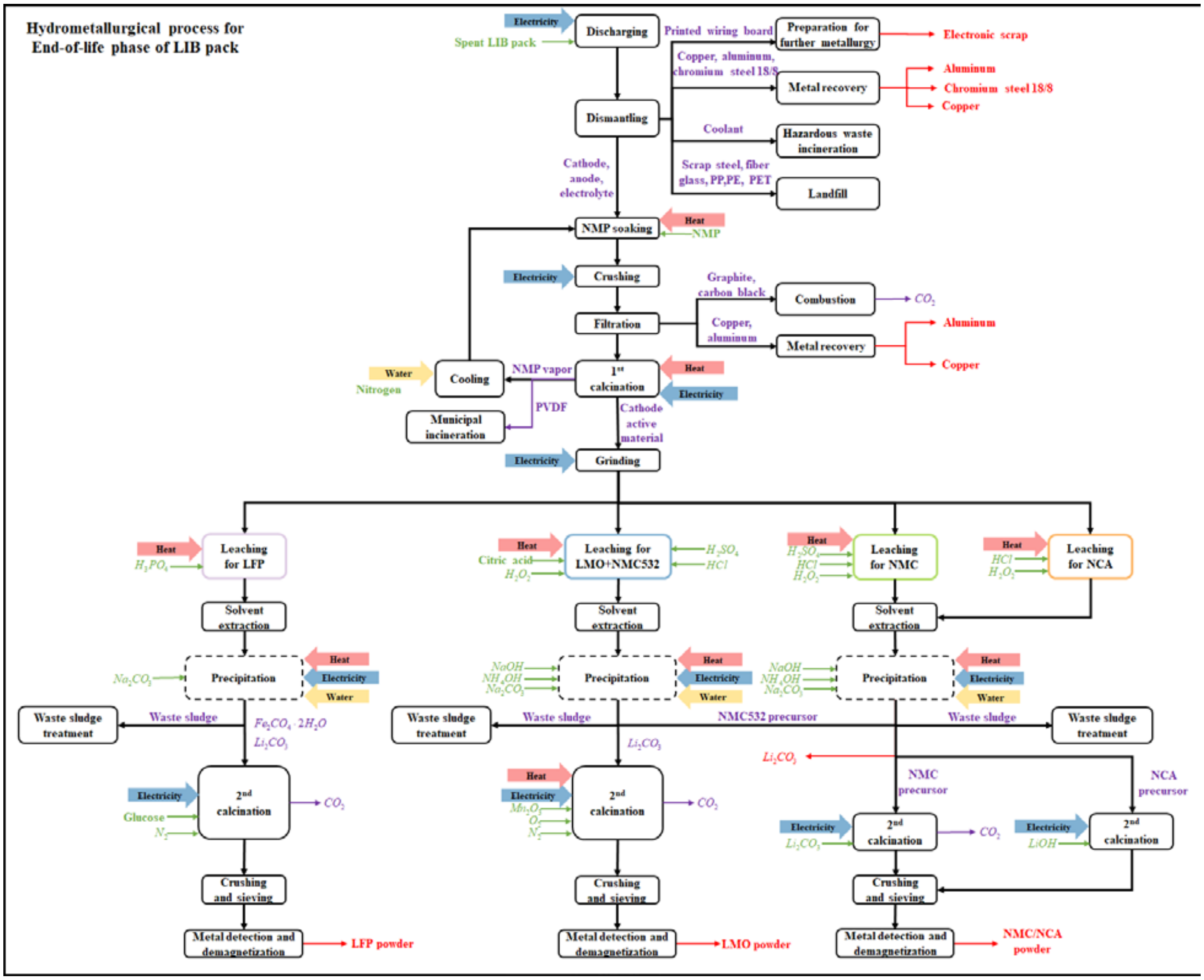


**Fig. S4. Process diagram of the hydrometallurgical recycling.**

**Table S10. Material input and output of hydrometallurgical recycling for EOL LIB packs with an initial energy storage capacity of 562.5 kWh (LFP, LMO/NMC532, NMC622, NCA).**

| | | After EV use | | | | After cascaded use | | | |
|---|---|---|---|---|---|---|---|---|---|
| | | LFP | LMO/ NMC | NMC622 | NCA | LFP | LMO/ NMC | NMC622 | NCA |
| Material input (kg) | EV LIB pack | 3218.89 | 2475.02 | 2220.15 | 2158.19 | - | - | - | - |
| | ESS LIB pack | - | - | - | - | 3087.93 | 2461.03 | 2170.89 | 2100.00 |
| | Waste aluminum | - | - | - | - | 126.39 | 48.95 | 54.03 | 55.36 |
| | Waste fibre glass | - | - | - | - | 186.42 | 112.69 | 124.37 | 127.43 |
| | Waste copper | - | - | - | - | 64.08 | 50.97 | 47.58 | 46.65 |
| | Waste printed wiring board | - | - | - | - | 9.66 | 7.43 | 6.66 | 6.47 |
| | Waste steel | - | - | - | - | 38.63 | 29.70 | 26.64 | 25.90 |
| | Waste coolant | - | - | - | - | 77.77 | 53.11 | 58.63 | 60.14 |
| | NMP (*93*) | 1834.54 | 1695.68 | 1417.13 | 1352.78 | 1834.54 | 1695.68 | 1417.13 | 1352.78 |
| | Decarbonised water (*21*) | 148165.86 | 136951.84 | 114454.37 | 109256.35 | 148165.86 | 136951.84 | 114454.37 | 109256.3 5 |
| | Nitrogen (*21*) | 31.01 | 28.66 | 23.95 | 22.86 | 31.01 | 28.66 | 23.95 | 22.86 |
| | Steam (*21*) | 2728.60 | 2522.08 | 2107.77 | 2012.05 | 2728.60 | 2522.08 | 2107.77 | 2012.05 |
| | Citric acid (*93*) | - | 1297.29 | - | - | - | 1297.29 | - | - |
| | $Mn_2O_3$ (*27*) | - | 416.55 | - | - | - | 416.55 | - | - |
| | $O_2$ (*27*) | - | 324.44 | - | - | - | 324.44 | - | - |
| | $N_2$ (*27, 98*) | 934.63 | 356.66 | - | - | 934.63 | 356.66 | - | - |
| | $H_2SO_4$ (*97*) | - | 1369.99 | 2070.03 | - | - | 1369.99 | 2070.03 | - |
| | HCl (*39, 99*) | - | 43.37 | 33.71 | 1711.70 | - | 43.37 | 33.71 | 1711.70 |
| | $H_3PO_4$ (*98*) | 2279.90 | - | - | - | 2279.90 | - | - | - |
| | $H_2O_2$ (*93*) | - | 357.87 | 313.90 | - | - | 357.87 | 313.90 | - |
| | $NH_3$ (*99*) | - | 28.29 | 42.92 | 37.74 | - | 28.29 | 42.92 | 37.74 |
| | NaOH (*99*) | - | 1042.79 | 1582.10 | 1391.29 | - | 1042.79 | 1582.10 | 1391.29 |
| | Sodium carbonate ($Na_2CO_3$) (*31, 93, 100*) | 389.75 | 470.68 | 463.89 | 468.58 | 389.75 | 470.68 | 463.89 | 468.58 |
| | $Li_2CO_3$ (*27, 98, 99*) | 271.65 | 322.23 | 340.87 | - | 271.65 | 322.23 | 340.87 | - |
| | LiOH (*88*) | - | - | - | 185.95 | - | - | - | - |
| | Glucose (*98*) | 237.82 | - | - | - | 237.82 | - | - | - |
| | Deionised water (*27, 99*) | 15243.83 | 14290.55 | 11944.59 | 8996.73 | 15243.83 | 14290.55 | 11944.59 | 8996.73 |
| Energy input (kg) | Electricity: Discharging (kWh) (*93*) | 47.37 | 34.88 | 34.38 | 31.70 | 47.37 | 34.88 | 34.38 | 31.70 |
| | Electricity: Anode crushing (kWh) (*93*) | 26.87 | 20.33 | 20.72 | 21.43 | 26.87 | 20.33 | 20.72 | 21.43 |
| | Electricity: Cathode crushing (kWh) (*93*) | 43.29 | 40.22 | 30.89 | 28.99 | 43.29 | 40.22 | 30.89 | 28.99 |
| | Heat: NMP soaking (MJ) (*93*) | 439.51 | 401.00 | 336.36 | 321.30 | 439.51 | 401.00 | 336.36 | 321.30 |
| | Electricity: Calcination (kWh) (*93*) | 29.13 | 27.97 | 21.28 | 67.00 | 29.13 | 27.97 | 21.28 | 67.00 |
| | Heat: Calcination (MJ) (*93*) | 2634.01 | 2529.67 | 1924.47 | 1767.13 | 2634.01 | 2529.67 | 1924.47 | 1767.13 |

| | | | | | | | | | |
|---|---|---|---|---|---|---|---|---|---|
| | Electricity: Grinding (kWh) (*93*) | 4.52 | 4.35 | 3.30 | 3.02 | 4.52 | 4.35 | 3.30 | 3.02 |
| | Heat: Leaching (MJ) (*93*) | 16.29 | 15.66 | 11.88 | 10.87 | 16.29 | 15.66 | 11.88 | 10.87 |
| | Electricity: Precipitation (kWh) (*101*) | 87.43 | 80.86 | 62.85 | 55.27 | 87.43 | 80.86 | 62.85 | 55.27 |
| | Heat: Precipitation (MJ) (*101*) | 20827.97 | 19261.32 | 14972.86 | 13166.99 | 20827.97 | 19261.32 | 14972.86 | 13166.99 |
| | Electricity: 2nd calcination (kWh) (*27*, *101*) | 8093.73 | 3837.31 | 5818.44 | 5116.68 | 8093.73 | 3837.31 | 5818.44 | 5116.68 |
| | Heat: 2nd calcination (MJ) (*27*) | - | 6942.53 | - | - | - | 6942.53 | - | - |
| Output waste (kg) | Waste copper | 2.41 | 1.48 | 1.55 | 1.56 | 2.92 | 1.90 | 1.95 | 1.95 |
| | Waste aluminium | 16.96 | 7.71 | 8.82 | 9.05 | 16.96 | 7.71 | 8.82 | 9.05 |
| | Waste printed wiring board | 9.66 | 7.43 | 6.66 | 6.47 | 14.29 | 11.12 | 9.92 | 9.62 |
| | Waste chromium steel 18/8 | 3.09 | 2.38 | 2.13 | 2.07 | 4.57 | 3.56 | 3.17 | 3.08 |
| | Waste coolant | 77.77 | 53.11 | 58.63 | 60.14 | 155.54 | 106.22 | 117.25 | 120.28 |
| | Waste fibre glass | 186.42 | 112.69 | 124.37 | 127.43 | 186.42 | 112.69 | 124.37 | 127.43 |
| | Waste steel | 13.37 | 6.11 | 7.52 | 7.92 | 245.48 | 191.10 | 170.70 | 165.77 |
| | Waste PP | 20.88 | 8.47 | 9.71 | 9.78 | 20.88 | 8.47 | 9.71 | 9.78 |
| | Waste PE | 4.77 | 1.89 | 2.15 | 2.16 | 4.77 | 1.89 | 2.15 | 2.16 |
| | Waste PET | 4.16 | 2.17 | 2.57 | 2.67 | 4.16 | 2.17 | 2.57 | 2.67 |
| | Waste sludge | 18573.66 | 19141.90 | 16644.99 | 12906.23 | 19508.29 | 19141.90 | 16644.99 | 12906.23 |
| | Waste PVDF | 36.68 | 33.93 | 28.35 | 27.05 | 36.68 | 33.93 | 28.35 | 27.05 |
| Avoided material (kg) | Chromium steel 18/8 (*102*) | 35.54 | 27.32 | 24.51 | 23.83 | 52.58 | 40.91 | 36.49 | 35.42 |
| | Aluminium (*103*) | 322.23 | 146.45 | 167.49 | 171.95 | 322.23 | 146.45 | 167.49 | 171.95 |
| | Copper (*104*) | 305.63 | 163.74 | 175.49 | 176.11 | 344.07 | 195.62 | 205.65 | 205.75 |
| | Cathode active material (*39*, *97*) | 1189.10 | - | 845.83 | 743.81 | 1189.10 | - | 845.83 | 743.81 |
| | NMC532 (from LMO/NMC532) (*97*) | - | 557.50 | - | - | - | 557.50 | - | - |
| | LMO (from LMO/NMC532) | - | 453.76 | - | - | - | 453.76 | - | - |
| | Citric acid (*93*) | - | 1167.56 | - | - | - | 1167.56 | - | - |
| | $Li_2CO_3$ (*39*, *93*, *97*) | 204.25 | 322.23 | 322.87 | 261.70 | 204.25 | 322.23 | 322.87 | 261.70 |
| | NMP (*96*) | 1815.11 | 1677.73 | 1402.12 | 1338.44 | 1815.11 | 1677.73 | 1402.12 | 1338.44 |
| Emitted (kg) | $CO_2$ (*27*, *97*, *99*) | 2488.81 | 2165.35 | 2155.70 | 2008.15 | 2488.81 | 2165.35 | 2155.70 | 2008.15 |
| | $N_2$ | 934.63 | 356.66 | - | - | 934.63 | 356.66 | - | - |

**Table S11. Material input and output of hydrometallurgical recycling for EOL LIB packs with an initial energy storage capacity of 562.5 kWh (NMC333, NMC532, NMC811).**

| | | After EV use | | | After cascaded use | | |
|---|---|---|---|---|---|---|---|
| | | NMC333 | NMC532 | NMC811 | NMC333 | NMC532 | NMC811 |
| Material input (kg) | EV LIB pack | 2431.72 | 2330.39 | 2145.40 | - | - | - |
| | ESS LIB pack | - | - | - | 2398.90 | 2297.31 | 2095.49 |
| | Waste aluminum | - | - | - | 53.35 | 51.58 | 52.84 |
| | Waste fibre glass | - | - | - | 122.82 | 118.73 | 121.64 |
| | Waste copper | - | - | - | 50.97 | 49.23 | 46.46 |
| | Waste printed wiring board | - | - | - | 7.30 | 6.99 | 6.44 |
| | Waste steel | - | - | - | 29.18 | 27.96 | 25.74 |
| | Waste coolant | - | - | - | 57.98 | 55.92 | 57.33 |
| | NMP (*93*) | 1604.77 | 1545.32 | 1352.91 | 1604.77 | 1545.32 | 1352.91 |
| | Decarbonised water (*21*) | 129600.44 | 124807.87 | 110188.79 | 129600.44 | 124807.87 | 110188.79 |
| | Nitrogen (*21*) | 27.12 | 26.12 | 23.06 | 27.12 | 26.12 | 23.06 |
| | Steam (*21*) | 2386.70 | 2298.44 | 2029.22 | 2386.70 | 2298.44 | 2029.22 |
| | $H_2SO_4$ (*97*) | 2465.66 | 2380.94 | 1749.92 | 2465.66 | 2380.94 | 1749.92 |
| | HCl (*99*) | 39.99 | 38.62 | 28.62 | 39.99 | 38.62 | 28.62 |
| | $H_2O_2$ (*93*) | 372.36 | 359.57 | 266.45 | 372.36 | 359.57 | 266.45 |
| | $NH_3$ (*99*) | 50.91 | 49.16 | 36.43 | 50.91 | 49.16 | 36.43 |
| | NaOH (*99*) | 1876.79 | 1812.30 | 1342.95 | 1876.79 | 1812.30 | 1342.95 |
| | Sodium carbonate ($Na_2CO_3$) (*31*) | 552.96 | 533.45 | 392.10 | 552.96 | 533.45 | 392.10 |
| | $Li_2CO_3$ (*27*, *99*) | 404.36 | 390.47 | 289.34 | 404.36 | 390.47 | 289.34 |
| | Deionised water (*99*) | 14169.39 | 13682.55 | 10139.01 | 14169.39 | 13682.55 | 10139.01 |
| Energy input (kg) | Electricity: Discharging (kWh) (*93*) | 40.98 | 39.53 | 28.98 | 40.98 | 39.53 | 28.98 |
| | Electricity: Anode crushing (kWh) (*93*) | 21.58 | 20.53 | 21.17 | 21.58 | 20.53 | 21.17 |
| | Electricity: Cathode crushing (kWh) (*93*) | 36.47 | 35.14 | 28.38 | 36.47 | 35.14 | 28.38 |
| | Heat: NMP soaking (MJ) (*93*) | 380.40 | 366.05 | 322.80 | 380.40 | 366.05 | 322.80 |
| | Electricity: Calcination (kWh) (*93*) | 25.21 | 24.34 | 19.30 | 25.21 | 24.34 | 19.30 |
| | Heat: Calcination (MJ) (*93*) | 2279.72 | 2201.19 | 1745.39 | 2279.72 | 2201.19 | 1745.39 |
| | Electricity: Grinding (kWh) (*93*) | 3.91 | 3.78 | 2.80 | 3.91 | 3.78 | 2.80 |
| | Heat: Leaching (MJ) (*93*) | 14.09 | 13.61 | 10.08 | 14.09 | 13.61 | 10.08 |
| | Electricity: Precipitation (kWh) (*101*) | 74.56 | 72.00 | 53.35 | 74.56 | 72.00 | 53.35 |
| | Heat: Precipitation (MJ) (*101*) | 17761.71 | 17151.44 | 12709.52 | 17761.71 | 17151.44 | 12709.52 |
| | Electricity: $2^{nd}$ calcination (kWh) (*27*, *101*) | 6902.19 | 6665.03 | 4938.91 | 6902.19 | 6665.03 | 4938.91 |
| Output waste (kg) | Waste copper | 1.60 | 1.52 | 1.50 | 2.02 | 1.93 | 1.89 |
| | Waste aluminium | 8.73 | 8.25 | 8.47 | 8.73 | 8.25 | 8.47 |
| | Waste printed wiring board | 7.30 | 6.99 | 6.44 | 10.89 | 10.44 | 9.58 |
| | Waste chromium steel 18/8 | 2.33 | 2.24 | 2.06 | 3.49 | 3.34 | 3.07 |
| | Waste coolant | 57.98 | 55.92 | 57.33 | 115.95 | 111.84 | 114.66 |
| | Waste fibre glass | 122.82 | 118.73 | 121.64 | 122.82 | 118.73 | 121.64 |
| | Waste steel | 7.32 | 6.83 | 7.18 | 183.80 | 179.51 | 164.69 |
| | Waste PP | 9.95 | 10.97 | 10.80 | 9.95 | 10.97 | 10.80 |
| | Waste PE | 2.22 | 2.49 | 2.43 | 2.22 | 2.49 | 2.43 |
| | Waste PET | 2.50 | 2.35 | 2.48 | 2.50 | 2.35 | 2.48 |
| | Waste sludge | 19717.95 | 19033.48 | 14175.13 | 19717.95 | 19033.48 | 14175.13 |
| | Waste PVDF | 31.88 | 30.92 | 51.06 | 31.88 | 30.92 | 51.06 |
| Avoided material (kg) | Chromium steel 18/8 (*102*) | 26.85 | 25.73 | 23.69 | 40.09 | 38.41 | 35.25 |
| | Aluminium (*103*) | 165.96 | 156.80 | 160.94 | 165.96 | 156.80 | 160.94 |
| | Copper (*104*) | 180.83 | 169.23 | 167.08 | 212.89 | 200.33 | 196.68 |
| | Cathode active material (*97*) | 1003.37 | 968.90 | 717.97 | 1003.37 | 968.90 | 717.97 |
| | $Li_2CO_3$ (*97*) | 384.87 | 371.29 | 272.91 | 384.87 | 371.29 | 272.91 |

| | | | | | | | |
|---|---|---|---|---|---|---|---|
| | NMP (*96*) | 1587.67 | 1528.96 | 1349.87 | 1587.67 | 1528.96 | 1349.87 |
| Emitted (kg) | $CO_2$ (*97, 99*) | 2283.81 | 2189.05 | 2280.81 | 2283.81 | 2189.05 | 2280.81 |

Direct Cathode Recycling

Fig. S5 depicts the process design of direct cathode recycling. Tables S11– S12 detail the bill of materials and energy. The direct cathode recycling process focuses on recovering the cathode active material and electrolyte with limited processing. The discharging and dismantling are assumed to be the same as described in that of hydrometallurgical recycling. After dismantling, cathode, anode, and electrolyte are crushed first. Electricity is used in this step. Then the electrolyte is extracted by liquid $CO_2$ at 25 °C and 60 bar with a flow rate of 15 L/min, additional solvent, namely acetonitrile (ACN) and propylene carbonate (PC) (3:1), are added to the liquid $CO_2$ stream with a flow rate of 0.5 mL/min (*41*). This process is assumed to be operated in a dry room supported by both heat and electricity (*41, 93*). An experiment showed that the recovery rate of lithium hexafluorophosphate ($LiPF_6$), EC, and DMC were 100%, 75.76%, and 81.71%, respectively. The extraction time varies according to the amount of electrolyte and the ratio of $LiPF_6$, EC, and EMC (*41*). We assume no loss for ACN, PC, and $CO_2$. After the electrolyte is extracted and recovered, the residues undergo steps of size reduction and final separation which both require electricity (*93*). Then, they are soaked in NMP to dissolve PVDF as described in the hydrometallurgical recycling, so do the NMP recovery, the combustion of graphite and carbon black, the recovery of copper and aluminum, and the municipal incineration of PVDF. The next steps for aged cathode active material of LMO/NMC532, NMC, and NCA are hydrothermal treatment with LiOH and annealing process. During the hydrothermal process, the aged cathode active material is put in LiOH solution at 220 °C for four hours with a volume ratio of solid to liquid equals to 1:1 (*40*). The amount of LiOH depends on the cyclable lithium-ion loss, which is assumed to be 10% after first use and 20% after cascaded use (*105*). A 5% (molar) excess of lithium ions than needed is assumed to be applied. The residues are washed, dried, and annealed at 800 °C for four hours. The energy input for hydrothermal treatment and annealing is assumed to be 1.59 MJ/kg and 4.29 MJ/kg aged cathode active material (*40*). For LFP, this step is called solid-phase sintering which directly regenerates LFP at 700 °C with doping of new LFP at a ratio of 3:7 (*42*).

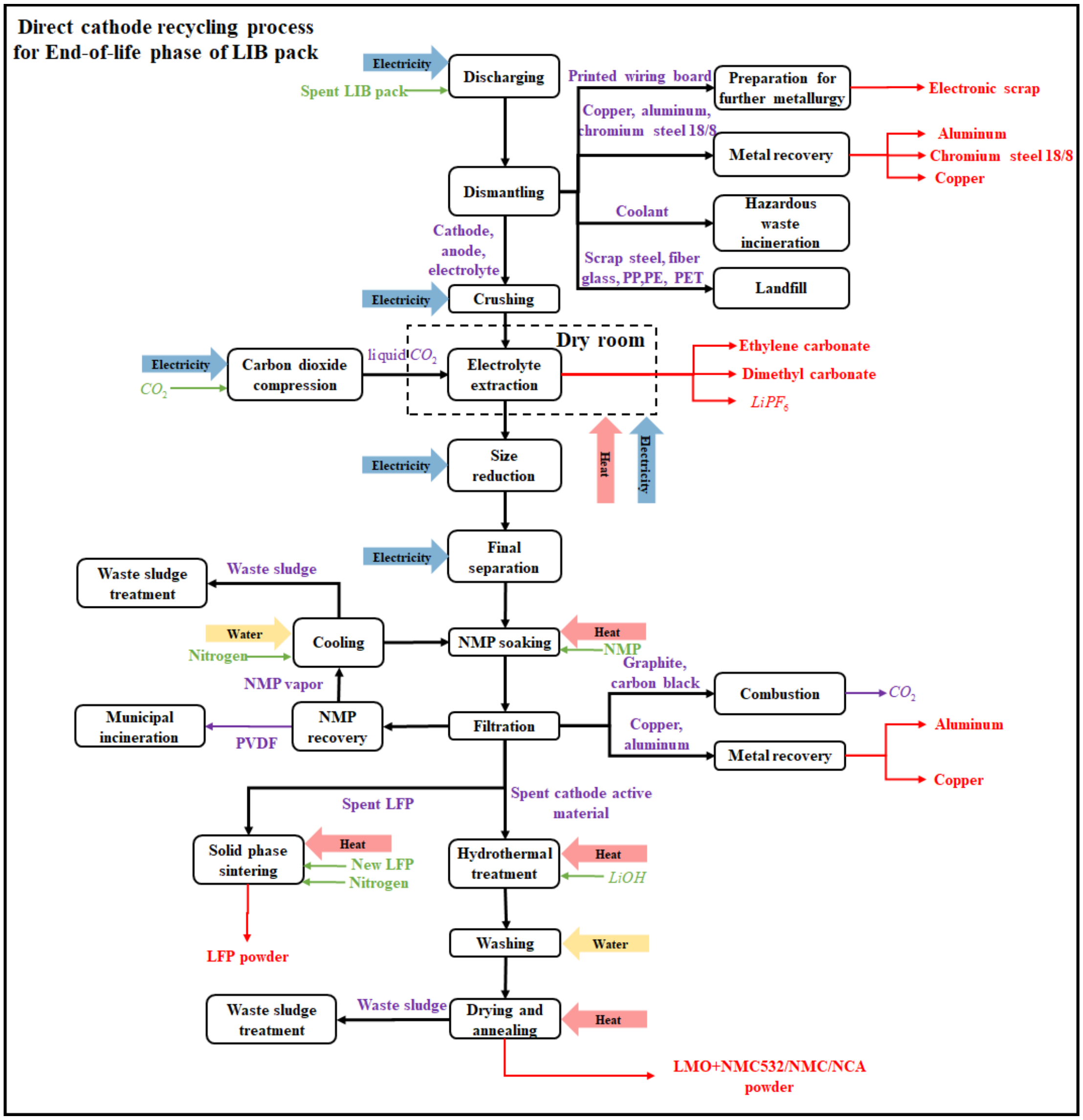


**Fig. S5. Process diagram of the direct cathode recycling.**

**Table S12. Material input and output of direct cathode recycling for EOL LIB packs with an initial energy storage capacity of 562.5 kWh (LFP, LMO/NMC532, NMC622, NCA).**

| | | After EV use | | | | After cascaded use | | | |
|---|---|---|---|---|---|---|---|---|---|
| | | LFP | LMO/ NMC | NMC622 | NCA | LFP | LMO/ NMC | NMC622 | NCA |
| Material input (kg) | EV LIB pack | 3218.89 | 2475.02 | 2220.15 | 2158.19 | - | - | - | - |
| | ESS LIB pack | - | - | - | - | 3087.93 | 2461.03 | 2170.89 | 2100.00 |
| | Waste aluminum | - | - | - | - | 126.39 | 48.95 | 54.03 | 55.36 |
| | Waste fibre glass | - | - | - | - | 186.42 | 112.69 | 124.37 | 127.43 |
| | Waste copper | - | - | - | - | 64.08 | 50.97 | 47.58 | 46.65 |
| | Waste printed wiring board | - | - | - | - | 9.66 | 7.43 | 6.66 | 6.47 |
| | Waste steel | - | - | - | - | 38.63 | 29.70 | 26.64 | 25.90 |
| | Waste coolant | - | - | - | - | 77.77 | 53.11 | 58.63 | 60.14 |
| | NMP (*93*) | 1834.54 | 1695.68 | 1417.13 | 1352.78 | 1834.54 | 1695.68 | 1417.13 | 1352.78 |
| | Decarbonised water (*21*) | 148165.86 | 136951.84 | 114454.37 | 109256.35 | 148165.86 | 136951.84 | 114454.37 | 109256.35 |
| | Nitrogen (*21*) | 31.01 | 28.66 | 23.95 | 22.86 | 31.01 | 28.66 | 23.95 | 22.86 |
| | Steam (*21*) | 2728.60 | 2522.08 | 2107.77 | 2012.05 | 2728.60 | 2522.08 | 2107.77 | 2012.05 |
| | LiOH (*40, 105*) | - | 29.08 | 22.06 | 20.23 | - | 58.15 | 44.12 | 40.46 |
| | LFP (*42*) | 498.52 | - | - | - | 498.52 | - | - | |
| | Deionised water (*40, 106*) | - | 5893.74 | 4470.92 | 4102.25 | - | 6195.70 | 4699.99 | 4314.27 |
| | $N_2$ (*42*) | 1306.13 | - | - | - | 1306.13 | - | - | - |
| | $CO_2$ (*41*) | 144086.72 | 87957.05 | 78334.82 | 75744.22 | 144086.72 | 87957.05 | 78334.82 | 75744.22 |
| | Acetonitrile (ACN) (*41*) | 857.97 | 523.74 | 466.45 | 451.02 | 857.97 | 523.74 | 466.45 | 451.02 |
| | Propylene carbonate (PC) (*41*) | 438.45 | 267.65 | 238.37 | 230.48 | 438.45 | 267.65 | 238.37 | 230.48 |
| Energy input (kg) | Electricity: Discharging (kWh) (*93*) | 47.37 | 34.88 | 34.38 | 31.70 | 47.37 | 34.88 | 34.38 | 31.70 |
| | Electricity: Liquid CO2 compression (kWh) (*107*) | 14120.50 | 8619.79 | 7676.81 | 7422.93 | 14120.50 | 8619.79 | 7676.81 | 7422.93 |
| | Electricity: Size reduction (kWh) (*93*) | 167.25 | 135.51 | 117.42 | 113.00 | 167.25 | 135.51 | 117.42 | 113.00 |
| | Electricity: Final separation (kWh) (*93*) | 14.42 | 11.68 | 10.12 | 9.74 | 14.42 | 11.68 | 10.12 | 9.74 |
| | Electricity: Dry room operation (kWh) (*93*) | 456.36 | 369.74 | 320.40 | 369.27 | 456.36 | 369.74 | 320.40 | 369.27 |
| | Heat: Dry room operation (MJ) (*93*) | 3011.99 | 2440.31 | 2114.60 | 2437.20 | 3011.99 | 2440.31 | 2114.60 | 2437.20 |
| | Heat: NMP soaking (MJ) (*93*) | 439.51 | 401.00 | 336.36 | 321.30 | 439.51 | 401.00 | 336.36 | 321.30 |
| | Heat: Hydrothermal process (MJ) (*40*) | - | 1778.19 | 1348.91 | 1003.68 | - | 1778.19 | 1348.91 | 1003.68 |

| | | | | | | | | | |
|---|---|---|---|---|---|---|---|---|---|
| | Heat: Annealing process (MJ) (*40*, *42*) | 9980.39 | 4797.75 | 3639.52 | 3337.82 | 9980.39 | 4797.75 | 3639.52 | 3337.82 |
| Output waste (kg) | Waste copper | 2.41 | 1.48 | 1.55 | 1.56 | 2.92 | 1.90 | 1.95 | 1.95 |
| | Waste aluminium | 16.96 | 7.71 | 8.82 | 9.05 | 16.96 | 7.71 | 8.82 | 9.05 |
| | Waste printed wiring board | 9.66 | 7.43 | 6.66 | 6.47 | 14.29 | 11.12 | 9.92 | 9.62 |
| | Waste chromium steel 18/8 | 3.09 | 2.38 | 2.13 | 2.07 | 4.57 | 3.56 | 3.17 | 3.08 |
| | Waste coolant | 77.77 | 53.11 | 58.63 | 60.14 | 155.54 | 106.22 | 117.25 | 120.28 |
| | Waste fibre glass | 186.42 | 112.69 | 124.37 | 127.43 | 186.42 | 112.69 | 124.37 | 127.43 |
| | Waste steel | 13.37 | 6.11 | 7.52 | 7.92 | 245.48 | 191.10 | 170.70 | 165.77 |
| | Waste PP | 20.88 | 8.47 | 9.71 | 9.78 | 20.88 | 8.47 | 9.71 | 9.78 |
| | Waste PE | 4.77 | 1.89 | 2.15 | 2.16 | 4.77 | 1.89 | 2.15 | 2.16 |
| | Waste PET | 4.16 | 2.17 | 2.57 | 2.67 | 4.16 | 2.17 | 2.57 | 2.67 |
| | Waste sludge | - | 6039.70 | 4585.59 | 4208.89 | - | 6370.74 | 4836.71 | 4441.13 |
| | Waste PVDF | 36.68 | 33.93 | 28.35 | 27.05 | 36.68 | 33.93 | 28.35 | 27.05 |
| Avoided material (kg) | Chromium steel 18/8(*102*) | 35.54 | 27.32 | 24.51 | 23.83 | 52.58 | 40.91 | 36.49 | 35.42 |
| | Aluminium (*103*) | 322.23 | 146.45 | 167.49 | 171.95 | 322.23 | 146.45 | 167.49 | 171.95 |
| | Copper (*104*) | 305.63 | 163.74 | 175.49 | 176.11 | 344.07 | 195.62 | 205.65 | 205.75 |
| | Cathode active material (*93*) | 1661.74 | 1062.44 | 805.95 | 739.14 | 1661.74 | 1062.44 | 805.95 | 739.14 |
| | $LiPF_6$ (*41*) | 96.13 | 96.13 | 96.13 | 96.13 | 96.13 | 96.13 | 96.13 | 96.13 |
| | EC (*41*) | 101.42 | 47.72 | 38.52 | 36.04 | 101.42 | 47.72 | 38.52 | 36.04 |
| | DMC (*41*) | 109.51 | 51.54 | 41.60 | 38.92 | 109.51 | 51.54 | 41.60 | 38.92 |
| | $CO_2$ (*41*) | 144086.72 | 87957.05 | 78334.82 | 75744.22 | 144086.72 | 87957.05 | 78334.82 | 75744.22 |
| | ACN (*41*) | 857.97 | 523.74 | 466.45 | 451.02 | 857.97 | 523.74 | 466.45 | 451.02 |
| | PC (*41*) | 438.45 | 267.65 | 238.37 | 230.48 | 438.45 | 267.65 | 238.37 | 230.48 |
| | NMP (*96*) | 1815.11 | 1677.73 | 1402.12 | 1338.44 | 1815.11 | 1677.73 | 1402.12 | 1338.44 |
| Emitted (kg) | $CO_2$ (*27*, *97*, *99*) | 2488.81 | 2165.35 | 2155.70 | 2008.15 | 2488.81 | 2165.35 | 2155.70 | 2008.15 |
| | $N_2$ | 1306.13 | - | - | - | 1306.13 | - | - | - |

**Table S13. Material input and output of direct cathode recycling for EOL LIB packs with an initial energy storage capacity of 562.5 kWh (NMC333, NMC532, NMC811).**

| | | After EV use | | | After cascaded use | | |
|---|---|---|---|---|---|---|---|
| | | NMC333 | NMC532 | NMC811 | NMC333 | NMC532 | NMC811 |
| Material input (kg) | EV LIB pack | 2431.72 | 2330.39 | 2145.40 | - | - | - |
| | ESS LIB pack | - | - | - | 2398.90 | 2297.31 | 2095.49 |
| | Waste aluminum | - | - | - | 53.35 | 51.58 | 52.84 |
| | Waste fibre glass | - | - | - | 122.82 | 118.73 | 121.64 |
| | Waste copper | - | - | - | 50.97 | 49.23 | 46.46 |
| | Waste printed wiring board | - | - | - | 7.30 | 6.99 | 6.44 |
| | Waste steel | - | - | - | 29.18 | 27.96 | 25.74 |
| | Waste coolant | - | - | - | 57.98 | 55.92 | 57.33 |
| | NMP (*93*) | 1604.77 | 1545.32 | 1352.91 | 1604.77 | 1545.32 | 1352.91 |
| | Decarbonised water (*21*) | 129600.44 | 124807.87 | 110188.79 | 129600.44 | 124807.87 | 110188.79 |
| | Nitrogen (*21*) | 27.12 | 26.12 | 23.06 | 27.12 | 26.12 | 23.06 |
| | Steam (*21*) | 2386.70 | 2298.44 | 2029.22 | 2386.70 | 2298.44 | 2029.22 |
| | LiOH (*40*, *105*) | 26.17 | 25.27 | 18.72 | 52.33 | 50.53 | 37.45 |
| | Deionised water (*40*, *106*) | 5303.68 | 5121.45 | 3795.09 | 5575.41 | 5383.84 | 3989.52 |
| | $CO_2$ (*41*) | 85243.09 | 80061.89 | 77594.65 | 85243.09 | 80061.89 | 77594.65 |
| | Acetonitrile (ACN) (*41*) | 507.58 | 476.73 | 462.04 | 507.58 | 476.73 | 462.04 |
| | Propylene carbonate (PC) (*41*) | 259.39 | 243.62 | 236.12 | 259.39 | 243.62 | 236.12 |
| Energy input (kg) | Electricity: Discharging (kWh) (*93*) | 40.98 | 39.53 | 28.98 | 40.98 | 39.53 | 28.98 |
| | Electricity: Liquid CO2 compression (kWh) (*107*) | 8353.82 | 7846.06 | 7604.28 | 8353.82 | 7846.06 | 7604.28 |
| | Electricity: Size reduction (kWh) (*93*) | 130.90 | 125.33 | 113.26 | 130.90 | 125.33 | 113.26 |
| | Electricity: Final separation (kWh) (*93*) | 11.28 | 10.80 | 9.76 | 11.28 | 10.80 | 9.76 |
| | Electricity: Dry room operation (kWh) (*93*) | 357.17 | 341.98 | 309.04 | 357.17 | 341.98 | 309.04 |
| | Heat: Dry room operation (MJ) (*93*) | 2357.35 | 2257.05 | 2039.65 | 2357.35 | 2257.05 | 2039.65 |
| | Heat: NMP soaking (MJ) (*93*) | 380.40 | 366.05 | 322.80 | 380.40 | 366.05 | 322.80 |
| | Heat: Hydrothermal process (MJ) (*40*) | 1600.16 | 1545.18 | 1145.01 | 1600.16 | 1545.18 | 1145.01 |
| | Heat: Annealing process (MJ) (*40*) | 4317.42 | 4169.08 | 3089.36 | 4317.42 | 4169.08 | 3089.36 |
| Output waste (kg) | Waste copper | 1.60 | 1.52 | 1.50 | 2.02 | 1.93 | 1.89 |
| | Waste aluminium | 8.73 | 8.25 | 8.47 | 8.73 | 8.25 | 8.47 |
| | Waste printed wiring board | 7.30 | 6.99 | 6.44 | 10.89 | 10.44 | 9.58 |
| | Waste chromium steel 18/8 | 2.33 | 2.24 | 2.06 | 3.49 | 3.34 | 3.07 |
| | Waste coolant | 57.98 | 55.92 | 57.33 | 115.95 | 111.84 | 114.66 |
| | Waste fibre glass | 122.82 | 118.73 | 121.64 | 122.82 | 118.73 | 121.64 |
| | Waste steel | 7.32 | 6.83 | 7.18 | 183.80 | 179.51 | 164.69 |
| | Waste PP | 9.95 | 10.97 | 10.80 | 9.95 | 10.97 | 10.80 |
| | Waste PE | 2.22 | 2.49 | 2.43 | 2.22 | 2.49 | 2.43 |
| | Waste PET | 2.50 | 2.35 | 2.48 | 2.50 | 2.35 | 2.48 |
| | Waste sludge | 5437.84 | 5249.01 | 3898.54 | 5735.74 | 5536.67 | 4111.70 |
| | Waste PVDF | 31.88 | 30.92 | 51.06 | 31.88 | 30.92 | 51.06 |
| Avoided material (kg) | Chromium steel 18/8 (*102*) | 26.85 | 25.73 | 23.69 | 40.09 | 38.41 | 35.25 |
| | Aluminium (*103*) | 165.96 | 156.80 | 160.94 | 165.96 | 156.80 | 160.94 |
| | Copper (*104*) | 180.83 | 169.23 | 167.08 | 212.89 | 200.33 | 196.68 |
| | Cathode active material (*93*) | 956.07 | 923.22 | 684.12 | 956.07 | 923.22 | 684.12 |
| | $LiPF_6$ (*41*) | 96.13 | 96.13 | 96.13 | 96.13 | 96.13 | 96.13 |
| | EC (*41*) | 45.13 | 40.17 | 37.81 | 45.13 | 40.17 | 37.81 |
| | DMC (*41*) | 48.73 | 43.38 | 40.83 | 48.73 | 43.38 | 40.83 |
| | $CO_2$ (*41*) | 85243.09 | 80061.89 | 77594.65 | 85243.09 | 80061.89 | 77594.65 |
| | ACN (*41*) | 507.58 | 476.73 | 462.04 | 507.58 | 476.73 | 462.04 |
| | PC (*41*) | 259.39 | 243.62 | 236.12 | 259.39 | 243.62 | 236.12 |
| | NMP (*96*) | 1587.67 | 1528.96 | 1349.87 | 1587.67 | 1528.96 | 1349.87 |

| | | | | | | | |
|---|---|---|---|---|---|---|---|
| Emitted (kg) | $CO_2$ (*97, 99*) | 2283.81 | 2189.05 | 2280.81 | 2283.81 | 2189.05 | 2280.81 |

Pyrometallurgical Recycling

Fig. S6 depicts the process design of pyrometallurgical recycling. Tables S14–S15 detail the bill of materials and energy. Pyrometallurgical recycling is developed by Umicore and is designed to recover Ni and Co by forming an alloy through a three-stage smelting. The spent LIB pack is disassembled to the module level and disposed of as described in that of the hydrometallurgical recycling. After disassembly, the left spent LIB modules are fed directly into the smelting furnace together with slag-forming materials such as limestone, sand, and slag and a commonly used reducing agent in metal smelting, namely, coke. The furnace can be divided into three zones: the preheating zone (under 300 °C), the plastics pyrolyzing zone (700 °C), and the metal melting and reducing zone (ranged from 1200 to 1450 °C). The preheating zone and plastics pyrolyzing zone is where the electrolyte is evaporated and where the plastics are melted. Heated air is injected into the last zone where blast furnace slag and alloy are subsequently formed (*108*).

Flue gas containing $CO_2$, carbon monoxide (CO), formaldehyde, ethylene, acetylene, soot, hydrogen fluoride (HF), and a negligible amount of silicon tetrafluoride, as well as hydrocarbons, is formed from the electrolyte evaporation (*109*). Moreover, blast furnace gas containing $CO_2$, CO, hydrogen, and nitrogen, is formed during alloy formation (*110*). The content of the gaseous phase is given in Tables S16–S17. The gaseous phase is post-combusted and complete combustion is assumed. During combustion, $CO_2$, and HF are formed, where $CO_2$ is emitted, and HF is assumed to be recovered by water. Plastics are assumed to be treated the same as if it is sent to the municipal incineration. The slag containing 100% of Li and Al, 7.2% of copper (Cu), and 35.5% of iron (Fe) from the input are assumed to be granulated blast furnace slag and can be used as a strength-enhancing compound for concrete. The alloy consists of 92.8% Cu, 64.5% Fe, 99% Ni, and 94% Co from the input and can be treated to recover metals (*108*). The alloy is first leached by 6 M HCl at 95 °C with an S:L ratio of 100 g/L for three hours. The leaching efficiencies are 62%, 96%, 99%, and 98% for Co, Ni, Fe, and Cu (*111*). By adding iron scraps to the leachate, the copper ions are replaced by iron and precipitated at 80 °C. Next, $Na_2CO_3$ and sodium chlorate ($NaClO_3$) are added to precipitate goethite according to Eq. (8).

$$6FeCl_2 + NaClO_3 + 3H_2O + 6Na_2CO_3 \rightleftharpoons 6FeOOH \downarrow + 13NaCl \tag{8}$$

After copper and goethite precipitation, NaOH is added to the solution to precipitate $Ni(OH)_2$. The avoided $Ni(OH)_2$ is assumed to be precipitated from the reaction of NaOH and $NiSO_4$ as shown in Eq.(9). The bill of materials for producing $Ni(OH)_2$ is given in Table S13. The by-product of sodium sulfate is excluded from the system boundary using the concept of the “recycled content” approach. The energy demand for this reaction is assumed to be negligible.

$$NiSO_{4\,(aq)} + 2NaOH_{(aq)} \rightarrow Ni(OH)_{2\,(s)} + Na_2SO_{4\,(aq)} \tag{9}$$

In the end, only Co ions are left in the solution. We assume that the precipitates are washed and recovered. In addition, the cobalt ion solution is also assumed to be recovered.

## Table S14. Material input and output of $Ni(OH)_2$ production (*29*).

| Input | | Amount |
|---|---|---|
| Material | $NiSO_4$ (kg) | 1.67 |
| | NaOH (kg) | 0.86 |
| Transport | Rail (t·km) | 1.50 |
| | Road (t·km) | 0.25 |
| Infrastructure | Chemical plant construction (unit) | 4.6E-10 |
| Output | $Ni(OH)_2$ (kg) | 1.00 |

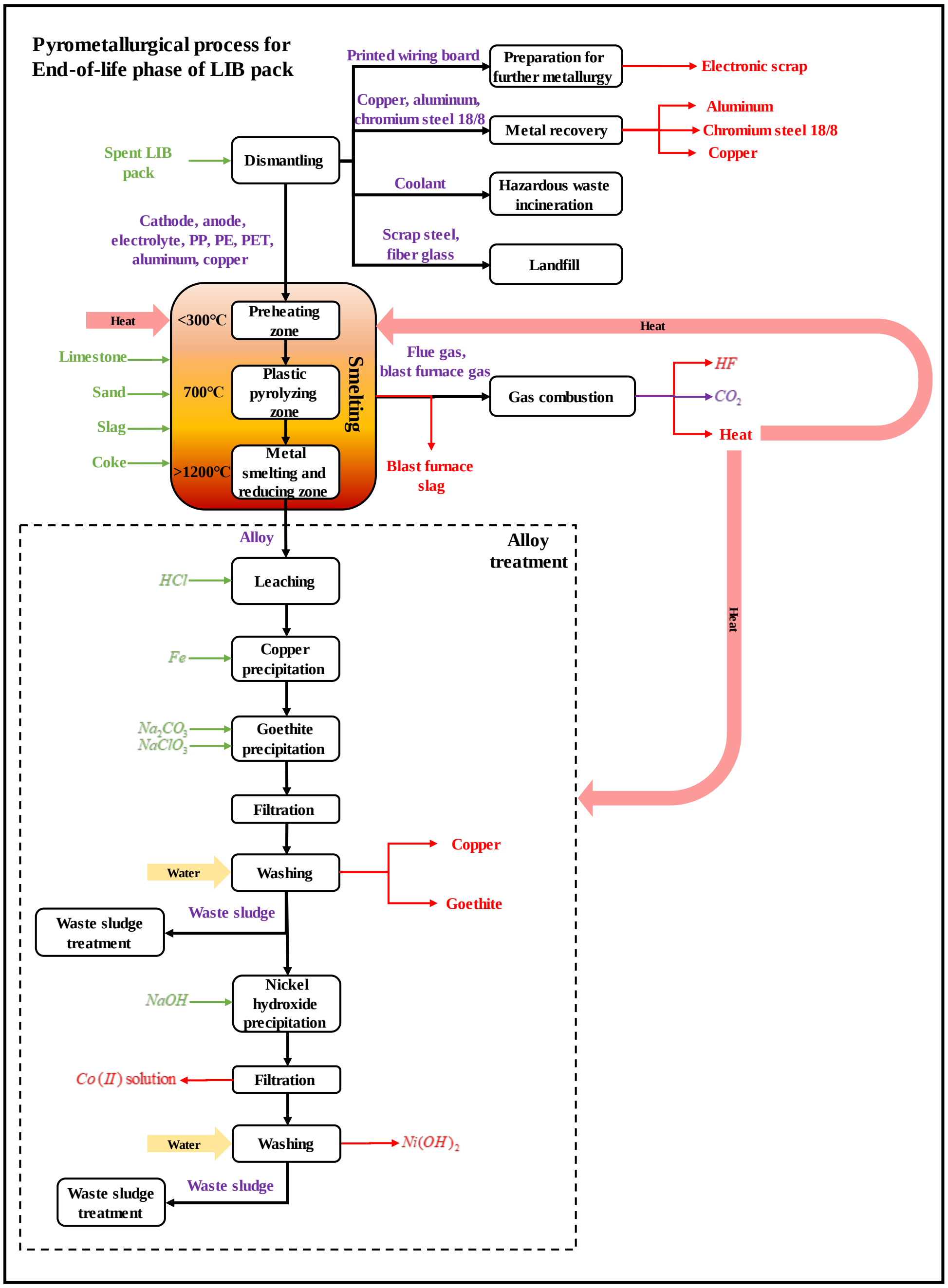


**Fig. S6. Process diagram of the pyrometallurgical recycling.**

**Table S15. Material input and output of pyrometallurgical recycling for EOL LIB packs with an initial energy storage capacity of 562.5 kWh (LMO/NMC532, NMC622, NCA).**

| | | After EV use | | | After cascaded use | | |
|---|---|---|---|---|---|---|---|
| | | LMO/NMC | NMC622 | NCA | LMO/NMC | NMC622 | NCA |
| Material input (kg) | EV LIB pack | 2475.02 | 2220.15 | 2158.19 | - | - | - |
| | ESS LIB pack | - | - | - | 2461.03 | 2170.89 | 2100.00 |
| | Waste aluminum | - | - | - | 48.95 | 54.03 | 55.36 |
| | Waste fibre glass | - | - | - | 112.69 | 124.37 | 127.43 |
| | Waste copper | - | - | - | 50.97 | 47.58 | 46.65 |
| | Waste printed wiring board | - | - | - | 7.43 | 6.66 | 6.47 |
| | Waste steel | - | - | - | 29.70 | 26.64 | 25.90 |
| | Waste coolant | - | - | - | 53.11 | 58.63 | 60.14 |
| | Limestone (*31*, *108*) | 180.51 | 157.89 | 152.36 | 195.92 | 171.49 | 165.51 |
| | Sand (*31*, *108*) | 198.56 | 173.68 | 167.60 | 215.51 | 188.64 | 182.06 |
| | Slag (*31*, *108*) | 361.01 | 315.79 | 304.72 | 391.84 | 342.98 | 331.03 |
| | Coke (*31*, *108*) | 722.02 | 631.58 | 609.44 | 783.68 | 685.97 | 662.05 |
| | HCl (*39*, *111*) | 817.82 | 1148.43 | 1890.20 | 818.26 | 1148.81 | 1890.57 |
| | Iron (*111*) | 93.50 | 105.95 | 107.23 | 93.50 | 105.95 | 107.23 |
| | $NaClO_3$ (*111*) | 30.36 | 34.21 | 34.59 | 30.42 | 34.27 | 34.64 |
| | $Na_2CO_3$ (*111*) | 181.39 | 204.41 | 206.69 | 181.77 | 204.74 | 207.01 |
| | $H_2SO_4$ (*97*) | 643.92 | 976.94 | - | 643.92 | 976.94 | - |
| | $H_2O_2$ (*97*) | 96.22 | 145.98 | - | 96.22 | 145.98 | - |
| | NaOH (*29*) | 261.58 | 396.87 | 489.83 | 261.58 | 396.87 | 489.83 |
| | Deionised water (*99*) | 13186.72 | 9057.55 | 10405.03 | 13194.10 | 9060.89 | 10408.26 |
| Energy input | Heat: Smelting (MJ) (*31*) | 1447.37 | 1266.07 | 1221.69 | 1570.98 | 1375.10 | 1327.16 |
| | Heat: Leaching (MJ) (*93*) | 47.32 | 66.45 | 71.89 | 47.35 | 66.48 | 71.91 |
| Output waste (kg) | Waste copper | 0.66 | 0.62 | 0.61 | 1.08 | 1.02 | 1.00 |
| | Waste aluminium | 2.45 | 2.70 | 2.77 | 2.45 | 2.70 | 2.77 |
| | Waste printed wiring board | 7.43 | 6.66 | 6.47 | 14.29 | 10.44 | 9.58 |
| | Waste chromium steel 18/8 | 2.38 | 2.13 | 2.07 | 4.57 | 3.34 | 3.07 |
| | Waste coolant | 53.11 | 58.63 | 60.14 | 155.54 | 111.84 | 114.66 |
| | Waste fibre glass | 112.69 | 124.37 | 127.43 | 186.42 | 118.73 | 121.64 |
| | Waste steel | 6.11 | 7.52 | 7.92 | 245.48 | 179.51 | 164.69 |
| | Waste sludge | 15021.97 | 11687.50 | 12752.65 | 14845.12 | 11298.66 | 12598.67 |
| | Incinerated PVDF | 33.93 | 28.35 | 27.05 | 36.68 | 30.92 | 51.06 |
| | Evaporated NMP | 16.29 | 13.61 | 12.98 | 16.29 | 13.61 | 12.98 |
| | Incinerated PP | 8.47 | 9.71 | 9.78 | 20.88 | 10.97 | 10.80 |
| | Incinerated PE | 1.89 | 2.15 | 2.16 | 4.77 | 2.49 | 2.43 |
| | Incinerated PET | 2.17 | 2.57 | 2.67 | 4.16 | 2.35 | 2.48 |
| Avoided material (kg) | Chromium steel 18/8 (*102*) | 27.32 | 24.51 | 23.83 | 40.91 | 36.49 | 35.42 |
| | Aluminium (*103*) | 46.50 | 51.33 | 52.59 | 46.50 | 51.33 | 52.59 |
| | Copper (*104*, *108*, *111*) | 153.98 | 164.44 | 164.93 | 185.86 | 194.60 | 194.57 |
| | Slag (*108*) | 1704.10 | 1222.61 | 1091.55 | 1952.10 | 1441.36 | 1303.16 |
| | Hydrogen fluoride (HF) (*109*) | 44.38 | 44.38 | 44.38 | 44.38 | 44.38 | 44.38 |
| | $Ni(OH)_2$ (*108*, *111*) | 304.17 | 461.47 | 569.57 | 304.17 | 461.47 | 569.57 |
| | $CoSO_4$ (*108*, *111*) | 104.72 | 158.88 | - | 104.72 | 158.88 | - |
| | $CoCl_2$ (*108*, *111*) | - | - | 88.51 | - | - | 88.51 |
| | Goethite (*111*) | 150.76 | 169.89 | 171.79 | 151.07 | | 172.05 |
| Energy output | Avoided heat (MJ) | 1494.70 | 1332.52 | 1293.58 | 1618.33 | 1441.58 | 1399.08 |
| | Waste heat (MJ) | 7329.80 | 6381.33 | 6090.28 | 7953.20 | 6931.24 | 6680.01 |
| Emitted | Heat (MJ) | 1417.39 | 1213.05 | 1143.09 | 1540.27 | 1321.45 | 1267.02 |
| | $CO_2$ (kg) (*109*, *110*) | 4481.63 | 4146.30 | 4092.68 | 4678.33 | 4319.81 | 4260.52 |
| | $N_2$ (kg) | 2012.64 | 1760.52 | 1698.81 | 2184.52 | 1912.14 | 1845.48 |

**Table S16. Material input and output of pyrometallurgical recycling for EOL LIB packs with an initial energy storage capacity of 562.5 kWh (NMC333, NMC532, NMC811).**

| | | After EV use | | | After cascaded use | | |
|---|---|---|---|---|---|---|---|
| | | NMC333 | NMC532 | NMC811 | NMC333 | NMC532 | NMC811 |
| Material input (kg) | EV LIB pack | 2431.72 | 2330.39 | 2145.40 | - | - | - |
| | ESS LIB pack | - | - | - | 2398.90 | 2297.31 | 2095.49 |
| | Waste aluminum | - | - | - | 53.35 | 51.58 | 52.84 |
| | Waste fibre glass | - | - | - | 122.82 | 118.73 | 121.64 |
| | Waste copper | - | - | - | 50.97 | 49.23 | 46.46 |
| | Waste printed wiring board | - | - | - | 7.30 | 6.99 | 6.44 |
| | Waste steel | - | - | - | 29.18 | 27.96 | 25.74 |
| | Waste coolant | - | - | - | 57.98 | 55.92 | 57.33 |
| | Limestone (*31*, *108*) | 175.23 | 167.76 | 152.31 | 190.26 | 182.15 | 165.44 |
| | Sand (*31*, *108*) | 192.76 | 184.54 | 167.54 | 209.29 | 200.37 | 181.98 |
| | Slag (*31*, *108*) | 350.47 | 335.52 | 304.63 | 380.52 | 364.30 | 330.88 |
| | Coke (*31*, *108*) | 700.93 | 671.05 | 609.25 | 761.04 | 728.61 | 661.76 |
| | HCl (*111*) | 1317.09 | 1260.78 | 1000.17 | 1317.51 | 1261.18 | 1000.54 |
| | Iron (*111*) | 107.59 | 99.45 | 99.94 | 107.59 | 99.45 | 99.94 |
| | $NaClO_3$ (*111*) | 34.80 | 32.19 | 32.28 | 34.86 | 32.25 | 32.34 |
| | $Na_2CO_3$ (*111*) | 207.92 | 192.34 | 192.89 | 208.29 | 192.69 | 193.21 |
| | $H_2SO_4$ (*97*) | 1158.91 | 1119.09 | 829.27 | 1158.91 | 1119.09 | 829.27 |
| | $H_2O_2$ (*97*) | 173.17 | 167.22 | 123.91 | 173.17 | 167.22 | 123.91 |
| | NaOH (*29*) | 470.79 | 454.61 | 336.88 | 470.79 | 454.61 | 336.88 |
| | Deionised water (*99*) | 10154.46 | 9661.18 | 8017.42 | 10158.15 | 9664.71 | 8020.64 |
| Energy input | Heat: Smelting (MJ) (*31*) | 1405.10 | 1345.19 | 1221.32 | 1525.59 | 1460.58 | 1326.57 |
| | Heat: Leaching (MJ) (*93*) | 76.21 | 72.95 | 57.87 | 76.24 | 72.98 | 57.90 |
| Output waste (kg) | Waste copper | 0.66 | 0.64 | 0.60 | 1.08 | 1.05 | 0.99 |
| | Waste aluminium | 2.67 | 2.58 | 2.64 | 2.67 | 2.58 | 2.64 |
| | Waste printed wiring board | 7.30 | 6.99 | 6.44 | 11.12 | 10.89 | 9.92 |
| | Waste chromium steel 18/8 | 2.33 | 2.24 | 2.06 | 3.56 | 3.49 | 3.17 |
| | Waste coolant | 57.98 | 55.92 | 57.33 | 106.22 | 115.95 | 117.25 |
| | Waste fibre glass | 122.82 | 118.73 | 121.64 | 112.69 | 122.82 | 124.37 |
| | Waste steel | 7.32 | 6.83 | 7.18 | 191.10 | 183.80 | 170.70 |
| | Waste sludge | 13198.68 | 12582.33 | 10292.16 | 12739.69 | 12137.92 | 9949.55 |
| | Incinerated PVDF | 31.88 | 30.92 | 51.06 | 33.93 | 31.88 | 28.35 |
| | Evaporated NMP | 15.30 | 14.84 | 24.51 | 15.30 | 14.84 | 24.51 |
| | Incinerated PP | 9.95 | 10.97 | 10.80 | 8.47 | 9.95 | 9.71 |
| | Incinerated PE | 2.22 | 2.49 | 2.43 | 1.89 | 2.22 | 2.15 |
| | Incinerated PET | 2.50 | 2.35 | 2.48 | 2.17 | 2.50 | 2.57 |
| Avoided material (kg) | Chromium steel 18/8 (*102*) | 26.85 | 25.73 | 23.69 | 40.09 | 38.41 | 35.25 |
| | Aluminium (*103*) | 50.68 | 49.00 | 50.20 | 50.68 | 49.00 | 50.20 |
| | Copper (*104, 108, 111*) | 169.59 | 158.86 | 156.66 | 201.66 | 189.96 | 186.27 |
| | Slag (*108*) | 1375.69 | 1318.40 | 1126.19 | 1617.42 | 1549.90 | 1337.34 |
| | Hydrogen fluoride (HF) (*109*) | 44.38 | 44.38 | 44.38 | 44.38 | 44.38 | 44.38 |
| | $Ni(OH)_2$ (*108*, *111*) | 547.43 | 528.62 | 391.72 | 547.43 | 528.62 | 391.72 |
| | $CoSO_4$ (*108*, *111*) | 188.48 | 182.00 | 134.87 | 188.48 | 182.00 | 134.87 |
| | Goethite (*111*) | 172.81 | 159.86 | 160.32 | 173.12 | 160.15 | 160.59 |
| Energy output | Avoided heat (MJ) | 1481.31 | 1418.14 | 1279.19 | 1601.83 | 1533.55 | 1384.46 |
| | Waste heat (MJ) | 7083.51 | 6776.57 | 6163.09 | 7691.17 | 7358.50 | 6693.89 |
| Emitted | Heat (MJ) | 1345.08 | 1286.11 | 1176.76 | 1464.86 | 1400.82 | 1281.39 |
| | $CO_2$ (kg) (*109*, *110*) | 4488.96 | 4286.64 | 4223.78 | 4680.70 | 4470.26 | 4391.26 |
| | $N_2$ (kg) | 1953.86 | 1870.55 | 1698.30 | 2121.40 | 2031.00 | 1844.65 |

## Table S17. Composition of gaseous phase from electrolyte combustion (*109*).

| | Gas Component | Amount (kg) |
|---|---|---|
| Input | Electrolyte | 1 |
| Output | $CO_2$ | 1.11 |
| | CO | 4.90E-03 |
| | Formaldehyde | 3.56E-04 |
| | Ethene | 8.91E-05 |
| | Ethyne | 7.13E-04 |
| | Total hydrocarbon | 6.06E-03 |
| | Soot | 0.012 |
| | HF | 0.099 |

## Table S18. Composition of blast furnace gas from Coking (*21*, *110*).

| Gas Component | Percentage (w %) |
|---|---|
| $CO_2$ | 28.93 |
| CO | 19.33 |
| $H_2$ | 0.2 |
| $N_2$ | 51.54 |

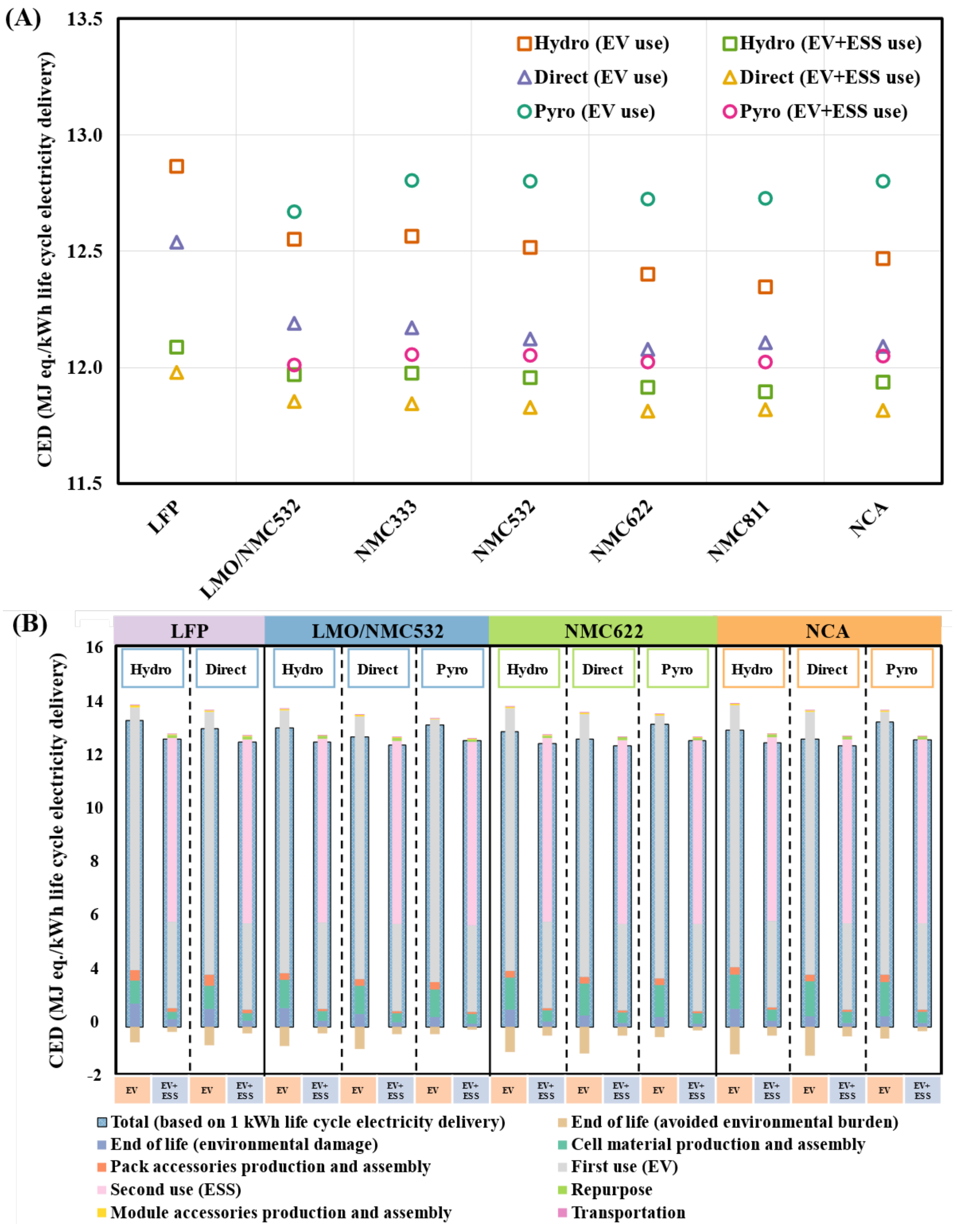


**Fig. S7. Breakdowns of CED for LFP, LMO/NMC532, NMC622, and NCA LIBs, with the hydrometallurgical, direct cathode, and pyrometallurgical recycling abbreviated as hydro, direct, and pyro.**

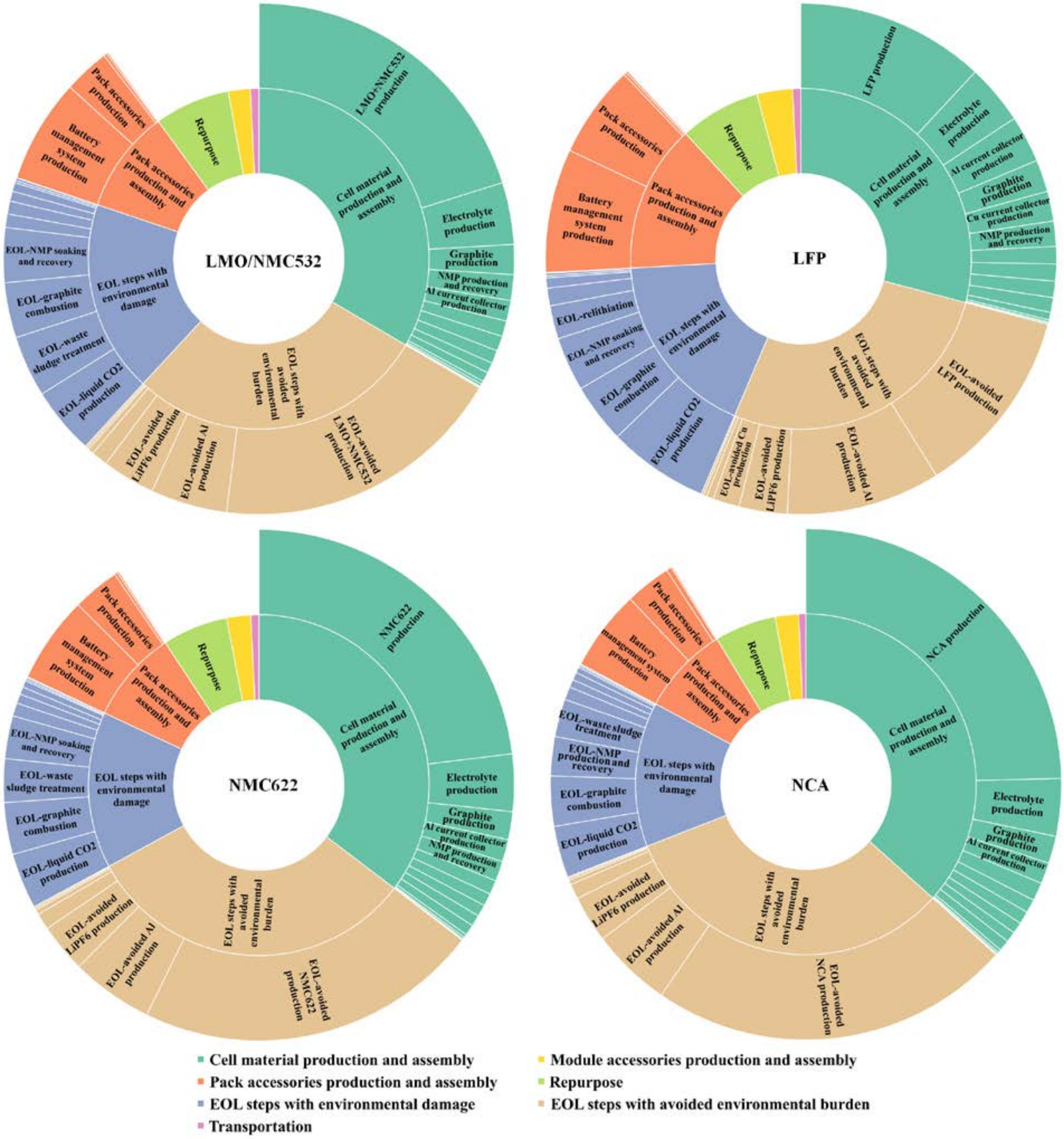


**Fig. S8. Carbon footprint hotspots of LFP, LMO/NMC532, NMC622, and NCA LIBs with direct cathode recycling.**

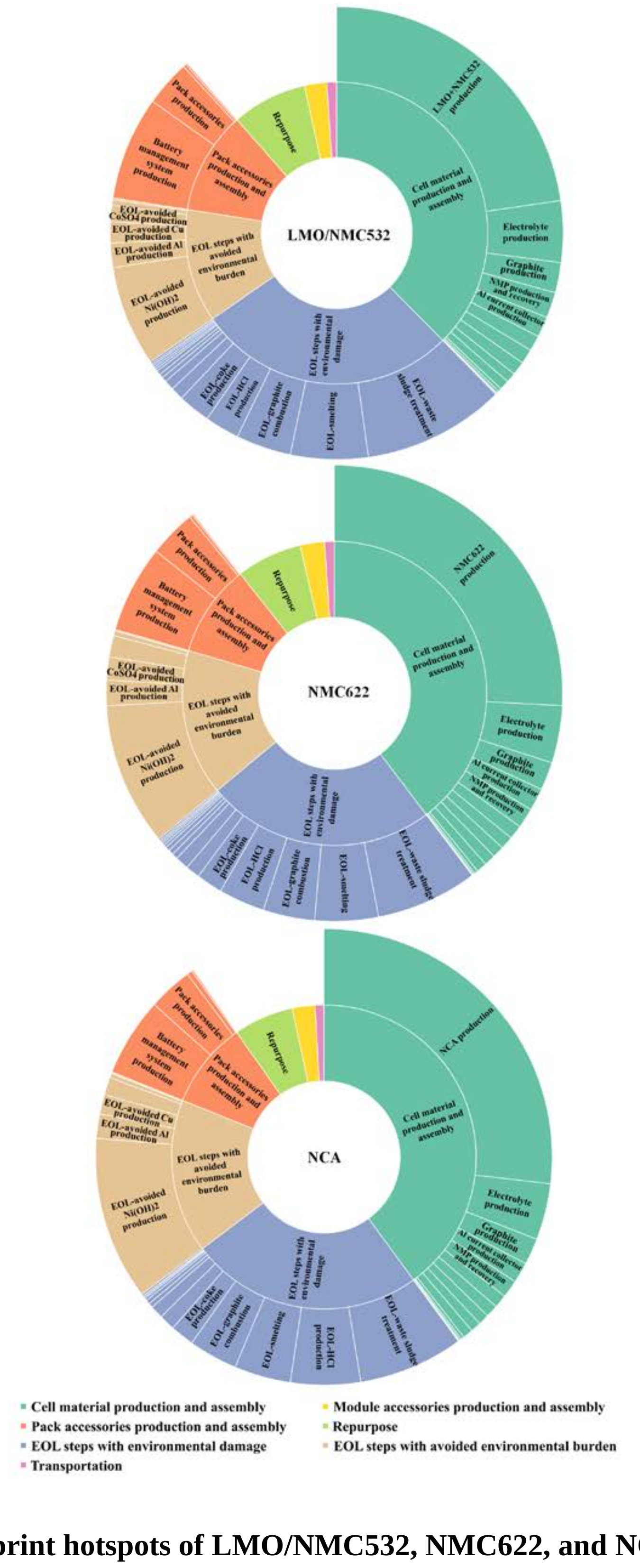


**Fig. S9. Carbon footprint hotspots of LMO/NMC532, NMC622, and NCA LIBs with pyrometallurgical recycling.**

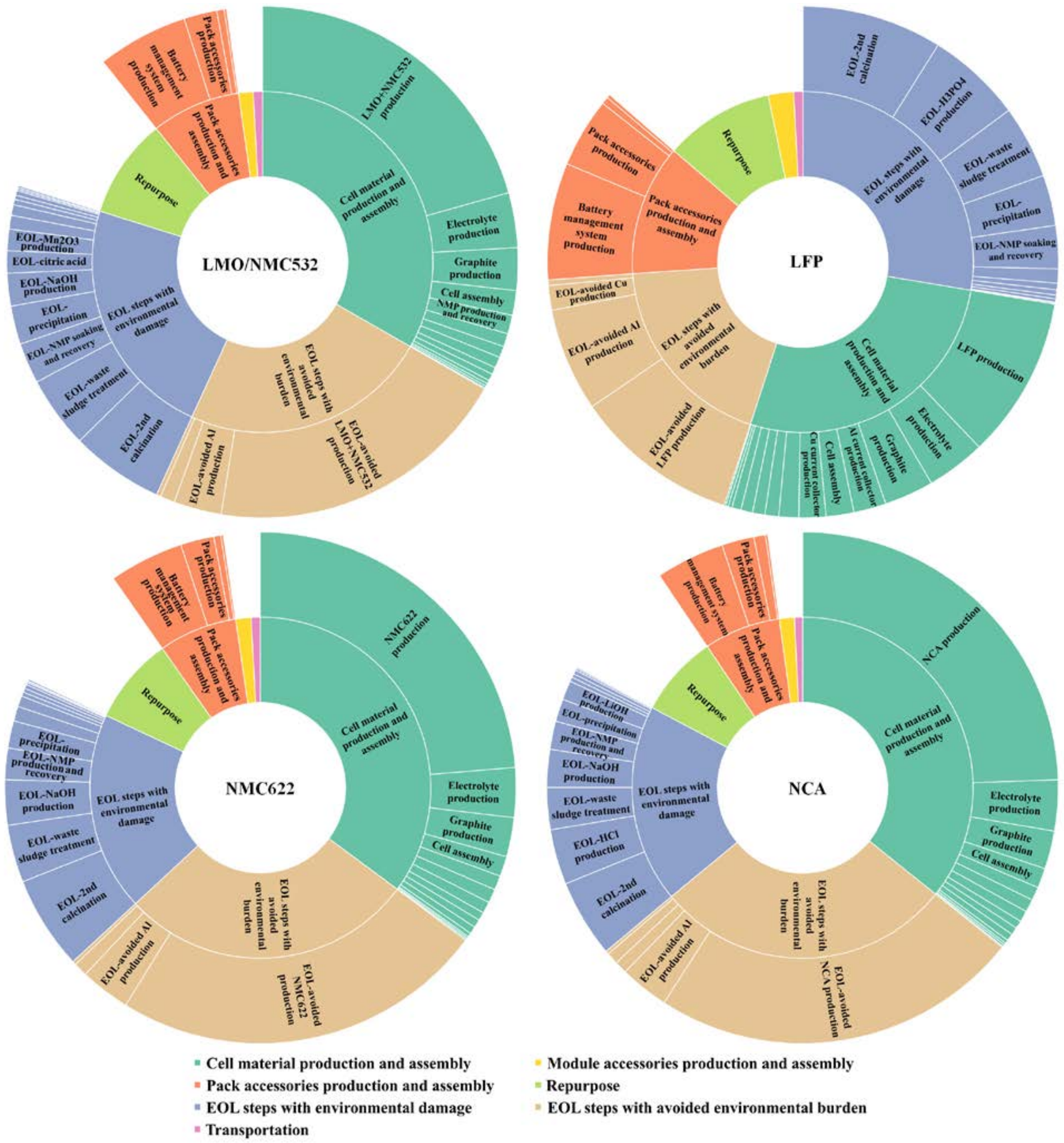


**Fig. S10. Energy hotspots of LFP, LMO/NMC532, NMC622, and NCA LIBs with hydrometallurgical recycling.**

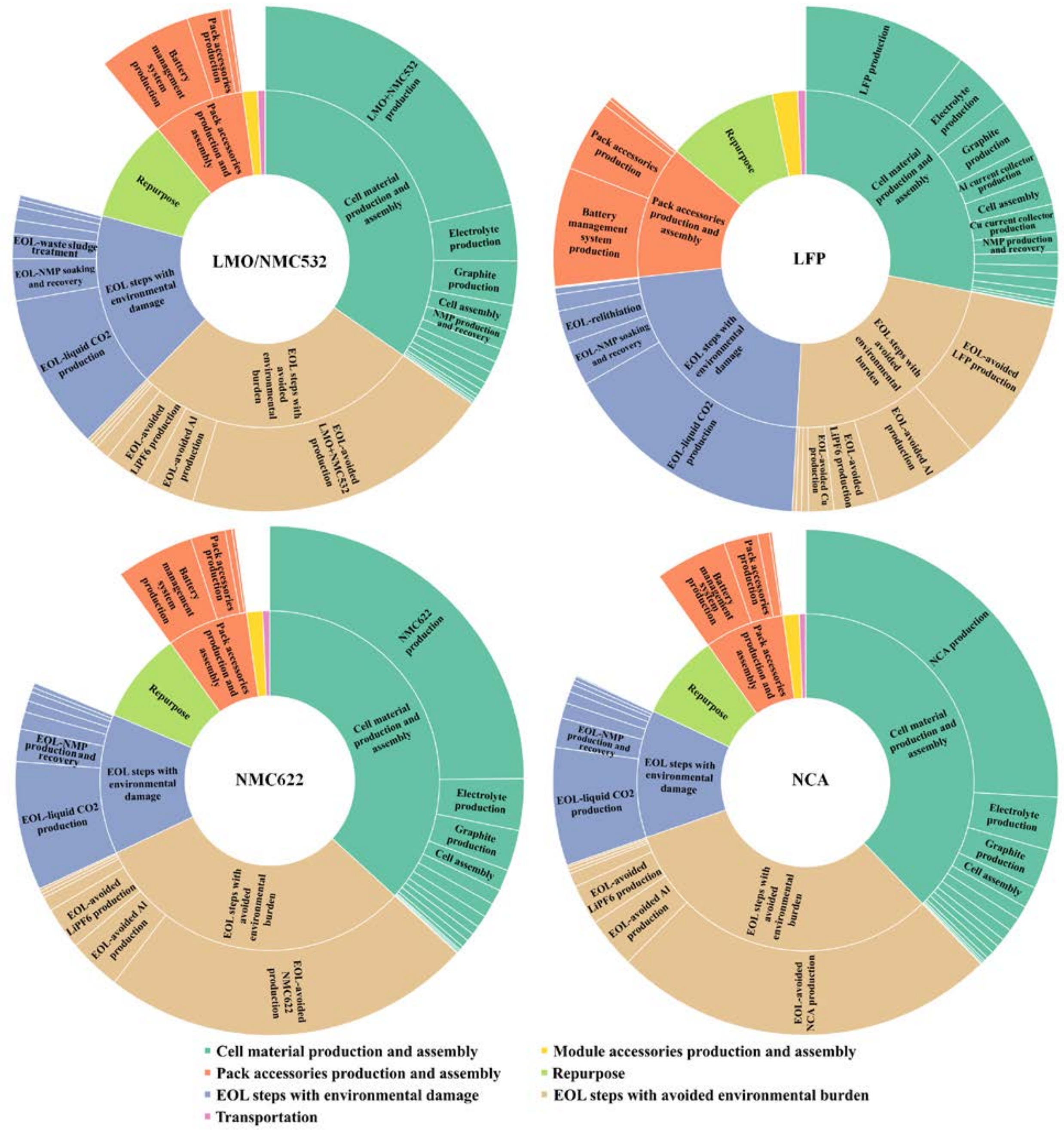


**Fig. S11. Energy hotspots of LFP, LMO/NMC532, NMC622, and NCA LIBs with direct cathode recycling.**

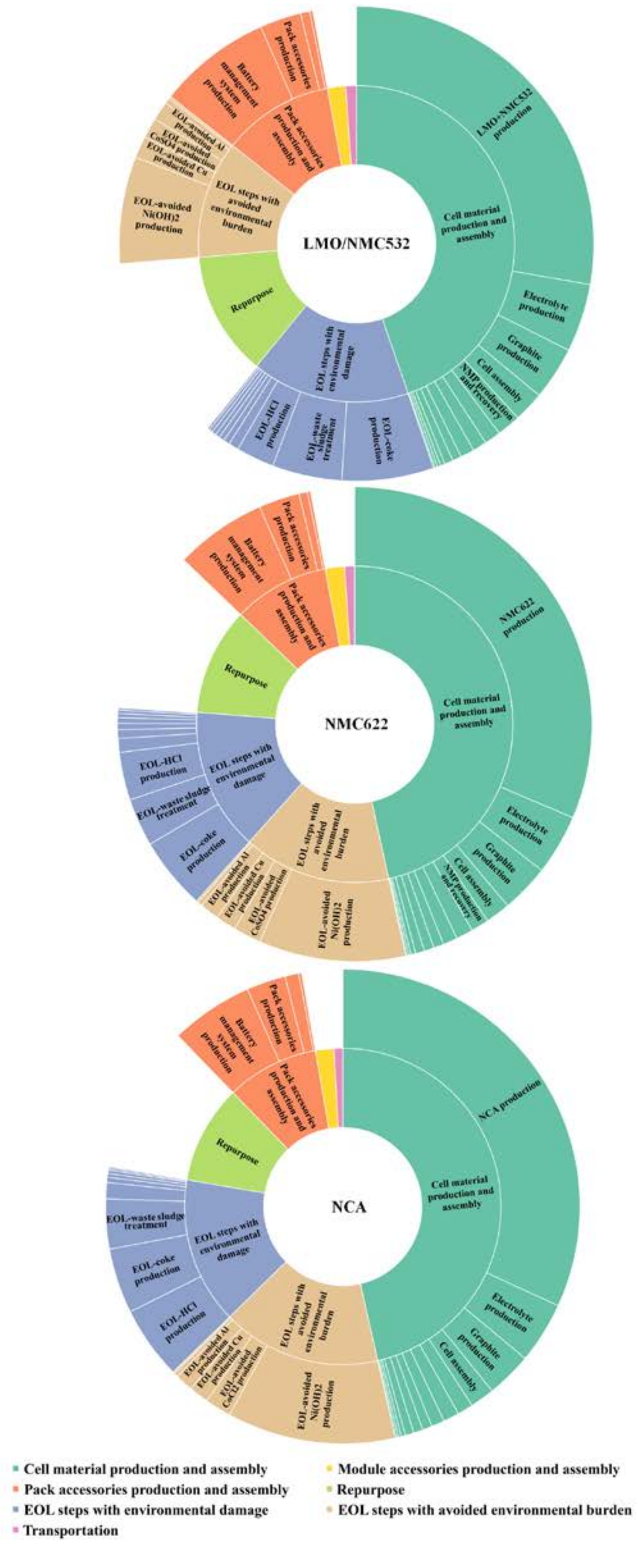


**Fig. S12. Energy hotspots of LMO/NMC532, NMC622, and NCA LIBs with pyrometallurgical recycling.**

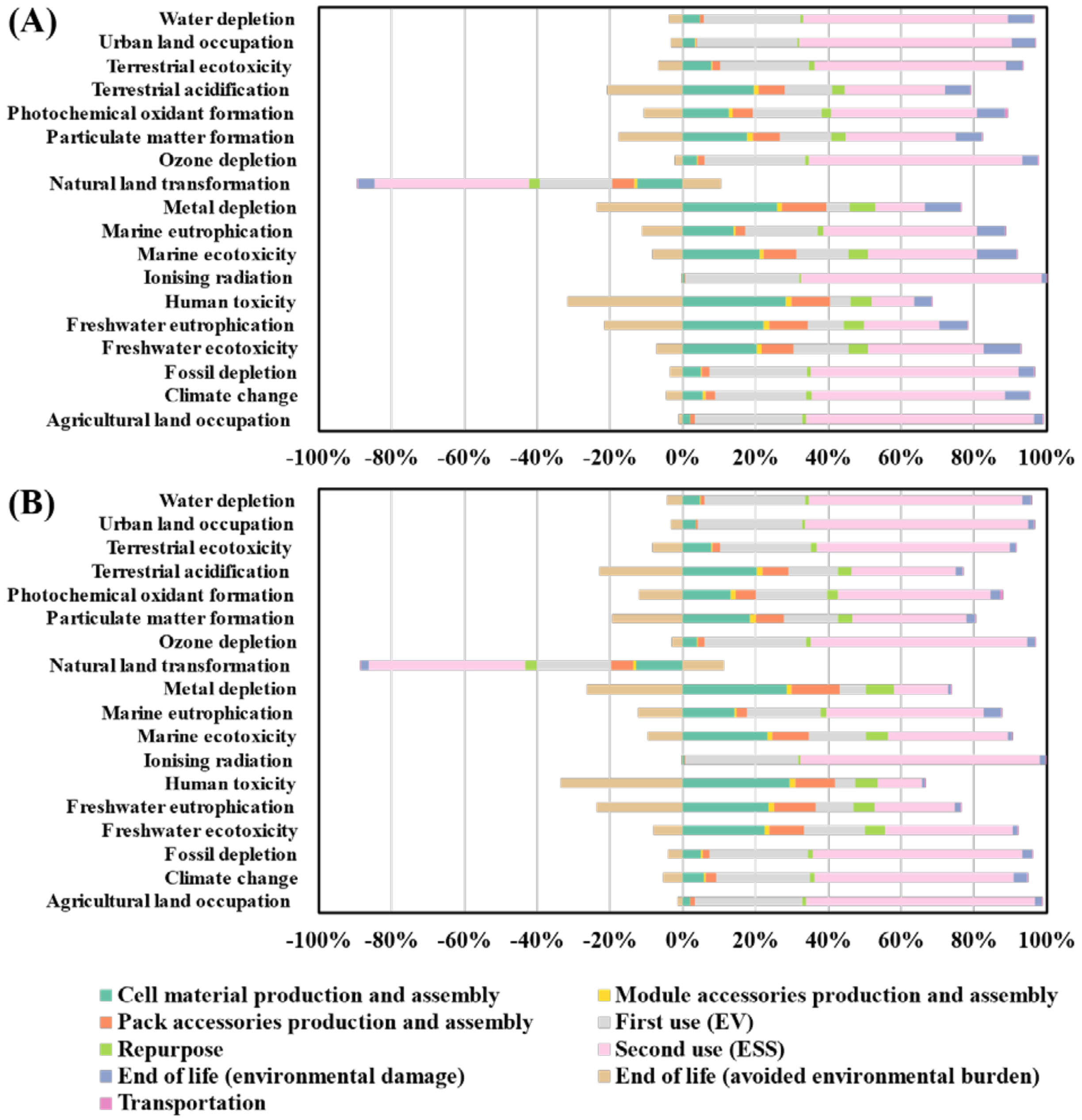


**Fig. S13. Comparison of full-spectrum environmental profiles for LFP LIBs across different recycling methods.** Full-spectrum environmental profiles for LMO/NMC532 LIBs subjected to second life and recycled by (**A**) hydrometallurgical recycling (**B**) direct cathode recycling on a percentage scale. Different colors in the stacked bars indicate different life cycle stages of LMO/NMC532 LIBs.

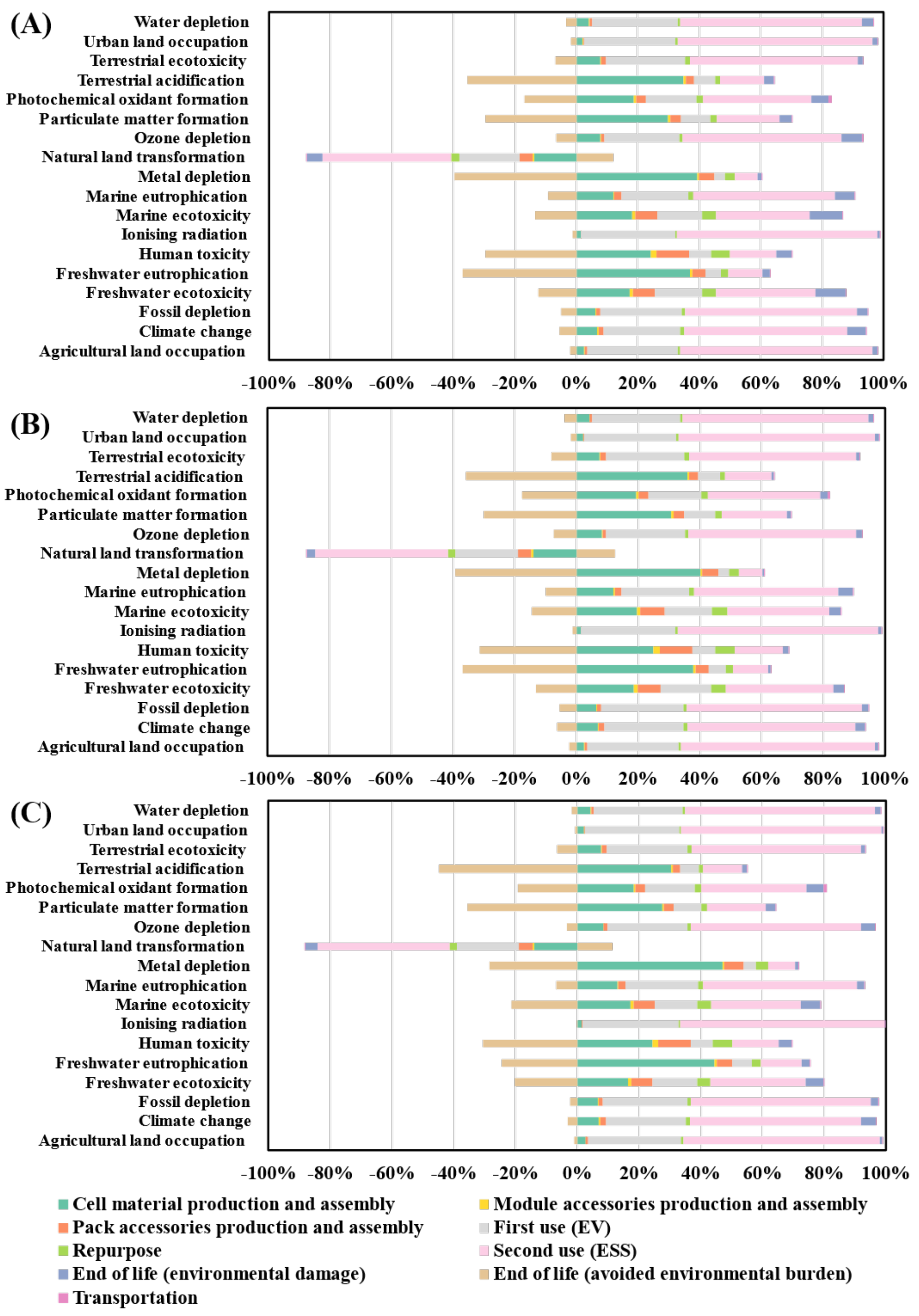


**Fig. S14. Comparison of full-spectrum environmental profiles for NMC333 LIBs across different recycling methods.** Full-spectrum environmental profiles for NMC333 LIBs subjected to second life and recycled by (**A**) hydrometallurgical recycling (**B**) direct cathode recycling (**C**) pyrometallurgical recycling on a percentage scale. Different colors in the stacked bars indicate different life cycle stages of NMC333 LIBs.

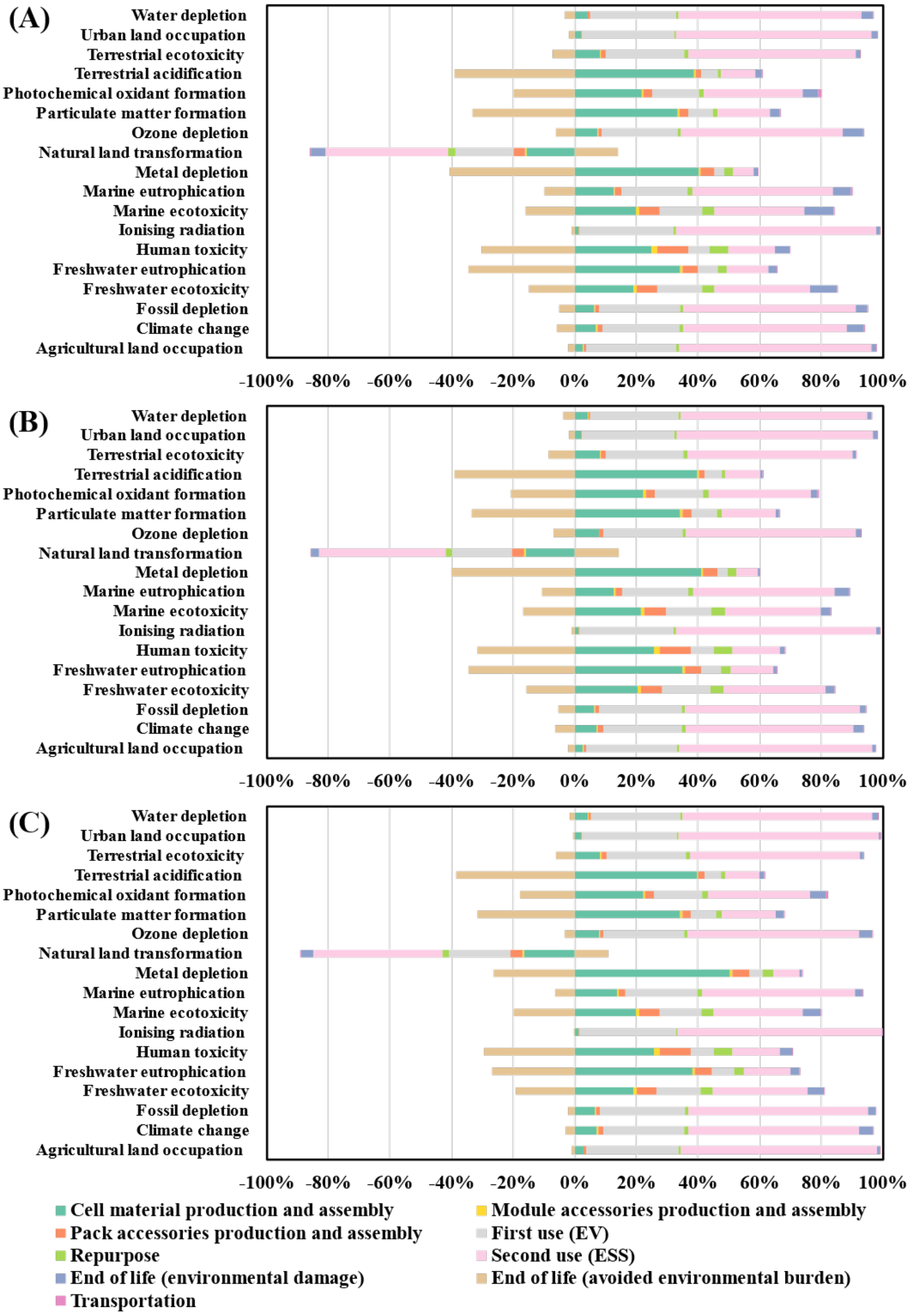


**Fig. S15. Comparison of full-spectrum environmental profiles for NMC532 LIBs across different recycling methods.** Full-spectrum environmental profiles for NMC532 LIBs subjected to second life and recycled by (**A**) hydrometallurgical recycling (**B**) direct cathode recycling (**C**) pyrometallurgical recycling on a percentage scale. Different colors in the stacked bars indicate different life cycle stages of NMC532 LIBs.

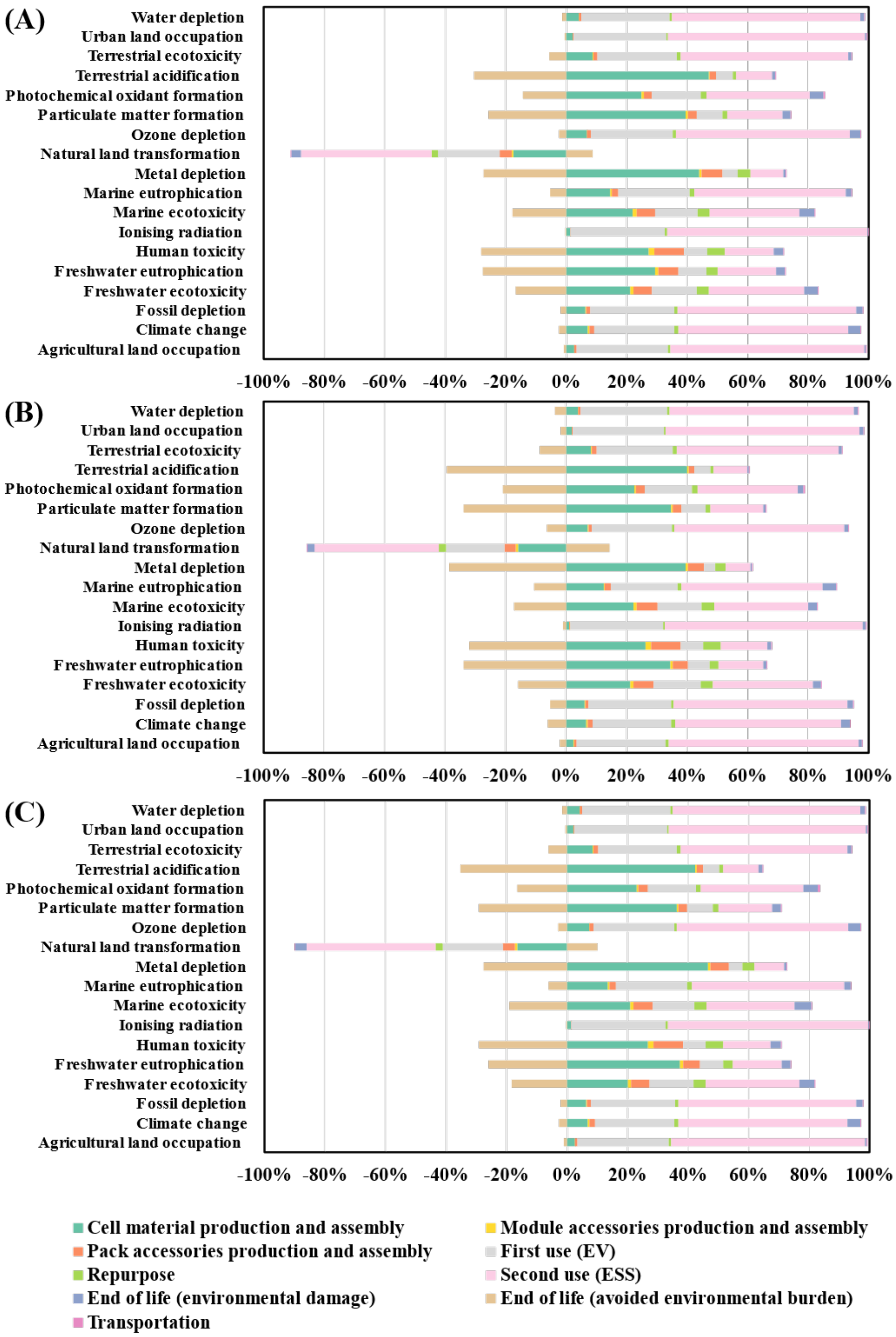


**Fig. S16. Comparison of full-spectrum environmental profiles for NMC622 LIBs across different recycling methods.** Full-spectrum environmental profiles for NMC622 LIBs subjected to second life and recycled by (**A**) hydrometallurgical recycling (**B**) direct cathode recycling (**C**) pyrometallurgical recycling on a percentage scale. Different colors in the stacked bars indicate different life cycle stages of NMC622 LIBs.

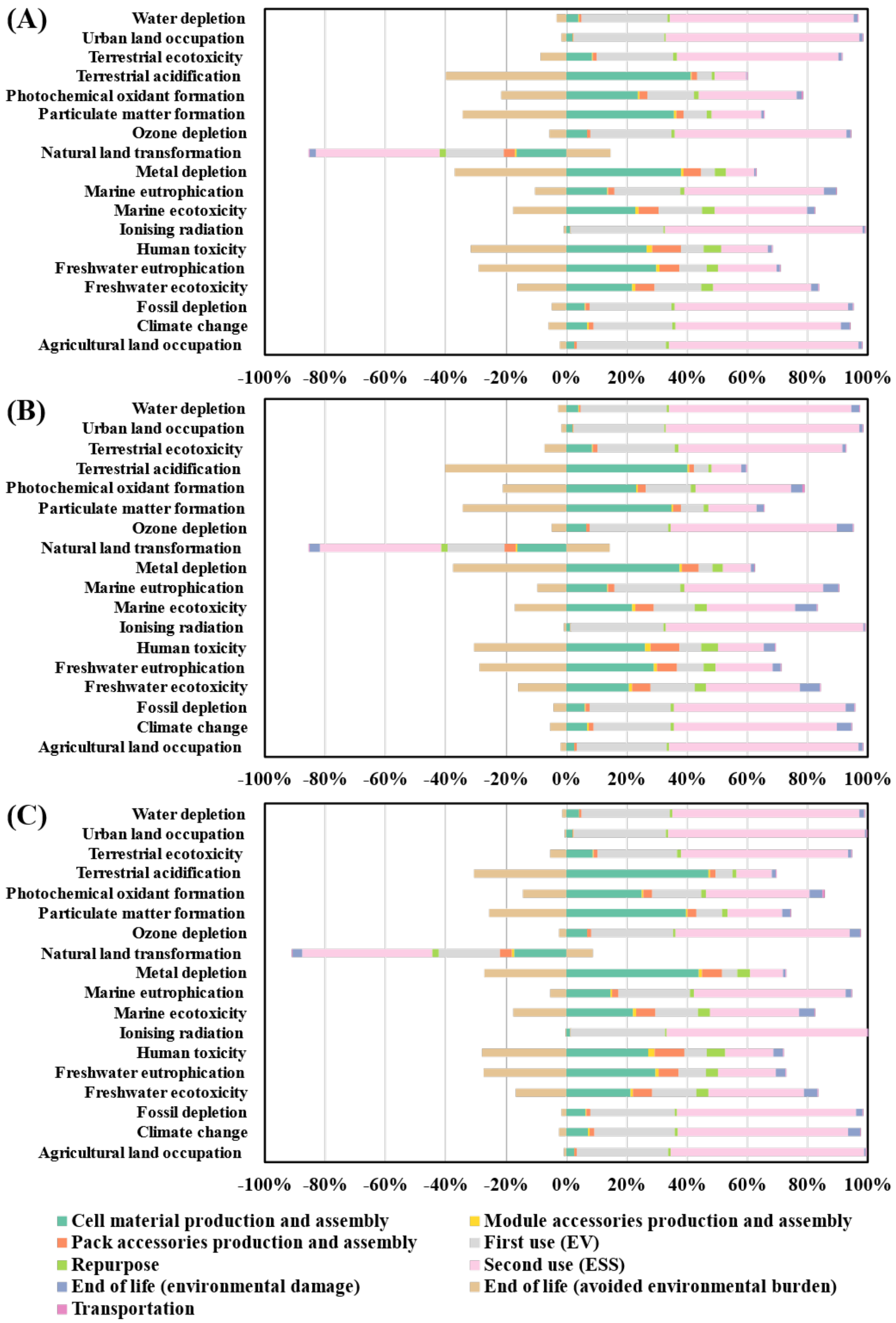


**Fig. S17. Comparison of full-spectrum environmental profiles for NMC811 LIBs across different recycling methods.** Full-spectrum environmental profiles for NMC811 LIBs subjected to second life and recycled by (**A**) hydrometallurgical recycling (**B**) direct cathode recycling (**C**) pyrometallurgical recycling on a percentage scale. Different colors in the stacked bars indicate different life cycle stages of NMC811 LIBs.

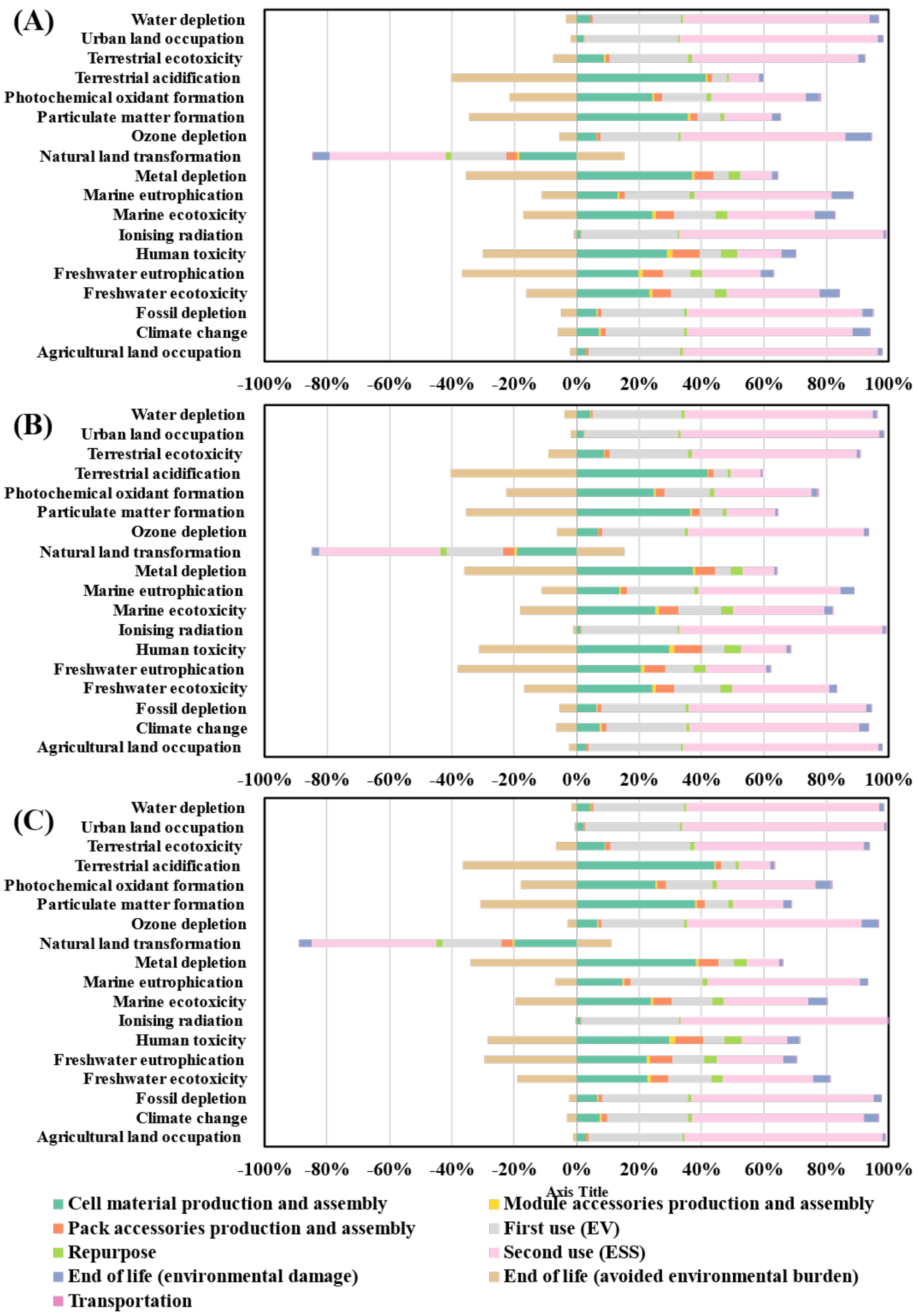


**Fig. S18. Comparison of full-spectrum environmental profiles for NCA LIBs across different recycling methods.** Full-spectrum environmental profiles for NCA LIBs subjected to second life and recycled by (**A**) hydrometallurgical recycling (**B**) direct cathode recycling (**C**) pyrometallurgical recycling on a percentage scale. Different colors in the stacked bars indicate different life cycle stages of NCA LIBs.

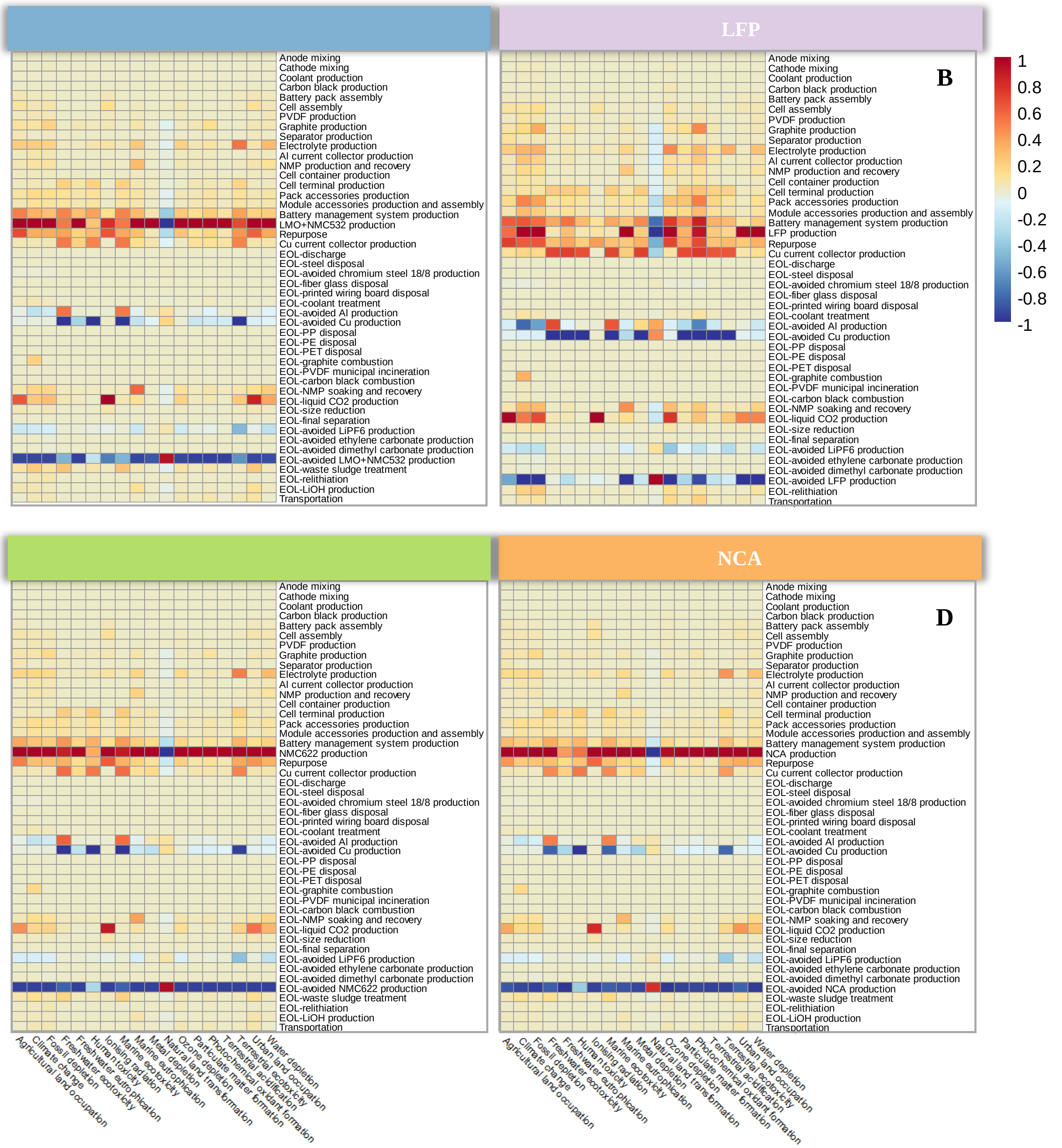


**Fig. S19. Full-spectrum environmental hotspots for LFP, LMO/NMC532, NMC622, and NCA LIBs with direct cathode recycling.** (**A**) Full-spectrum environmental hotspots for LFP with direct cathode recycling. (**B**) Full-spectrum environmental hotspots for LMO/NMC532 with direct cathode recycling. (**C**) Full-spectrum environmental hotspots for NMC622 with direct cathode recycling. (**D**) Full-spectrum environmental hotspots for NCA with direct cathode recycling. Use phases are excluded from the system boundary. Colors represent values corresponding to the environmental impacts of each process under each impact category.

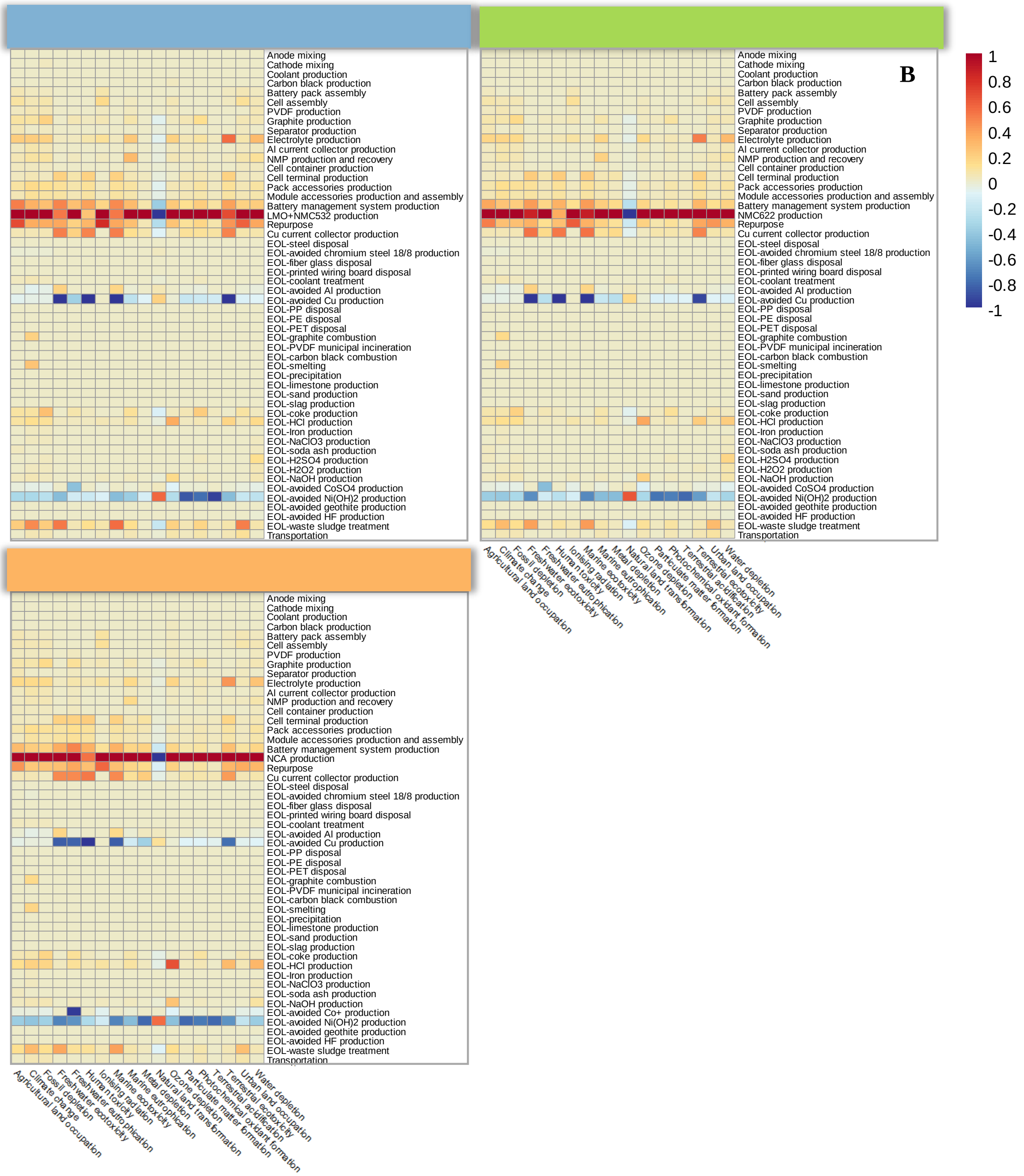


**Fig. S20. Full-spectrum environmental hotspots for LFP, LMO/NMC532, NMC622, and NCA LIBs with pyrometallurgical recycling.** (**A**) Full-spectrum environmental hotspots for LFP with pyrometallurgical recycling. (**B**) Full-spectrum environmental hotspots for LMO/NMC532 with pyrometallurgical recycling. (**C**) Full-spectrum environmental hotspots for NMC622 with pyrometallurgical recycling. (**D**) Full-spectrum environmental hotspots for NCA with direct cathode recycling. Use phases are excluded from the system boundary. Colors represent values corresponding to the environmental impacts of each process under each impact category.

## Sensitivity Analyses

In addition to electricity generation, LIB recycling affects the most on the life cycle environmental impacts. In this section, we assess the variability in life cycle environmental impacts of LIBs to recycling parameters, including recovery rate of cathode active materials, $Ni(OH)_2$, Co ion solution, other metals, graphite, $LiPF_6$, and chemical agents, as listed in Tables S18–S21. The upper and lower bound of these recycling parameters are extracted from previous LCA studies and experimental literature (*30, 112*). Since direct cathode recycling of LFP adopts a solid phase sintering method, which involves no washing, filtration, or separation steps, the recovery rate of LFP is assumed to be 100% and is not investigated in this sensitivity analysis. It is worth mentioning that there are three baseline values for copper recovery. To be specific, 98.7%, 98.3%, and 97.8% represent the recovery rates of copper dismantled from the LIB pack, LIB modules and negative terminals of LIB cells, copper sieved from the anode of LIB cells, and copper recovered from alloy leaching and precipitation following smelting process of pyrometallurgical recycling, respectively (*104, 111, 112*).

Results of sensitivity analyses for recycling parameters are shown in Figs. S19–S21. The results suggest that the recovery rate of cathode active materials, aluminum, and NMP is crucial to both the carbon footprint and energy performances of these LIBs. Besides these, improving the recovery rate of citric acid is important to reduce the carbon footprint and CED of hydrometallurgical recycling. The recovery rate of $Ni(OH)_2$ is also an influential recycling parameter to both the carbon footprint and CED of LIBs. Moreover, graphite recovery instead of combustion can largely reduce the carbon footprint of hydrometallurgical and direct cathode recycling for all types of LIBs. Notably, even under the best-case scenarios, the carbon footprint mitigation potential of pyrometallurgical and hydrometallurgical recycling is not as good as that of direct cathode recycling under the worst-case scenarios. The results highlight the need to achieve high recovery rates of these parameters during up-scaled recycling.

**Table S19. Sensitivity analyses on recycling parameters of LFP (*30, 112*).**

| Recycling method | Parameter | Baseline | Low | High |
|---|---|---|---|---|
| Hydrometallurgical recycling | Recovery rate of chromium steel 18/8 | 92% | 90% | 99.9% |
| | Recovery rate of aluminium | 95% | 70% | 99.9% |
| | Recovery rate of copper | 98.7%98.3% | 80% | 99.9% |
| | Recovery rate of graphite | 0% | - | 76.9% |
| | Recovery rate of LFP | 95% | 60% | 99.9% |
| | Recovery rate of NMP | 98% | 80% | 99.9% |
| Direct cathode recycling | Recovery rate of chromium steel 18/8 | 92% | 90% | 99.9% |
| | Recovery rate of aluminium | 95% | 70% | 99.9% |
| | Recovery rate of copper | 98.7%/98.3% | 80% | 99.9% |
| | Recovery rate of graphite | 0% | - | 76.9% |
| | Recovery rate of $LiPF_6$ | 100% | 70% | - |
| | Recovery rate of NMP | 98% | 80% | 99.9% |

## Table S20. Sensitivity analyses on recycling parameters of LMO/NMC532 (*30, 112*).

| Recycling method | Parameter | Baseline | Low | High |
|---|---|---|---|---|
| Hydrometallurgical recycling | Recovery rate of chromium steel 18/8 | 92% | 90% | 99.9% |
| | Recovery rate of aluminium | 95% | 70% | 99.9% |
| | Recovery rate of copper | 98.7%98.3% | 80% | 99.9% |
| | Recovery rate of graphite | 0% | - | 76.9% |
| | Recovery rate of NMC532 | 90.33% | 60% | 99.9% |
| | Conversion efficiency of $Mn_2O_3$ | 100% | 90% | - |
| | Recovery rate of citric acid | 90% | 80% | 99.9% |
| | Recovery rate of NMP | 98% | 80% | 99.9% |
| Direct cathode recycling | Recovery rate of chromium steel 18/8 | 92% | 90% | 99.9% |
| | Recovery rate of aluminium | 95% | 70% | 99.9% |
| | Recovery rate of copper | 98.7%/98.3% | 80% | 99.9% |
| | Recovery rate of graphite | 0% | - | 76.9% |
| | Recovery rate of NMC532 | 95% | 60% | 99.9% |
| | Recovery rate of $LiPF_6$ | 100% | 70% | - |
| | Recovery rate of NMP | 98% | 80% | 99.9% |
| Pyrometallurgical recycling | Recovery rate of chromium steel 18/8 | 92% | 90% | 99.9% |
| | Recovery rate of aluminium | 95% | 70% | 99.9% |
| | Recovery rate of copper | 98.7%/98.3%/97.78% | 80% | 99.9% |
| | Recovery rate of goethite | 98.99% | 60% | - |
| | Recovery rate of cobalt as $CoSO_4$ | 61.81% | 60% | 99.9% |
| | Recovery rate of nickel as $Ni(OH)_2$ | 95.71% | 60% | 99.9% |

## Table S21. Sensitivity analyses on recycling parameters of NMC622 (*30, 112*).

| Recycling method | Parameter | Baseline | Low | High |
|---|---|---|---|---|
| Hydrometallurgical recycling | Recovery rate of chromium steel 18/8 | 92% | 90% | 99.9% |
| | Recovery rate of aluminium | 95% | 70% | 99.9% |
| | Recovery rate of copper | 98.7%/98.3% | 80% | 99.9% |
| | Recovery rate of graphite | 0% | - | 76.9% |
| | Recovery rate of NMC622 | 99.7% | 60% | 99.9% |
| | Recovery rate of NMP | 98% | 80% | 99.9% |
| Direct cathode recycling | Recovery rate of chromium steel 18/8 | 92% | 90% | 99.9% |
| | Recovery rate of aluminium | 95% | 70% | 99.9% |
| | Recovery rate of copper | 98.7%/98.3% | 80% | 99.9% |
| | Recovery rate of graphite | 0% | - | 76.9% |
| | Recovery rate of NMC622 | 95% | 60% | 99.9% |
| | Recovery rate of $LiPF_6$ | 100% | 70% | - |
| | Recovery rate of NMP | 98% | 80% | 99.9% |
| Pyrometallurgical recycling | Recovery rate of chromium steel 18/8 | 92% | 90% | 99.9% |
| | Recovery rate of aluminium | 95% | 70% | 99.9% |
| | Recovery rate of copper | 98.7%/98.3%/97.78% | 80% | 99.9% |
| | Recovery rate of goethite | 98.99% | 60% | - |
| | Recovery rate of cobalt as $CoSO_4$ | 61.81% | 60% | 99.9% |
| | Recovery rate of nickel as $Ni(OH)_2$ | 95.71% | 60% | 99.9% |

**Table S22. Sensitivity analyses on recycling parameters of NCA (*30, 112*).**

| Recycling method | Parameter | Baseline | Low | High |
|---|---|---|---|---|
| Hydrometallurgical recycling | Recovery rate of chromium steel 18/8 | 92% | 90% | 99.9% |
| | Recovery rate of aluminium | 95% | 70% | 99.9% |
| | Recovery rate of copper | 98.7%/98.3% | 80% | 99.9% |
| | Recovery rate of graphite | 0% | - | 76.9% |
| | Recovery rate of NCA | 95.6% | 60% | 99.9% |
| | Recovery rate of NMP | 98% | 80% | 99.9% |
| Direct cathode recycling | Recovery rate of chromium steel 18/8 | 92% | 90% | 99.9% |
| | Recovery rate of aluminium | 95% | 70% | 99.9% |
| | Recovery rate of copper | 98.7%/98.3% | 80% | 99.9% |
| | Recovery rate of graphite | 0% | - | 76.9% |
| | Recovery rate of NCA | 95% | 60% | 99.9% |
| | Recovery rate of $LiPF_6$ | 100% | 70% | - |
| | Recovery rate of NMP | 98% | 80% | 99.9% |
| Pyrometallurgical recycling | Recovery rate of chromium steel 18/8 | 92% | 90% | 99.9% |
| | Recovery rate of aluminium | 95% | 70% | 99.9% |
| | Recovery rate of copper | 98.7%/98.3%/97.78% | 80% | 99.9% |
| | Recovery rate of goethite | 98.99% | 60% | - |
| | Recovery rate of cobalt as $CoCl_2$ | 59.27% | - | 99.9% |
| | Recovery rate of nickel as $Ni(OH)_2$ | 95.81% | 60% | 99.9% |

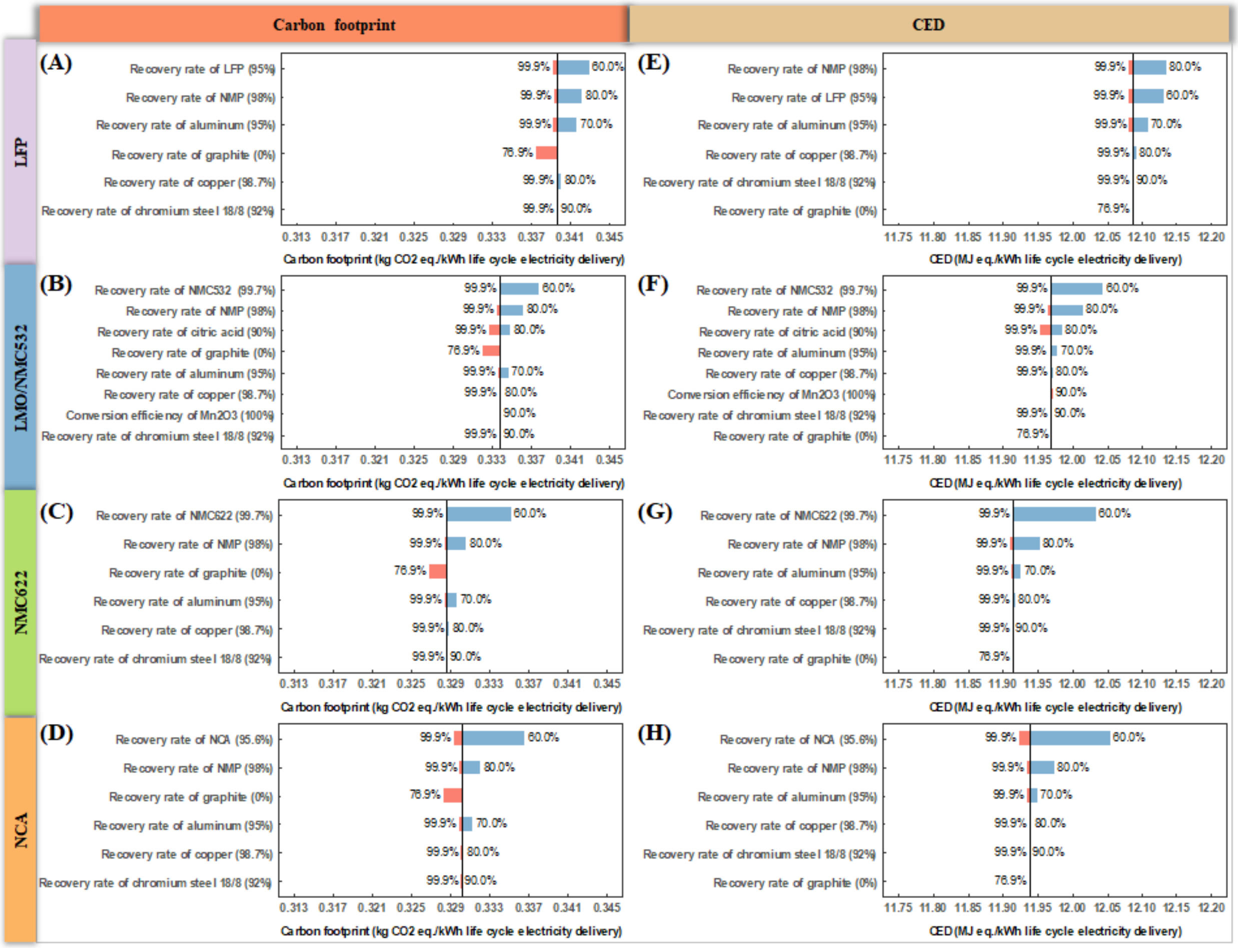


**Fig. S21. Sensitivity analyses for hydrometallurgical recycling on recycling parameters.** (**A**) Sensitivity analysis results for LFP LIBs in terms of carbon footprint. (**B**) Sensitivity analysis results for LMO/NMC532 LIBs in terms of carbon footprint. (**C**) Sensitivity analysis results for

NMC622 LIBs in terms of carbon footprint. (**D**) Sensitivity analysis results for NCA LIBs in terms of carbon footprint. (**E**) Sensitivity analysis results for LFP LIBs in terms of CED. (**F**) Sensitivity analysis results for LMO/NMC532 LIBs in terms of CED. (**G**) Sensitivity analysis results for NMC622 LIBs in terms of CED. (**H**) Sensitivity analysis results for NCA LIBs in terms of CED.

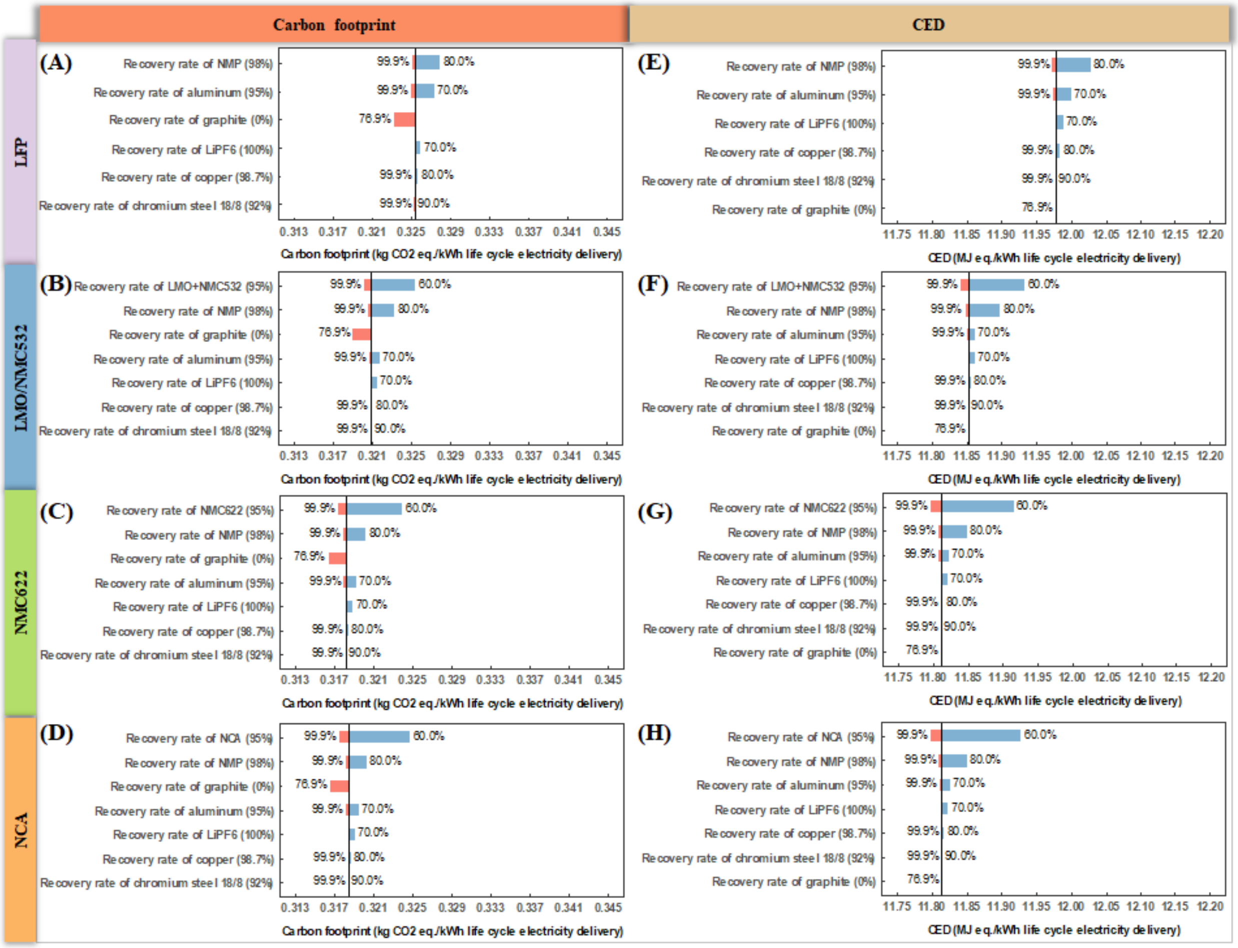


**Fig. S22. Sensitivity analyses for direct cathode recycling on recycling parameters.** (**A**) Sensitivity analysis results for LFP LIBs in terms of carbon footprint. (**B**) Sensitivity analysis results for LMO/NMC532 LIBs in terms of carbon footprint. (**C**) Sensitivity analysis results for NMC622 LIBs in terms of carbon footprint. (**D**) Sensitivity analysis results for NCA LIBs in terms of carbon footprint. (**E**) Sensitivity analysis results for LFP LIBs in terms of CED. (**F**) Sensitivity analysis results for LMO/NMC532 LIBs in terms of CED. (**G**) Sensitivity analysis results for NMC622 LIBs in terms of CED. (**H**) Sensitivity analysis results for NCA LIBs in terms of CED.

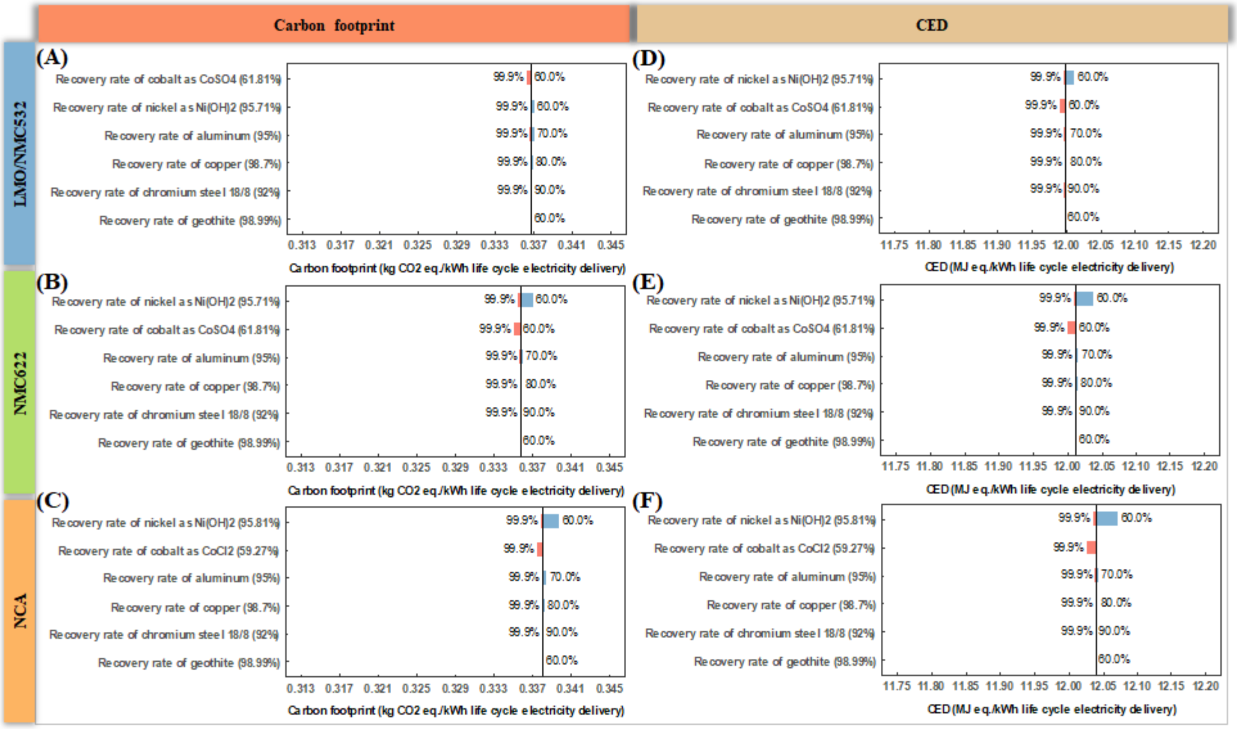


**Fig. S23. Sensitivity analyses for pyrometallurgical recycling on recycling parameters.** (A) Sensitivity analysis results for LMO/NMC532 LIBs in terms of carbon footprint. (B) Sensitivity analysis results for NMC622 LIBs in terms of carbon footprint. (C) Sensitivity analysis results for NCA LIBs in terms of carbon footprint. (D) Sensitivity analysis results for LMO/NMC532 LIBs in terms of CED. (E) Sensitivity analysis results for NMC622 LIBs in terms of CED. (F) Sensitivity analysis results for NCA LIBs in terms of CED.

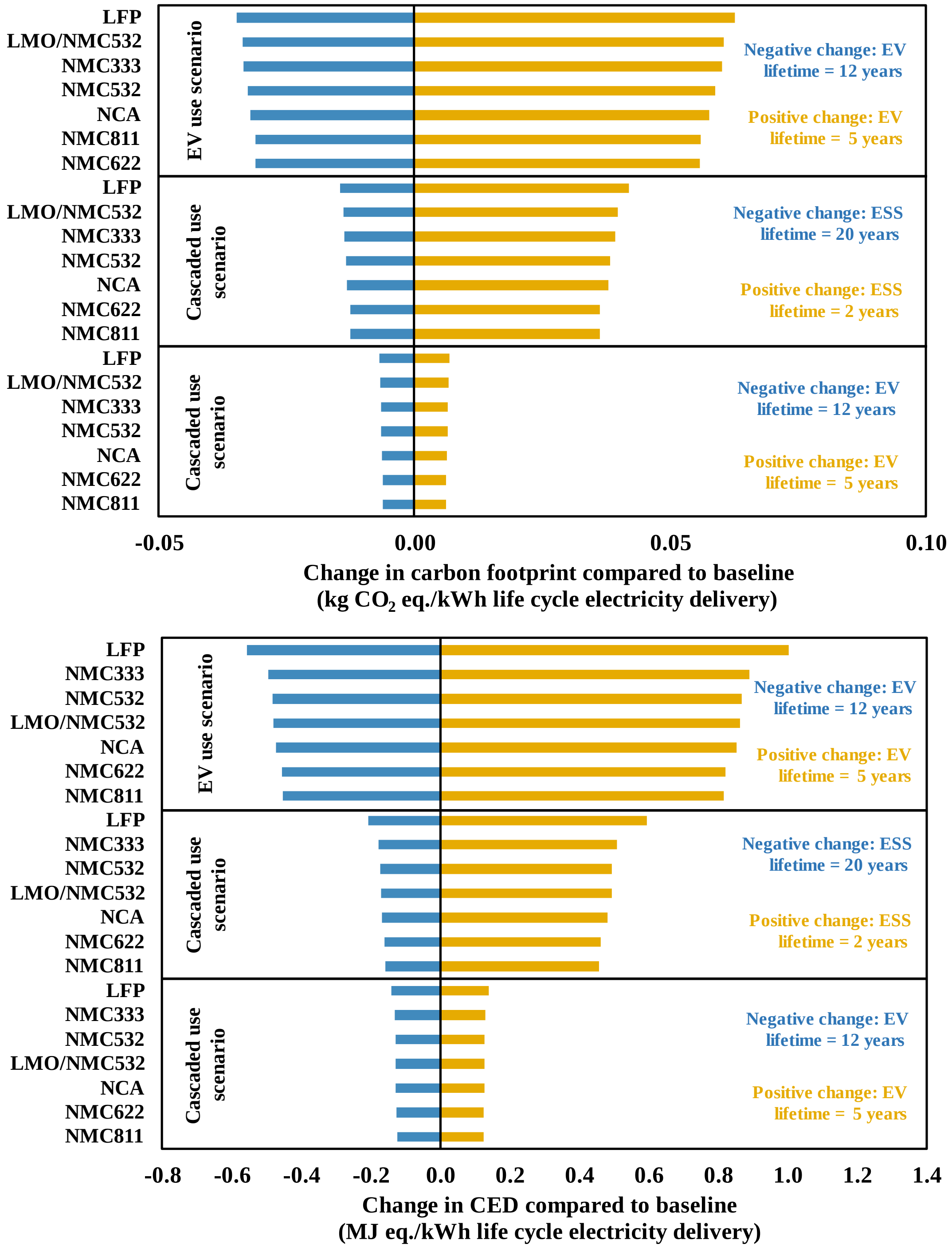


**Fig. S24. Sensitivity analysis for the lifetime of EV use and ESS use.**

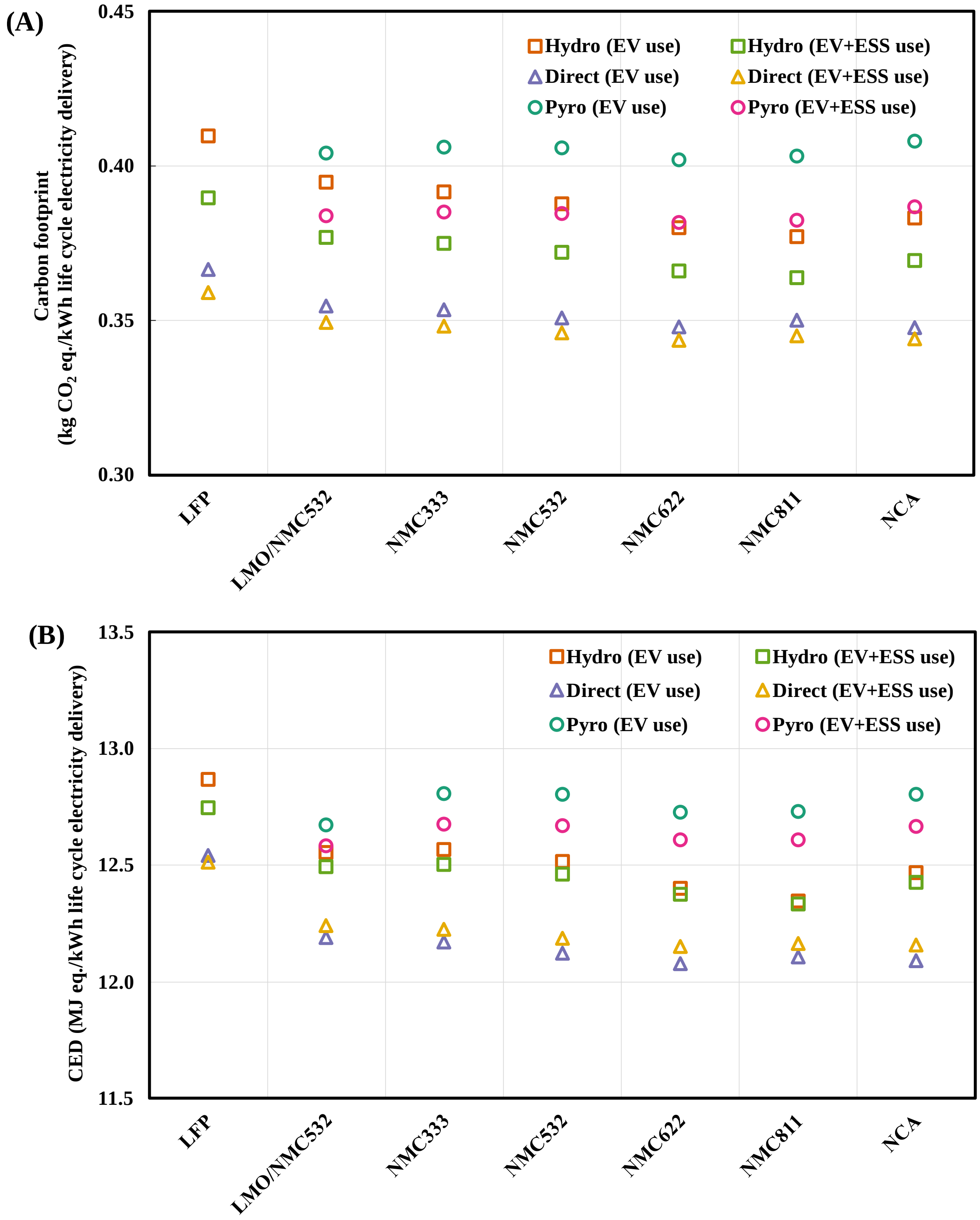


**Fig. S25. Result of carbon footprint and CED for a 2-year second life in ESS.**

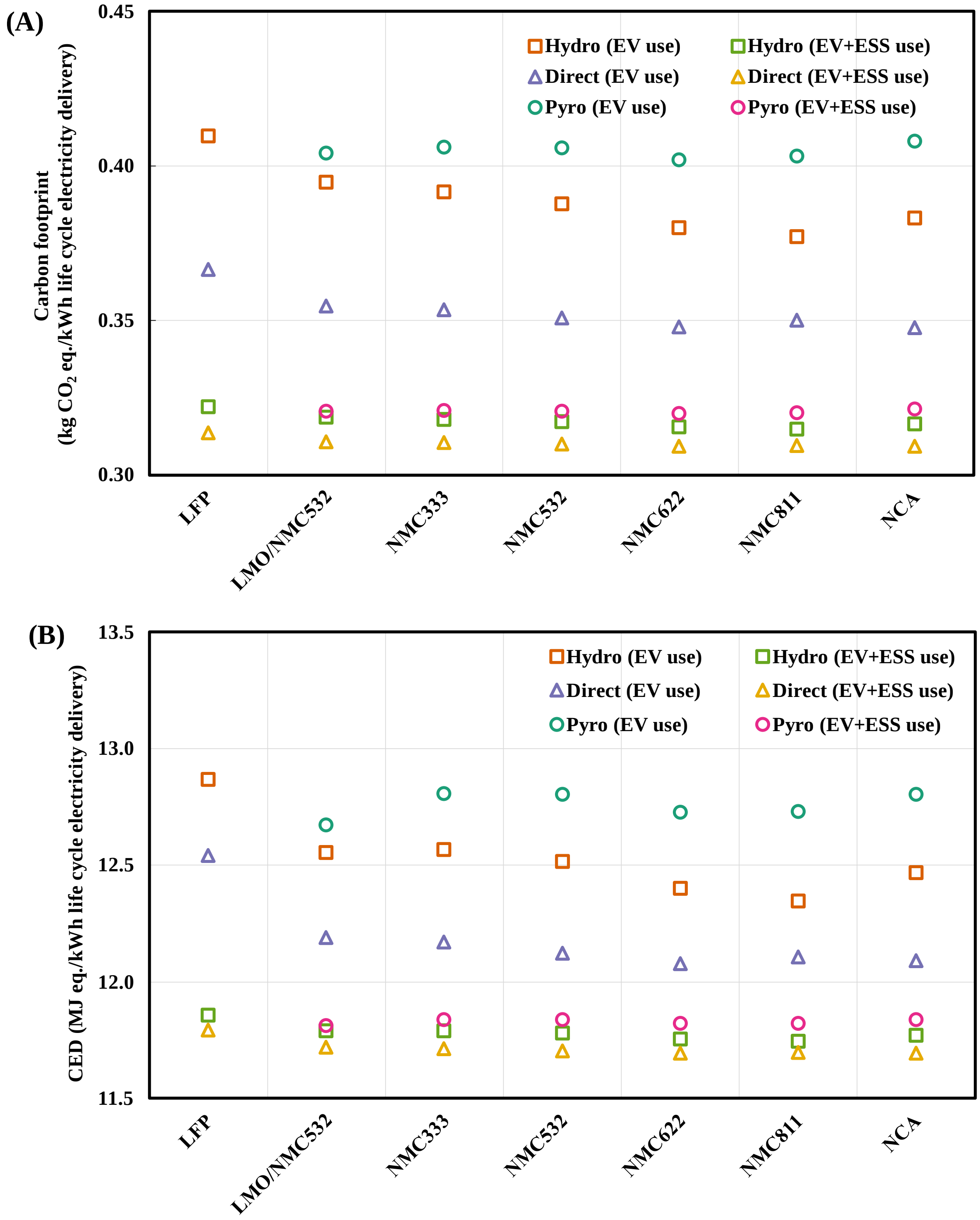


**Fig. S26. Result of carbon footprint and CED for a 20-year second life in ESS.**

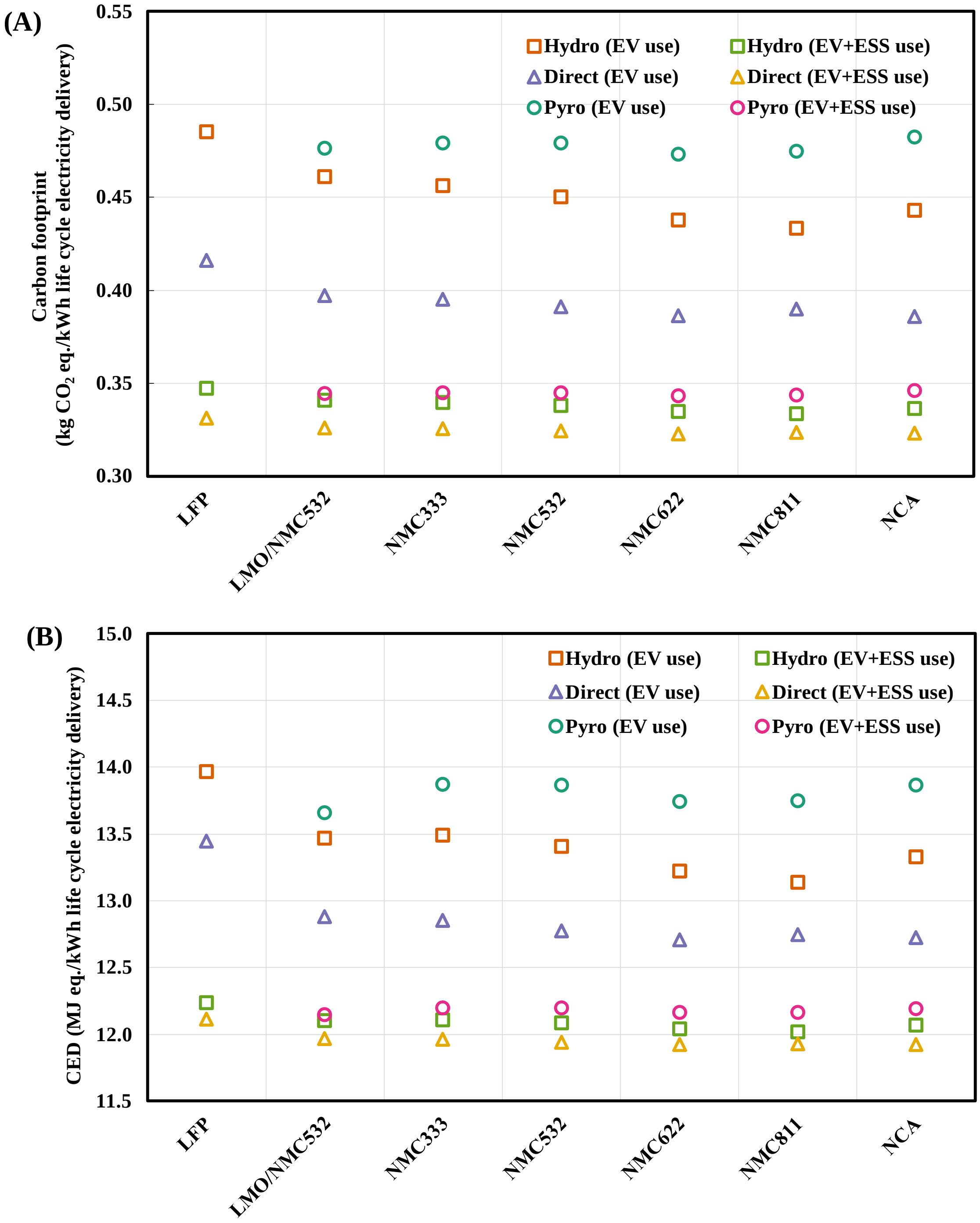


**Fig. S27. Result of carbon footprint and CED for 5-year EV use.**

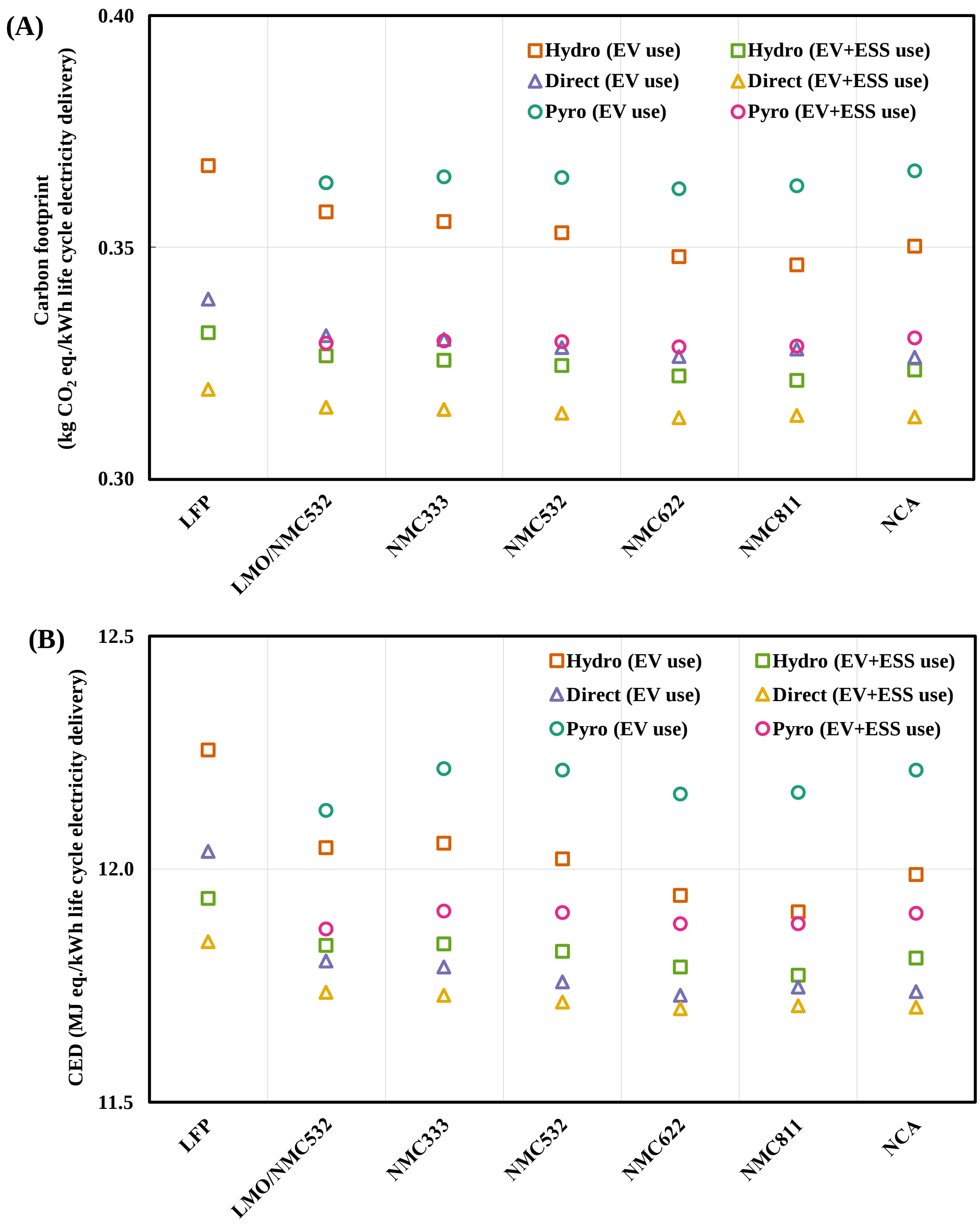


**Fig. S28. Result of carbon footprint and CED for 12-year EV use.**

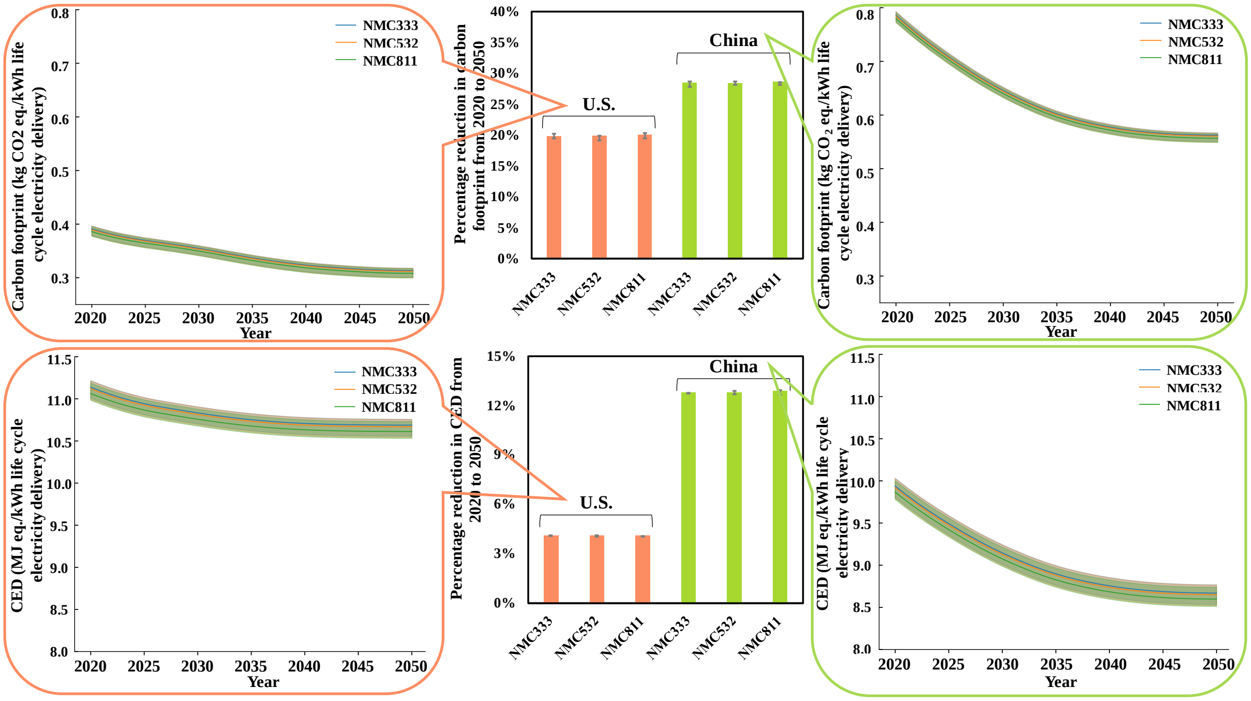


**Fig. S29. Sensitivity analysis of temporal and spatial variations in electricity generation from 2020 to 2050 in the U.S. and China for NMC333, NMC532, NMC622, and NMC811.**

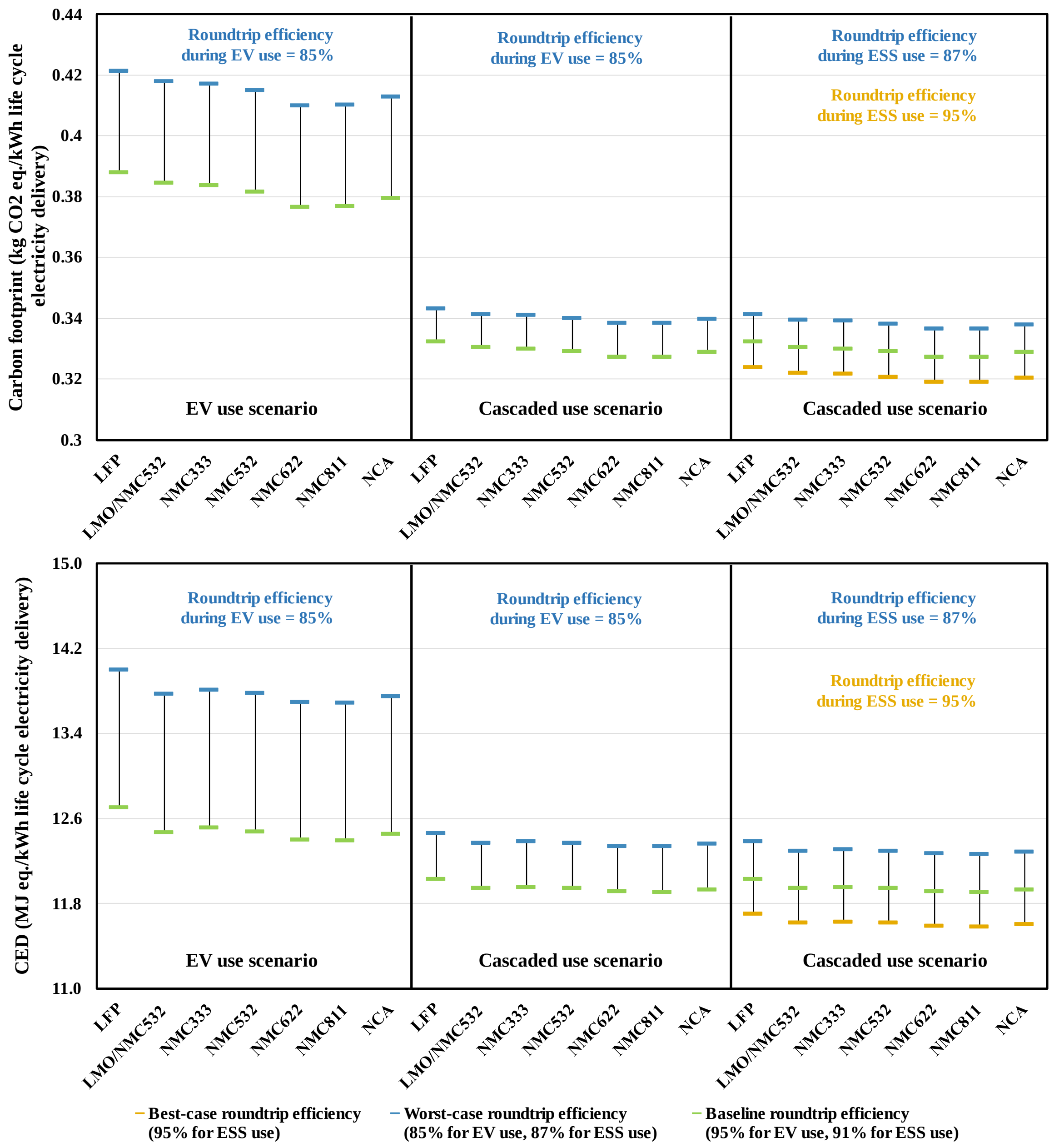


**Fig. S30. Sensitivity analysis of the roundtrip efficiency with the best and worst cases compared to the base case of carbon footprint and CED for LFP, LMO/NMC532, NMC333, NMC532, NMC622, NMC811, and NCA.** The best and worst cases of roundtrip efficiency are obtained from existing literature (*12*, *29*). The best-case scenario of the roundtrip efficiency during EV use is not considered because none of the existing studies adopted any values higher than its base-case value (95%).

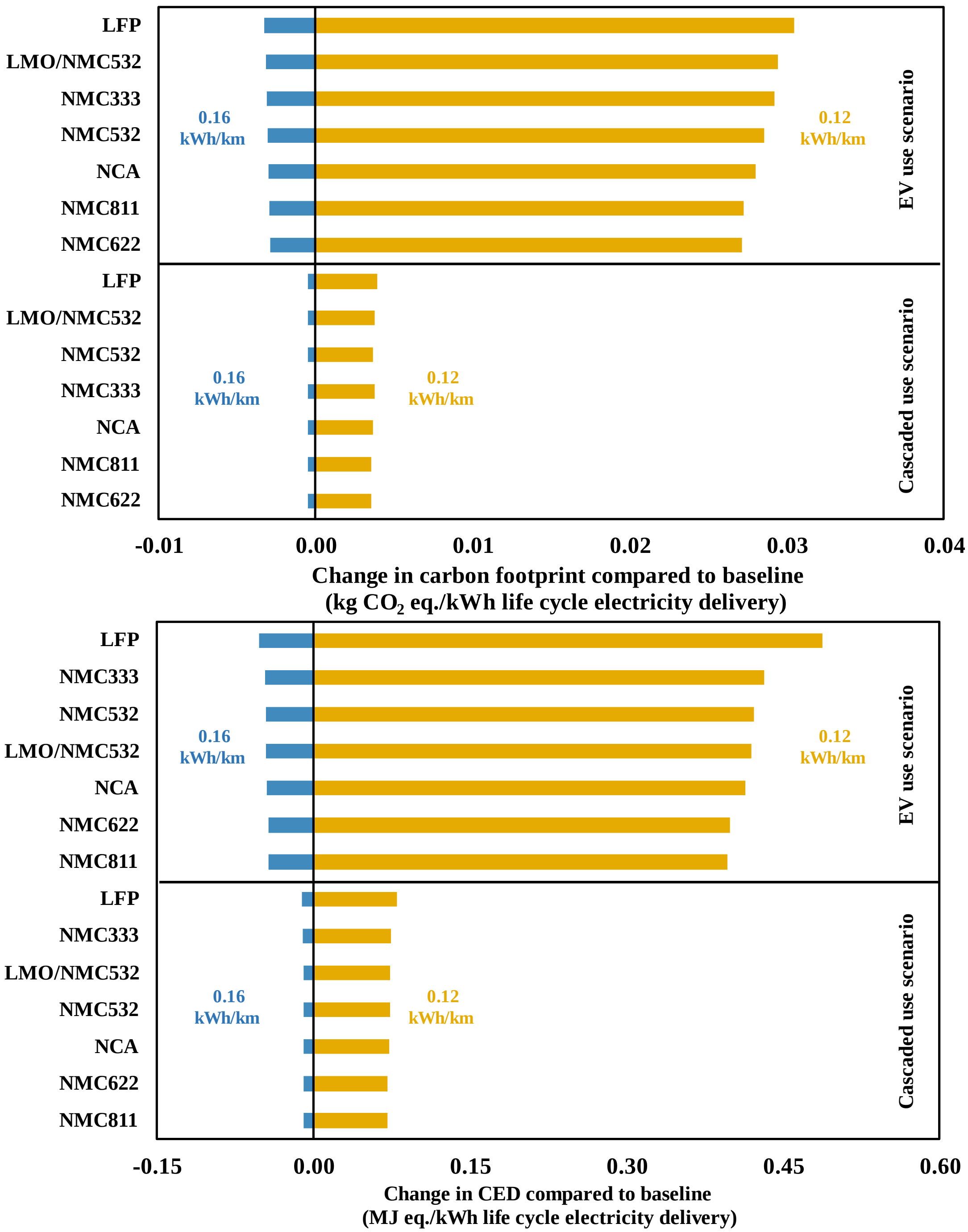


**Fig. S31. Sensitivity analysis of the energy consumption rate with the best and worst cases compared to the base case of carbon footprint and CED for LFP, LMO/NMC532, NMC333, NMC532, NMC622, NMC811, and NCA.** The best and worst cases of energy consumption rate are obtained from existing literature (*59*).

## Environmental profile of electricity generation

Fig. S32 shows the electricity generation by energy sources in NPCC used in this study (*113*). The energy generated from natural gas, nuclear and hydroelectric is responsible for 90% of the total energy generation. Natural gas, as a source that accounts for 44% of electricity generation, contributes to a large portion of fossil depletion, climate change, and ozone depletion; Nuclear power accounts for 30% of the NPCC electricity generation and contributes to major environmental impacts of ionizing radiation, marine ecotoxicity, and ozone depletion; Hydroelectricity accounts for 15% of the NPCC electricity generation and is responsible for the majority of water depletion. In addition, although biomass results in only 4.5% of electricity generation, it could explain most environmental impacts from the urban land occupation and terrestrial ecotoxicity. Therefore, the environmental impacts for electricity consumption (especially the use phase) are largely determined by characteristics of the environmental profiles of these energy sources. Some minor energy sources such as coal also have an influential impact on the environmental profile due to its large damage to ecosystems and human health.

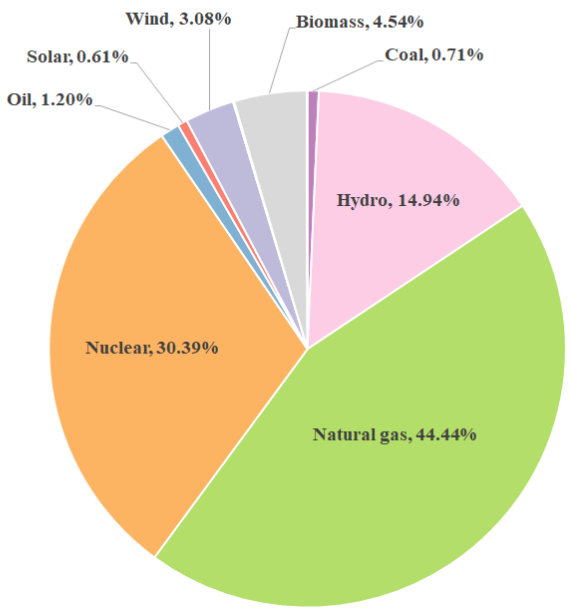


**Fig. S32. Electricity generation by energy sources of NPCC in 2018.**

The electricity generation by energy source from 2020 to 2050 in the United States and China is presented on a percentage basis in Fig. S35. This data is used to assess the impact of temporal and geographical variation in electricity generation on the environmental performance of reused automotive LIBs.

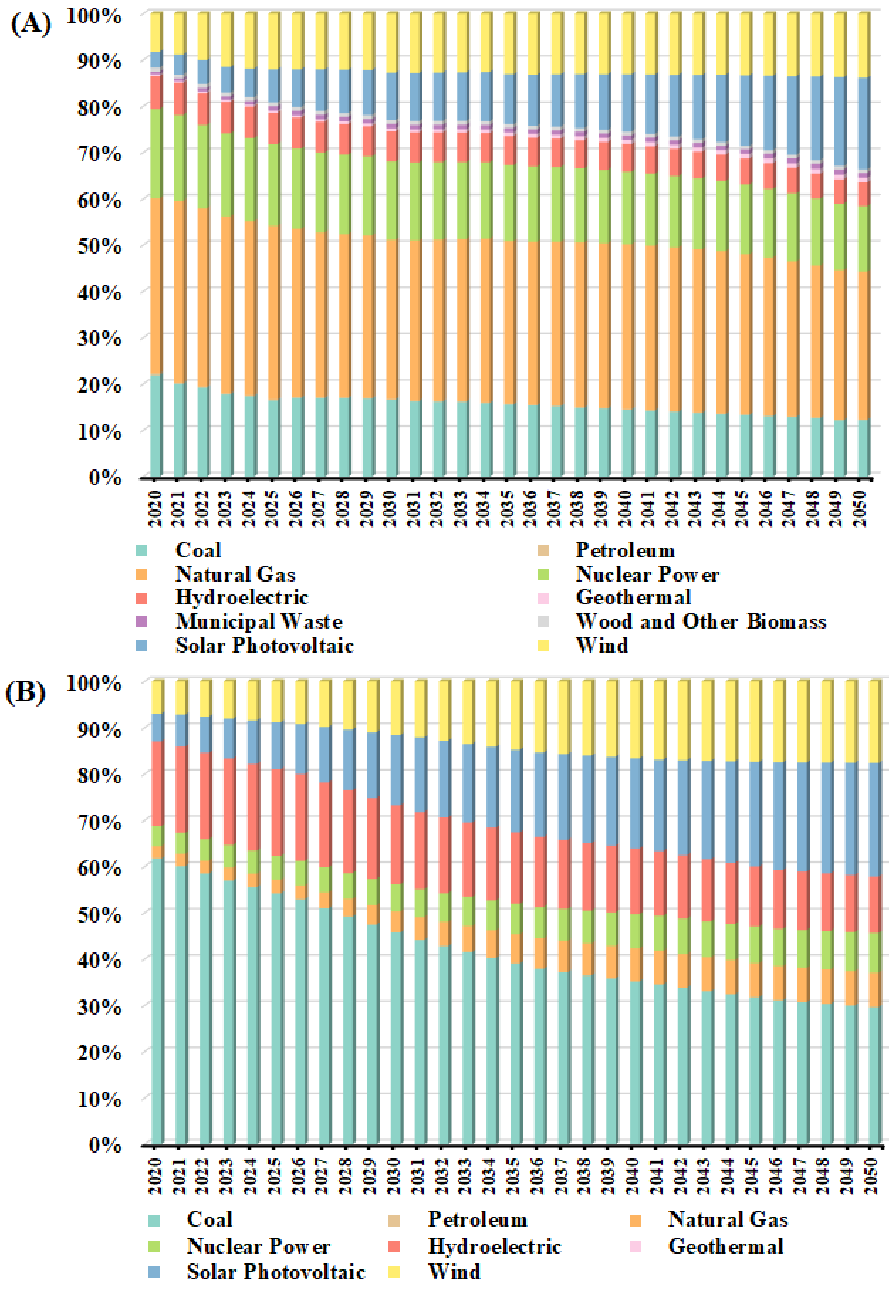


**Fig. S33. Electricity generation by energy sources from 2020 to 2050.** (**A**) Electricity generation by energy sources in the United States from 2020 to 2050 (*55*). (**B**) Electricity generation by energy sources in China (*54*) from 2020 to 2050.

## Table S23. Transportation of metal and chemicals on a ton basis (*21, 31*).

| Component | Water (km) | Rail (km) | Road (km) |
|---|---|---|---|
| $NiSO_4$ | 0 | 500 | 50 |
| $CoSO_4$ | 0 | 1500 | 50 |
| $MnSO_4$ | 0 | 1350 | 50 |
| $Al_2(SO_4)_3$ | 0 | 600 | 50 |
| Steel | 8250 | 600 | 50 |
| Iron | 8250 | 600 | 50 |
| Aluminium | 0 | 700 | 50 |
| Copper | 0 | 650 | 50 |
| Graphite | 8250 | 600 | 50 |
| PVDF | 0 | 500 | 50 |
| $LiPF_6$ | 0 | 500 | 50 |
| EC | 0 | 500 | 50 |
| DMC | 0 | 500 | 50 |
| PP | 0 | 500 | 50 |
| PE | 0 | 500 | 50 |
| PET | 0 | 500 | 50 |
| NaOH | 0 | 500 | 50 |
| $NH_3$ | 0 | 500 | 50 |
| ACN | 0 | 500 | 50 |
| PC | 0 | 500 | 50 |
| $H_2O_2$ | 0 | 500 | 50 |
| Citric acid | 0 | 500 | 50 |
| $H_2SO_4$ | 0 | 500 | 50 |
| HCl | 0 | 500 | 50 |
| $H_3PO_4$ | 0 | 500 | 50 |
| $FeSO_4$ | 0 | 500 | 50 |
| $Na_2CO_3$ | 0 | 500 | 50 |
| $NaClO_3$ | 0 | 500 | 50 |
| $LI_2CO_3$ | 0 | 500 | 50 |
| LiOH | 0 | 500 | 50 |
| $Mn_2O_3$ | 0 | 0 | 500 |
| Cathode active material | 0 | 0 | 20 |
| EOL LIBs (after EV use) | 0 | 0 | 450 |
| EOL LIBs (after stationary ESS use) | 0 | 0 | 500 |

## Table S24. Yield rates for battery materials and cells (*34, 114*).

| Component | Yield rate |
|---|---|
| Cathode materials | 92.2% |
| Anode materials | 92.2% |
| Aluminum current collector | 90.2% |
| Copper current collector | 90.2% |
| NMP recovery | 98% |
| Separator | 98% |
| Electrolyte | 94% |
| Cells | 95% |

**Table S25. Characterization factors extracted from the Ecoinvent database (*80*). The impact categories are global warming potential (GWP), cumulative energy demand (CED), Agriculture Land Occupation (ALO), Climate Change (CC), Fossil Depletion (FD), Freshwater Ecotoxicity (FET), Freshwater Eutrophication (FE), Human Toxicity (HT), Ionizing Radiation (IR), Marine Ecotoxicity (MET), Marine Eutrophication (ME), Metal Resource Depletion (MRD), Natural Land Transformation (NLT), Ozone Depletion (OD), Particulate matter formation (PMF), Photochemical Oxidant Formation (POF), Terrestrial Acidification (TA), Terrestrial Ecotoxicity (TET), Urban Land Occupation (ULO), Water Depletion (WD).**

| Item | Unit Process | Value | Unit | GWP100 | CED | ReCiPe Midpoint (H) | | | | | | | | | | | | | | | | | |
|---|---|---|---|---|---|---|---|---|---|---|---|---|---|---|---|---|---|---|---|---|---|---|---|
| | | | | | | ALO | CC | FD | FET | FE | HT | IR | MET | ME | MRD | NLT | OD | PMF | POF | TA | TET | ULO | WD |
| Heat | heat production, natural gas, at industrial furnace >100kW, RoW | 1 | MJ | 6.92E-02 | 1.14E+00 | 8.74E-05 | 6.84E-02 | 3.29E-02 | 3.63E-04 | 1.04E-06 | 3.13E-03 | 2.66E-04 | 1.41E-04 | 2.10E-06 | 1.15E-04 | -1.30E-07 | 5.32E-09 | 2.00E-05 | 6.85E-05 | 5.86E-05 | 4.55E-06 | 2.39E-05 | 1.43E-05 |
| Decarbonized water | water production, decarbonised, US | 1 | kg | 8.94E-05 | 1.68E-03 | 5.41E-06 | 8.75E-05 | 2.97E-05 | 3.03E-06 | 2.33E-07 | 9.66E-05 | 2.49E-05 | 2.72E-06 | 7.16E-07 | 2.43E-06 | -7.97E-09 | 8.72E-12 | 2.70E-07 | 2.20E-07 | 2.75E-07 | 8.10E-09 | 4.47E-06 | 7.40E-06 |
| Wastewater treatment | treatment of wastewater, average, capacity 1E9l/year, RoW | 1 | m3 | 5.70E-01 | 6.68E+00 | 2.20E-02 | 5.48E-01 | 1.40E-01 | 2.98E-02 | 1.13E-03 | 3.01E-01 | 3.31E-02 | 2.68E-02 | 2.07E-02 | 6.22E-02 | -3.28E-04 | 3.57E-08 | 2.17E-03 | 2.62E-03 | 4.72E-03 | 3.31E-04 | 2.69E-02 | 1.04E-01 |
| Wastewater treatment plant | wastewater treatment facility construction, capacity 1E9l/year, RoW | 1 | unit | 5.49E+06 | 5.49E+07 | 2.41E+05 | 5.12E+06 | 1.19E+06 | 4.38E+05 | 1.89E+03 | 2.85E+06 | 2.20E+05 | 3.92E+05 | 1.09E+03 | 1.13E+06 | -7.23E+02 | 2.95E-01 | 1.50E+04 | 2.27E+04 | 2.11E+04 | 7.23E+02 | 2.40E+05 | 2.96E+04 |
| $N_2$ | air separation, cryogenic, RoW | 1 | kg | 4.40E-01 | 6.21E+00 | 1.44E-02 | 4.33E-01 | 1.23E-01 | 6.59E-03 | 1.98E-04 | 1.47E-01 | 4.37E-02 | 5.88E-03 | 8.71E-05 | 3.41E-03 | -2.26E-05 | 1.45E-08 | 1.15E-03 | 1.15E-03 | 1.69E-03 | 1.55E-05 | 2.05E-03 | 1.04E-02 |
| Steam | steam production, in chemical industry, RoW | 1 | kg | 3.34E-01 | 4.47E+00 | 2.37E-03 | 3.30E-01 | 1.13E-01 | 1.80E-03 | 4.00E-05 | 4.48E-02 | 8.59E-03 | 1.54E-03 | 2.66E-05 | 8.89E-04 | -6.54E-06 | 2.71E-08 | 3.44E-04 | 5.92E-04 | 9.72E-04 | 4.16E-05 | 7.21E-04 | 2.63E-04 |
| Steam generation system | steam generation system construction, solar tower power plant, 20 MW, RoW | 1 | unit | 6.07E+05 | 6.22E+06 | 2.20E+04 | 5.31E+05 | 1.43E+05 | 6.78E+04 | 3.15E+02 | 3.69E+05 | 2.54E+04 | 6.17E+04 | 1.57E+02 | 2.42E+05 | -4.54E+01 | 2.91E-02 | 3.65E+03 | 3.51E+03 | 9.82E+03 | 1.19E+02 | 6.68E+03 | 5.43E+03 |
| NMP | N-methyl-2-pyrrolidone production, RoW | 1 | kg | 6.33E+00 | 1.21E+02 | 3.86E-01 | 6.18E+00 | 2.80E+00 | 2.13E-01 | 2.19E-03 | 2.22E+00 | 5.27E-01 | 1.86E-01 | 1.77E-03 | 3.29E-01 | -3.28E-04 | 5.36E-07 | 1.21E-02 | 1.91E-02 | 2.64E-02 | 5.37E-04 | 4.03E-02 | 2.72E-01 |
| $LiMn_2O_4$ | lithium manganese oxide production, GLO | 1 | kg | 4.17E+00 | 6.42E+01 | 2.67E-01 | 4.11E+00 | 1.21E+00 | 1.34E-01 | 2.01E-03 | 1.61E+00 | 6.86E-01 | 1.20E-01 | 1.89E-03 | 7.41E+01 | -5.75E-04 | 3.06E-07 | 6.94E-03 | 1.08E-02 | 1.49E-02 | 2.20E-04 | 5.69E-02 | 8.27E-02 |
| $FeSO_4$ | iron sulfate production, RoW | 1 | kg | 2.31E-01 | 3.42E+00 | 2.27E-02 | 2.27E-01 | 6.45E-02 | 2.15E-02 | 1.36E-04 | 1.37E-01 | 2.57E-02 | 1.88E-02 | 5.60E-05 | 6.33E-02 | -3.21E-05 | 7.88E-09 | 6.41E-04 | 6.96E-04 | 9.89E-04 | 2.45E-05 | 3.23E-02 | 1.47E-03 |
| $H_3PO_4$ | purification of wet-process phosphoric acid to industrial grade, product in 85% solution state, RoW | 1 | kg | 1.37E+00 | 2.16E+01 | 1.21E-01 | 1.35E+00 | 4.97E-01 | 1.66E-01 | 1.80E-03 | 1.14E+00 | 1.03E-01 | 1.49E-01 | 3.32E-04 | 1.62E+00 | 2.60E-05 | 1.44E-07 | 5.32E-03 | 5.93E-03 | 1.45E-02 | 6.77E-04 | 5.29E-01 | 1.27E-01 |
| $Li_2CO_3$ | lithium carbonate production, from concentrated brine, GLO | 1 | kg | 2.12E+00 | 3.03E+01 | 2.83E-01 | 2.09E+00 | 6.24E-01 | 9.75E-02 | 1.99E-03 | 1.19E+00 | 1.67E-01 | 8.71E-02 | 5.84E-03 | 5.07E-01 | -1.07E-03 | 1.73E-07 | 5.84E-03 | 9.97E-03 | 1.48E-02 | 2.97E-04 | 2.51E-01 | 4.17E-02 |
| LiOH | lithium hydroxide production, GLO | 1 | kg | 5.94E+00 | 7.18E+01 | 5.77E-01 | 5.78E+00 | 1.46E+00 | 2.83E-01 | 3.78E-03 | 2.98E+00 | 3.92E-01 | 2.54E-01 | 9.56E-03 | 9.00E-01 | -1.78E-03 | 4.29E-07 | 1.30E-02 | 2.11E-02 | 3.14E-02 | 5.76E-04 | 1.10E-01 | 7.56E-02 |
| $NiSO_4$ | nickel sulfate production, GLO | 1 | kg | 6.06E+00 | 8.96E+01 | 4.79E-01 | 5.91E+00 | 1.69E+00 | 4.80E+00 | 8.43E-03 | 1.39E+01 | 4.69E-01 | 4.46E+00 | 3.91E-03 | 1.80E+01 | -2.06E-03 | 3.63E-07 | 2.37E-01 | 1.33E-01 | 1.09E+00 | 4.07E-03 | 1.07E-01 | 5.65E-02 |
| $MnSO_4$ | manganese sulfate production, GLO | 1 | kg | 7.49E-01 | 1.51E+01 | 5.79E-02 | 7.30E-01 | 3.31E-01 | 6.42E-02 | 3.73E-04 | 1.91E+00 | 8.96E-02 | 5.73E-02 | 1.76E-04 | 4.34E+01 | -1.88E-04 | 5.17E-08 | 6.09E-03 | 4.61E-03 | 2.47E-02 | 9.76E-05 | 2.31E-02 | 5.85E-03 |
| NaOH | chlor-alkali electrolysis, membrane cell, RoW | 1 | kg | 1.32E+00 | 1.87E+01 | 6.64E-02 | 1.29E+00 | 3.62E-01 | 5.56E-02 | 6.48E-04 | 6.07E-01 | 1.33E-01 | 4.95E-02 | 3.59E-04 | 9.32E-02 | -9.19E-05 | 7.95E-07 | 3.52E-03 | 3.80E-03 | 5.66E-03 | 8.32E-05 | 1.04E-02 | 2.68E-02 |
| $Na_2SO_4$ | sodium sulfate production, from natural sources, RoW | 1 | kg | 1.85E-01 | 2.59E+00 | 1.55E-02 | 1.81E-01 | 4.91E-02 | 1.65E-02 | 1.05E-04 | 1.23E-01 | 1.70E-02 | 1.46E-02 | 3.99E-05 | 2.94E-02 | -1.91E-05 | 6.64E-09 | 5.22E-04 | 5.52E-04 | 9.23E-04 | 1.40E-05 | 1.78E-03 | 9.61E-04 |
| $NH_3$ | ammonia production, steam reforming, liquid, RoW | 1 | kg | 1.86E+00 | 3.66E+01 | 2.18E-02 | 1.84E+00 | 9.76E-01 | 4.17E-02 | 1.31E-04 | 3.51E-01 | 5.71E-02 | 3.50E-02 | 2.16E-04 | 7.62E-02 | -2.96E-05 | 2.39E-07 | 1.67E-03 | 3.17E-03 | 3.96E-03 | 3.17E-04 | 3.67E-03 | 5.57E-02 |
| $H_2SO_4$ | sulfuric acid production, RoW | 1 | kg | 9.46E-02 | 4.61E+00 | 1.19E-02 | 9.09E-02 | 1.22E-01 | 2.00E-02 | 6.12E-05 | 1.13E-01 | 7.66E-03 | 1.73E-02 | 4.26E-05 | 4.30E-02 | -1.45E-05 | 6.57E-09 | 1.93E-03 | 1.53E-03 | 8.68E-03 | 2.90E-05 | 1.55E-03 | 2.61E-02 |
| Sulfur | market for sulfur, GLO | 1 | kg | 1.94E-01 | 1.78E+01 | 1.13E-03 | 1.83E-01 | 5.05E-01 | 4.42E-03 | 1.63E-05 | 6.29E-02 | 7.04E-03 | 1.78E-03 | 2.45E-05 | 2.46E-03 | -3.37E-06 | 1.65E-08 | 2.05E-03 | 1.63E-03 | 9.77E-03 | 6.55E-05 | 1.68E-03 | 2.62E-04 |
| MgO | market for magnesium oxide, GLO | 1 | kg | 1.18E+00 | 3.63E+00 | 1.22E-02 | 1.16E+00 | 7.18E-02 | 2.78E-02 | 8.92E-05 | 4.20E-01 | 2.71E-02 | 2.40E-02 | 1.92E-04 | 5.26E-03 | -2.53E-05 | 1.60E-08 | 3.91E-03 | 2.17E-03 | 2.10E-03 | 5.00E-05 | 1.28E-02 | 1.65E-03 |
| $O_2$ | air separation, cryogenic, RoW | 1 | kg | 1.11E+00 | 1.57E+01 | 3.64E-02 | 1.09E+00 | 3.09E-01 | 1.66E-02 | 4.99E-04 | 3.70E-01 | 1.10E-01 | 1.48E-02 | 2.20E-04 | 8.60E-03 | -5.69E-05 | 3.67E-08 | 2.90E-03 | 2.91E-03 | 4.25E-03 | 3.91E-05 | 5.17E-03 | 2.63E-02 |
| $NH_4HCO_3$ | market for ammonium bicarbonate, RoW | 1 | kg | 1.68E+00 | 1.99E+01 | 6.30E-02 | 1.63E+00 | 4.60E-01 | 4.72E-02 | 4.58E-04 | 4.39E-01 | 6.87E-02 | 4.08E-02 | 3.26E-04 | 8.33E-02 | -1.01E-04 | 5.09E-08 | 2.47E-03 | 3.81E-03 | 4.80E-03 | 2.17E-04 | 7.82E-02 | 1.77E-02 |
| Chemical production, inorganic | chemical production, inorganic, GLO | 1 | kg | 2.00E+00 | 2.46E+01 | 1.06E-01 | 1.99E+00 | 5.31E-01 | 8.55E-02 | 6.93E-04 | 7.96E-01 | 1.31E-01 | 7.68E-02 | 5.20E-04 | 4.67E-01 | -1.12E-04 | 2.24E-07 | 4.67E-03 | 6.32E-03 | 1.25E-02 | 2.69E-04 | 3.62E-02 | 4.16E-02 |
| Kerosene | market for kerosene, RoW | 1 | kg | 4.79E-01 | 5.35E+01 | 5.87E-03 | 4.70E-01 | 1.22E+00 | 3.89E-03 | 3.70E-05 | 8.08E-02 | 2.26E-01 | 3.40E-03 | 9.37E-05 | 1.01E-02 | -1.65E-05 | 6.45E-07 | 1.38E-03 | 3.40E-03 | 4.56E-03 | 8.94E-05 | 7.51E-03 | 1.47E-03 |

| | | | | | | | | | | | | | | | | | | | | | | | |
|---|---|---|---|---|---|---|---|---|---|---|---|---|---|---|---|---|---|---|---|---|---|---|---|
| **Lime** | **market for lime, RoW** | **1** | **kg** | 4.34E-02 | 6.44E-01 | 2.49E-02 | 4.27E-02 | 1.32E-02 | 1.85E-03 | 1.63E-05 | 3.22E-02 | 2.23E-03 | 1.64E-03 | 1.21E-05 | 8.50E-03 | -3.52E-06 | 2.58E-09 | 1.68E-04 | 2.31E-04 | 2.54E-04 | 1.25E-05 | 1.49E-03 | 9.59E-04 |
| **Diesel, burned in building machine** | **diesel, burned in building machine, GLO** | **1** | **MJ** | 9.22E-02 | 1.34E+00 | 3.16E-04 | 9.05E-02 | 3.06E-02 | 3.78E-04 | 3.32E-06 | 4.41E-03 | 5.73E-03 | 3.46E-04 | 4.35E-05 | 1.39E-03 | -8.10E-07 | 1.56E-08 | 3.77E-04 | 1.28E-03 | 7.26E-04 | 3.90E-06 | 1.69E-04 | 7.48E-05 |
| **$Al_2(SO4)_3$** | **aluminium sulfate production, powder, RoW** | **1** | **kg** | 7.73E-01 | 1.14E+01 | 4.30E-02 | 7.55E-01 | 2.38E-01 | 6.46E-02 | 4.39E-04 | 5.94E-01 | 5.89E-02 | 5.81E-02 | 1.77E-04 | 3.75E-01 | -6.93E-05 | 4.30E-08 | 2.77E-03 | 3.20E-03 | 7.24E-03 | 8.69E-05 | 9.08E-03 | 1.32E-02 |
| **Carbon black** | **carbon black production, GLO** | **1** | **kg** | 1.82E+00 | 8.04E+01 | 2.41E-02 | 1.79E+00 | 1.84E+00 | 3.38E-02 | 1.24E-04 | 2.19E-01 | 3.37E-01 | 2.91E-02 | 1.65E-04 | 6.47E-02 | -3.03E-05 | 9.58E-07 | 3.57E-03 | 5.49E-03 | 9.03E-03 | 8.12E-05 | 6.92E-03 | 3.43E-03 |
| **PVDF** | **polyvinylfluoride production, US** | **1** | **kg** | 1.34E+01 | 1.68E+02 | 5.01E-01 | 1.28E+01 | 3.27E+00 | 3.56E-01 | 5.27E-03 | 4.37E+00 | 1.92E+00 | 3.13E-01 | 1.87E-03 | 1.06E+00 | -4.34E-04 | 6.86E-07 | 2.27E-02 | 2.23E-02 | 4.26E-02 | 5.90E-04 | 4.82E-02 | 1.42E-01 |
| **LiPF6** | **lithium hexafluorophosphate production, RoW** | **1** | **kg** | 1.89E+01 | 2.76E+02 | 1.49E+00 | 1.82E+01 | 5.53E+00 | 9.50E-01 | 7.41E-03 | 1.97E+01 | 2.27E+00 | 1.01E+00 | 1.06E-02 | 1.84E+00 | -1.37E-03 | 2.29E-06 | 5.28E-02 | 6.39E-02 | 1.51E-01 | 2.40E-02 | 1.81E-01 | 3.91E-01 |
| **EC** | **ethylene carbonate production, RoW** | **1** | **kg** | 1.40E+00 | 3.94E+01 | 7.58E-02 | 1.33E+00 | 8.45E-01 | 6.69E-02 | 4.62E-04 | 4.99E-01 | 1.81E-01 | 5.95E-02 | 2.45E-04 | 1.20E-01 | -5.37E-05 | 5.28E-08 | 1.54E-03 | 3.74E-03 | 4.04E-03 | 1.41E-04 | 5.63E-03 | 1.49E-02 |
| **DMC** | **dimethyl carbonate production, RoW** | **1** | **kg** | 2.16E+00 | 5.62E+01 | 9.32E-02 | 2.08E+00 | 1.31E+00 | 1.02E-01 | 6.89E-04 | 7.77E-01 | 1.75E-01 | 8.96E-02 | 3.68E-04 | 1.79E-01 | -1.09E-04 | 1.34E-07 | 3.35E-03 | 8.41E-03 | 7.17E-03 | 2.18E-04 | 1.32E-02 | 1.88E-02 |
| **Ethylene glycol** | **ethylene glycol production, RoW** | **1** | **kg** | 2.07E+00 | 5.43E+01 | 6.14E-02 | 2.01E+00 | 1.24E+00 | 6.57E-02 | 6.21E-04 | 6.08E-01 | 9.87E-02 | 5.83E-02 | 2.96E-04 | 1.09E-01 | -8.93E-05 | 3.72E-08 | 3.57E-03 | 6.37E-03 | 6.95E-03 | 6.93E-05 | 8.78E-03 | 1.87E-02 |
| **Deionized water** | **water production, deionised, RoW** | **1** | **kg** | 3.85E-04 | 5.67E-03 | 2.12E-05 | 4.51E-04 | 1.48E-04 | 2.57E-05 | 1.86E-07 | 2.26E-04 | 4.04E-05 | 2.29E-05 | 1.15E-07 | 1.50E-04 | -3.69E-08 | 2.47E-10 | 1.29E-06 | 1.53E-06 | 3.08E-06 | 6.76E-08 | 5.74E-06 | 3.15E-05 |
| **Graphite** | **graphite production, battery grade, RoW** | **1** | **kg** | 1.74E+00 | 4.29E+01 | 1.08E-01 | 1.41E+00 | 1.08E+00 | 3.92E-02 | 1.97E-03 | 1.26E+00 | 1.95E-01 | 3.65E-02 | 6.17E-04 | 1.71E-02 | -2.54E-04 | 6.48E-08 | 7.81E-03 | 1.76E-02 | 7.75E-03 | 6.14E-05 | 2.48E-02 | 9.63E-03 |
| **Printed wiring board** | **printed wiring board production, surface mounted, unspecified, Pb containing, GLO** | **1** | **kg** | 3.79E+02 | 5.72E+03 | 2.36E+01 | 3.69E+02 | 1.07E+02 | 1.48E+02 | 5.81E-01 | 7.82E+02 | 4.75E+01 | 1.33E+02 | 1.33E-01 | 3.22E+02 | -4.82E-02 | 3.69E-05 | 1.11E+00 | 1.47E+00 | 2.23E+00 | 8.55E-02 | 3.11E+00 | 3.19E+00 |
| **Printed wiring board mounting facility** | **printed wiring board mounting facility construction, GLO** | **1** | **unit** | 1.06E+07 | 1.68E+08 | 5.53E+06 | 9.53E+06 | 2.53E+06 | 6.57E+06 | 1.50E+04 | 3.11E+07 | 5.51E+05 | 5.79E+06 | 4.50E+03 | 1.11E+07 | -7.36E+03 | 6.40E-01 | 4.97E+04 | 5.44E+04 | 1.18E+05 | 4.24E+03 | 4.81E+05 | 7.61E+04 |
| **Chromium steel 18/8** | **steel production, chromium steel 18/8, hot rolled, RoW** | **1** | **kg** | 4.86E+00 | 6.66E+01 | 3.38E-01 | 4.60E+00 | 1.26E+00 | 4.40E-01 | 1.82E-03 | 2.80E+00 | 2.60E-01 | 4.41E-01 | 1.01E-03 | 1.91E+00 | -5.17E-04 | 2.02E-07 | 2.53E-02 | 1.89E-02 | 2.30E-02 | 7.56E-04 | 7.20E-02 | 2.60E-02 |
| **Sheet rolling, steel** | **sheet rolling, chromium steel, RoW** | **1** | **kg** | 6.75E-01 | 9.63E+00 | 3.81E-02 | 6.58E-01 | 1.89E-01 | 4.71E-02 | 2.72E-04 | 3.05E-01 | 5.45E-02 | 4.44E-02 | 1.42E-04 | 1.14E-01 | -5.24E-05 | 3.04E-08 | 2.43E-03 | 2.48E-03 | 3.03E-03 | 6.01E-05 | 6.04E-03 | 9.30E-03 |
| **Wire drawing, copper** | **wire drawing, copper, RoW** | **1** | **kg** | 8.36E-01 | 1.45E+01 | 5.29E-02 | 6.86E-01 | 1.92E-01 | 1.35E+00 | 2.78E-03 | 6.18E+00 | 5.02E-02 | 1.19E+00 | 4.69E-04 | 1.62E+00 | -1.25E-04 | 2.99E-08 | 6.74E-03 | 6.32E-03 | 1.88E-02 | 6.49E-04 | 1.32E-02 | 1.02E-02 |
| **Copper** | **copper production, primary, RoW** | **1** | **kg** | 4.73E+00 | 6.59E+01 | 4.18E-01 | 4.63E+00 | 1.40E+00 | 3.01E+01 | 5.79E-02 | 2.30E+02 | 1.61E-01 | 2.73E+01 | 7.44E-03 | 4.16E+01 | -1.95E-03 | 2.07E-07 | 1.54E-01 | 9.08E-02 | 5.17E-01 | 2.53E-02 | 2.27E-01 | 9.61E-02 |
| **Sheet rolling, copper** | **sheet rolling, copper, RoW** | **1** | **kg** | 6.57E-01 | 1.18E+01 | 4.67E-02 | 5.09E-01 | 1.39E-01 | 1.35E+00 | 2.70E-03 | 6.12E+00 | 3.22E-02 | 1.19E+00 | 4.34E-04 | 1.62E+00 | -1.14E-04 | 2.22E-08 | 6.28E-03 | 5.46E-03 | 1.81E-02 | 6.42E-04 | 1.23E-02 | 1.19E-02 |
| **Aluminum** | **aluminium production, primary, ingot, RoW** | **1** | **kg** | 1.96E+01 | 2.14E+02 | 4.66E-01 | 1.90E+01 | 4.49E+00 | 2.93E-01 | 6.44E-03 | 6.43E+00 | 4.05E-01 | 2.77E-01 | 3.52E-03 | 2.50E-01 | -1.69E-03 | 5.95E-07 | 4.66E-02 | 6.30E-02 | 9.73E-02 | 5.17E-04 | 1.19E-01 | 1.15E-01 |
| **Sheet rolling, aluminum** | **sheet rolling, aluminium, RoW** | **1** | **kg** | 6.57E-01 | 9.24E+00 | 2.09E-02 | 6.55E-01 | 1.97E-01 | 1.86E-02 | 2.52E-04 | 2.11E-01 | 4.80E-02 | 1.64E-02 | 1.33E-04 | 9.02E-03 | -4.23E-05 | 2.85E-08 | 1.56E-03 | 2.31E-03 | 2.57E-03 | 4.90E-05 | 3.29E-03 | 4.69E-03 |
| **PP** | **polypropylene production, granulate, RoW** | **1** | **kg** | 2.29E+00 | 8.11E+01 | 3.36E-02 | 2.21E+00 | 1.93E+00 | 4.06E-02 | 4.47E-04 | 4.18E-01 | 4.40E-02 | 3.61E-02 | 2.90E-04 | 6.88E-02 | -5.07E-05 | 2.89E-08 | 2.88E-03 | 7.40E-03 | 7.18E-03 | 6.33E-05 | 6.42E-03 | 1.91E-02 |
| **Plastic processing factory** | **plastic processing factory construction, RoW** | **1** | **unit** | 1.86E+08 | 3.02E+09 | 9.56E+07 | 1.66E+08 | 4.53E+07 | 1.27E+08 | 2.75E+05 | 5.97E+08 | 8.45E+06 | 1.12E+08 | 7.40E+04 | 1.98E+08 | -1.88E+05 | 1.24E+01 | 9.18E+05 | 1.05E+06 | 2.30E+06 | 7.65E+04 | 1.77E+07 | 1.26E+06 |
| **Injection moulding** | **injection moulding, RoW** | **1** | **kg** | 1.45E+00 | 2.46E+01 | 2.11E-01 | 1.43E+00 | 5.07E-01 | 2.24E-02 | 5.62E-04 | 4.38E-01 | 1.30E-01 | 1.98E-02 | 2.99E-04 | 2.54E-02 | -7.99E-05 | 9.97E-08 | 3.41E-03 | 4.32E-03 | 5.41E-03 | 6.86E-05 | 9.66E-03 | 1.17E-02 |
| **PET** | **polyethylene terephthalate production, granulate, amorphous, RoW** | **1** | **kg** | 3.13E+00 | 7.75E+01 | 9.18E-02 | 3.03E+00 | 1.82E+00 | 9.35E-02 | 7.04E-04 | 7.97E-01 | 1.38E-01 | 8.35E-02 | 5.37E-04 | 2.08E-01 | -1.22E-04 | 1.31E-07 | 4.77E-03 | 9.61E-03 | 1.06E-02 | 2.42E-04 | 1.80E-02 | 3.32E-02 |
| **PE** | **polyethylene production, high density, granulate, RoW** | **1** | **kg** | 2.33E+00 | 7.97E+01 | 3.70E-02 | 2.25E+00 | 1.89E+00 | 4.21E-02 | 4.62E-04 | 4.35E-01 | 5.61E-02 | 3.74E-02 | 3.00E-04 | 7.00E-02 | -5.66E-05 | 4.28E-08 | 3.09E-03 | 8.03E-03 | 7.35E-03 | 7.39E-05 | 6.70E-03 | 2.19E-02 |
| **Metal working, copper** | **metal working, average for copper product manufacturing, RoW** | **1** | **kg** | 3.57E+00 | 6.58E+01 | 2.16E-01 | 2.74E+00 | 7.56E-01 | 7.73E+00 | 1.54E-02 | 3.50E+01 | 1.94E-01 | 6.81E+00 | 2.72E-03 | 9.35E+00 | -8.73E-04 | 1.60E-07 | 3.52E-02 | 2.76E-02 | 1.02E-01 | 3.78E-03 | 9.21E-02 | 3.33E-02 |
| **Steel** | **steel production, low-alloyed, hot rolled, RoW** | **1** | **kg** | 2.12E+00 | 2.29E+01 | 7.05E-02 | 1.82E+00 | 5.18E-01 | 2.54E-01 | 1.27E-03 | 1.73E+00 | 1.06E-01 | 2.26E-01 | 5.88E-04 | 1.31E+00 | -1.66E-04 | 7.98E-08 | 7.84E-03 | 9.19E-03 | 7.52E-03 | 2.81E-04 | 2.31E-02 | 2.20E-02 |
| **Metal working, steel** | **metal working, average for steel product manufacturing, RoW** | **1** | **kg** | 2.21E+00 | 2.79E+01 | 8.54E-02 | 2.10E+00 | 5.98E-01 | 1.23E-01 | 8.61E-04 | 9.10E-01 | 1.48E-01 | 1.09E-01 | 9.81E-04 | 3.74E-01 | -3.53E-04 | 1.23E-07 | 5.40E-03 | 6.28E-03 | 7.36E-03 | 7.54E-04 | 3.61E-02 | 1.60E-02 |
| **Glass fiber** | **glass fibre production, RoW** | **1** | **kg** | 2.50E+00 | 3.54E+01 | 8.16E-02 | 2.46E+00 | 8.06E-01 | 5.65E-02 | 7.12E-04 | 2.25E+00 | 1.46E-01 | 5.02E-02 | 6.49E-04 | 4.58E-01 | -1.32E-04 | 1.23E-07 | 7.14E-03 | 1.17E-02 | 1.49E-02 | 2.97E-04 | 1.36E-02 | 1.68E-02 |
| **Transport, freight, lorry 16-32 metric ton, EURO3** | **transport, freight, lorry 16-32 metric ton, EURO3, RoW** | **1** | **tkm** | 1.74E-01 | 2.76E+00 | 1.58E-03 | 1.71E-01 | 6.24E-02 | 1.69E-03 | 1.47E-05 | 5.08E-02 | 1.20E-02 | 1.98E-03 | 4.95E-05 | 8.22E-03 | -1.31E-05 | 3.01E-08 | 4.54E-04 | 1.36E-03 | 8.95E-04 | 7.64E-05 | 8.85E-03 | 2.56E-04 |
| **Transport, freight, sea, container ship** | **transport, freight, sea, container ship, GLO** | **1** | **tkm** | 9.43E-03 | 1.28E-01 | 5.07E-05 | 9.34E-03 | 2.92E-03 | 6.43E-05 | 3.73E-07 | 6.14E-04 | 5.45E-04 | 6.44E-05 | 7.76E-06 | 2.42E-04 | -7.37E-08 | 1.50E-09 | 7.85E-05 | 2.17E-04 | 2.33E-04 | 4.80E-07 | 1.64E-05 | 7.95E-06 |
| **Transport, freight train, diesel** | **transport, freight train, diesel, US** | **1** | **tkm** | 5.81E-02 | 8.06E-01 | 1.33E-03 | 5.62E-02 | 1.78E-02 | 1.03E-03 | 8.18E-06 | 9.94E-03 | 3.81E-03 | 9.28E-04 | 2.80E-05 | 3.07E-03 | -5.91E-06 | 7.98E-09 | 2.17E-04 | 7.62E-04 | 4.64E-04 | 4.02E-06 | 2.41E-03 | 1.32E-04 |
| **Transport, freight, lorry >32 metric ton, EURO4** | **transport, freight, lorry >32 metric ton, EURO4, RoW** | **1** | **tkm** | 9.33E-02 | 1.53E+00 | 8.40E-04 | 9.20E-02 | 3.46E-02 | 7.35E-04 | 7.64E-06 | 3.01E-02 | 6.81E-03 | 1.02E-03 | 1.83E-05 | 3.12E-03 | -1.22E-05 | 1.69E-08 | 2.13E-04 | 5.24E-04 | 3.63E-04 | 5.60E-05 | 8.96E-03 | 1.46E-04 |

| | | | | | | | | | | | | | | | | | | | | | | | |
|---|---|---|---|---|---|---|---|---|---|---|---|---|---|---|---|---|---|---|---|---|---|---|---|
| **Treatment of scrap steel, inert material landfill** | **treatment of scrap steel, inert material landfill, RoW** | **1** | **kg** | 5.29E-03 | 1.58E-01 | 5.02E-04 | 5.14E-03 | 3.57E-03 | 5.33E-05 | 5.45E-07 | 7.38E-04 | 6.58E-04 | 4.90E-05 | 1.89E-06 | 1.65E-04 | -2.55E-06 | 1.72E-09 | 1.79E-05 | 5.51E-05 | 3.80E-05 | 5.64E-07 | 8.10E-04 | 1.06E-05 |
| **Treatment of aluminum scrap, post-consumer, by collecting, sorting, cleaning, pressing,** | **treatment of aluminium scrap, post-consumer, by collecting, sorting, cleaning, pressing, RoW** | **1** | **kg** | 7.16E-01 | 8.87E+00 | 1.99E-02 | 3.09E-01 | 5.19E-02 | 9.40E+00 | 9.99E-05 | 2.47E-01 | 1.08E-02 | 8.04E+00 | 4.52E-05 | 4.28E-02 | -5.91E-05 | 1.35E-08 | 5.81E-04 | 7.97E-04 | 1.07E-03 | 4.33E-05 | 7.75E-03 | 9.22E-04 |
| **Treatment of aluminum scrap, post-consumer, prepared for recycling, at refiner** | **treatment of aluminium scrap, post-consumer, prepared for recycling, at refiner, RoW** | **1** | **kg** | 5.57E-01 | 7.88E+00 | 6.64E-02 | 8.74E-01 | 2.19E-01 | 9.75E+00 | 4.32E-04 | 1.05E+00 | 7.01E-02 | 8.35E+00 | 1.76E-04 | 5.55E+00 | -2.12E-04 | 3.52E-07 | 1.69E-03 | 2.75E-03 | 3.69E-03 | 2.36E-04 | 2.20E-02 | 8.01E-03 |
| **Treatment of copper scrap by electrolytic refining** | **treatment of copper scrap by electrolytic refining, RoW** | **1** | **kg** | 1.79E+00 | 2.21E+01 | 2.02E-01 | 2.33E+00 | 5.94E-01 | 3.52E+00 | 7.40E-03 | 1.47E+01 | 1.25E-01 | 3.09E+00 | 1.25E-03 | 5.41E+00 | -4.06E-04 | 7.71E-08 | 1.39E-02 | 1.35E-02 | 2.41E-02 | 1.36E-03 | 4.52E-02 | 1.77E-02 |
| **Treatment of waste glass, sanitary landfill** | **treatment of waste glass, sanitary landfill, GLO** | **1** | **kg** | 9.33E-03 | 2.49E-01 | 1.05E-03 | 9.14E-03 | 5.64E-03 | 1.09E-03 | 1.08E-06 | 1.01E-02 | 1.01E-03 | 9.41E-04 | 3.50E-06 | 3.79E-04 | -3.97E-06 | 2.55E-09 | 3.15E-05 | 9.33E-05 | 6.42E-05 | 1.09E-06 | 3.13E-03 | 2.38E-05 |
| **Treatment of scrap printed wiring boards, shredding, and separation** | **treatment of scrap printed wiring boards, shredding and separation, RoW** | **1** | **kg** | 6.13E-01 | 4.92E+00 | 1.24E-03 | 2.86E-02 | 8.31E-03 | 4.69E-04 | 1.46E-05 | 1.06E-02 | 4.28E-03 | 4.21E-04 | 6.16E-06 | 4.84E-04 | -1.83E-06 | 1.26E-09 | 7.36E-05 | 7.52E-05 | 1.13E-04 | 1.04E-06 | 1.49E-04 | 1.65E-04 |
| **Treatment of spent antifreeze liquid, hazardous waste incineration** | **treatment of spent antifreezer liquid, hazardous waste incineration, RoW** | **1** | **kg** | 4.23E+00 | 2.50E+01 | 1.60E-01 | 3.42E+00 | 5.38E-01 | 2.05E-02 | 9.13E-04 | 8.63E-01 | 2.57E-02 | 1.96E-02 | 3.10E-04 | 9.91E-03 | -2.11E-04 | 5.31E-08 | 3.85E-03 | 1.32E-02 | 1.15E-02 | 1.02E-04 | 1.81E-02 | 3.23E-03 |
| **Treatment of waste reinforcement steel, recycling** | **treatment of waste reinforcement steel, recycling, RoW** | **1** | **kg** | 5.77E-02 | 8.40E-01 | 1.98E-04 | 5.66E-02 | 1.92E-02 | 2.36E-04 | 2.08E-06 | 2.76E-03 | 3.58E-03 | 2.17E-04 | 2.72E-05 | 8.73E-04 | -5.07E-07 | 9.78E-09 | 2.36E-04 | 7.98E-04 | 4.55E-04 | 2.44E-06 | 1.06E-04 | 4.68E-05 |
| **Treatment of waste polypropylene, sanitary landfill** | **treatment of waste polypropylene, sanitary landfill, RoW** | **1** | **kg** | 1.08E-01 | 2.71E-01 | 1.11E-03 | 9.51E-02 | 6.06E-03 | 5.96E-02 | 1.75E-06 | 3.51E-01 | 1.19E-03 | 5.78E-02 | 1.09E-03 | 4.20E-04 | -4.08E-06 | 2.61E-09 | 3.54E-05 | 1.25E-04 | 7.00E-05 | 6.02E-06 | 3.14E-03 | 3.38E-05 |
| **Treatment of waste polyethylene, sanitary landfill** | **treatment of waste polyethylene, sanitary landfill, RoW** | **1** | **kg** | 1.26E-01 | 2.71E-01 | 1.11E-03 | 1.11E-01 | 6.07E-03 | 7.06E-02 | 1.77E-06 | 4.16E-01 | 1.20E-03 | 6.85E-02 | 1.30E-03 | 4.20E-04 | -4.09E-06 | 2.61E-09 | 3.56E-05 | 1.30E-04 | 7.03E-05 | 6.91E-06 | 3.14E-03 | 3.41E-05 |
| **Treatment of waste polyethylene terephthalate, sanitary landfill** | **treatment of waste polyethylene terephthalate, sanitary landfill, RoW** | **1** | **kg** | 8.90E-02 | 2.78E-01 | 1.13E-03 | 7.87E-02 | 6.19E-03 | 4.23E-02 | 1.98E-06 | 2.24E-01 | 1.26E-03 | 3.96E-02 | 6.44E-03 | 4.28E-04 | -4.11E-06 | 2.63E-09 | 3.67E-05 | 1.22E-04 | 7.24E-05 | 4.19E-05 | 3.14E-03 | 3.66E-05 |
| **Treatment of waste PVDF, municipal incineration** | **treatment of waste polypropylene, sanitary landfill, RoW** | **1** | **kg** | 2.29E+00 | 1.64E+00 | 7.66E-03 | 2.29E+00 | 3.55E-02 | 2.67E-02 | 4.52E-05 | 8.91E-01 | 7.67E-03 | 2.42E-02 | 5.61E-05 | 1.37E-02 | -1.29E-05 | 1.93E-08 | 3.11E-04 | 8.29E-04 | 6.77E-04 | 1.01E-04 | 4.78E-03 | 2.25E-03 |
| **Citric acid** | **citric acid production, RoW** | **1** | **kg** | 6.31E+00 | 1.03E+02 | 1.61E+00 | 6.20E+00 | 1.46E+00 | 2.38E-01 | 2.35E-03 | 2.79E+00 | 2.32E-01 | 2.07E-01 | 9.57E-03 | 1.04E+00 | -3.71E-04 | 6.74E-07 | 1.66E-02 | 1.90E-02 | 5.36E-02 | 2.01E-02 | 5.20E-02 | 7.76E-02 |
| **$H_2O_2$** | **hydrogen peroxide production, product in 50% solution state, RoW** | **1** | **kg** | 1.47E+00 | 2.26E+01 | 4.50E-02 | 1.42E+00 | 5.19E-01 | 5.78E-02 | 4.30E-04 | 4.73E-01 | 7.69E-02 | 5.11E-02 | 4.42E-04 | 7.42E-02 | -6.76E-05 | 1.00E-07 | 2.68E-03 | 4.01E-03 | 4.93E-03 | 4.44E-04 | 6.59E-03 | 7.43E-02 |
| **$Na_2CO_3$** | **soda production, solvay process, RoW** | **1** | **kg** | 4.76E-01 | 5.51E+00 | 8.79E-02 | 4.62E-01 | 1.04E-01 | 4.07E-02 | 2.39E-04 | 3.15E-01 | 2.02E-02 | 3.63E-02 | 2.33E-04 | 2.01E-01 | -4.49E-05 | 1.41E-08 | 1.44E-03 | 1.55E-03 | 4.78E-03 | 5.00E-05 | 5.24E-03 | 1.70E-02 |
| **$Mn_2O_3$** | **manganese (III) oxide production, RoW** | **1** | **kg** | 1.92E+00 | 3.10E+01 | 1.48E-01 | 1.90E+00 | 4.58E-01 | 6.12E-02 | 1.02E-03 | 8.07E-01 | 5.33E-01 | 5.54E-02 | 4.01E-04 | 8.05E+01 | -2.82E-04 | 1.59E-07 | 2.42E-03 | 4.50E-03 | 6.23E-03 | 7.95E-05 | 3.65E-02 | 4.51E-02 |
| **Treatment of sludge from steel rolling, residual material landfill** | **treatment of sludge from steel rolling, residual material landfill, RoW** | **1** | **kg** | 3.31E-01 | 2.25E+00 | 1.15E-02 | 3.28E-01 | 4.88E-02 | 1.90E-01 | 9.36E-05 | 3.47E-01 | 9.94E-03 | 1.84E-01 | 4.11E-05 | 3.24E-02 | -2.67E-05 | 1.60E-08 | 3.80E-04 | 9.14E-04 | 8.27E-04 | 1.96E-05 | 1.05E-02 | 5.78E-04 |
| **Limestone** | **limestone quarry operation, RoW** | **1** | **kg** | 2.16E-03 | 2.86E-02 | 1.23E-04 | 2.11E-03 | 6.44E-04 | 3.30E-05 | 1.83E-07 | 2.58E-04 | 1.20E-04 | 2.94E-05 | 2.33E-06 | 7.93E-05 | -4.52E-08 | 3.05E-10 | 6.32E-05 | 5.50E-05 | 4.10E-05 | 4.01E-07 | 8.81E-06 | 2.64E-05 |
| **Limestone quarry construction** | **limestone quarry construction, RoW** | **1** | **unit** | 3.26E+05 | 4.83E+06 | 2.04E+05 | 3.17E+05 | 8.11E+04 | 1.50E+05 | 3.49E+02 | 7.32E+05 | 1.56E+04 | 1.31E+05 | 1.78E+02 | 3.88E+05 | -3.91E+01 | 2.16E-02 | 1.51E+03 | 1.74E+03 | 3.71E+03 | 1.33E+02 | 5.60E+04 | 2.31E+03 |
| **Sand quarry operation** | **sand quarry operation, extraction from river bed, RoW** | **1** | **kg** | 4.76E-03 | 6.99E-02 | 1.55E-02 | 4.68E-03 | 1.58E-03 | 2.73E-05 | 2.63E-07 | 3.11E-04 | 3.16E-04 | 2.47E-05 | 2.22E-06 | 1.48E-04 | -5.20E-08 | 7.92E-10 | 1.93E-05 | 6.42E-05 | 3.70E-05 | 2.17E-07 | 9.92E-06 | 1.55E-04 |
| **Silica sand** | **silica sand production, RoW** | **1** | **kg** | 4.25E-02 | 5.08E-01 | 1.52E-02 | 4.18E-02 | 1.11E-02 | 3.42E-04 | 8.32E-06 | 1.13E-02 | 1.44E-03 | 3.48E-04 | 9.77E-06 | 9.33E-04 | -2.03E-05 | 3.43E-09 | 1.07E-04 | 2.42E-04 | 2.58E-04 | 6.22E-06 | 9.67E-04 | 2.06E-04 |
| **Ground granulated blast furnace slag** | **ground granulated blast furnace slag production, US** | **1** | **kg** | 8.28E-02 | 1.48E+00 | 4.42E-03 | 8.15E-02 | 2.97E-02 | 3.53E-03 | 5.01E-05 | 4.05E-02 | 1.77E-02 | 2.98E-03 | 1.60E-05 | 4.80E-03 | -7.33E-06 | 7.77E-09 | 2.41E-04 | 1.67E-04 | 4.48E-04 | 7.91E-06 | 8.43E-04 | 4.53E-04 |
| **Coke** | **coking, RoW** | **1** | **MJ** | 2.53E-02 | 1.00E+00 | 1.63E-03 | 2.13E-02 | 2.25E-02 | 6.59E-04 | 3.84E-05 | 2.42E-02 | 4.36E-04 | 6.25E-04 | 1.16E-05 | 2.28E-04 | -5.48E-06 | 4.37E-10 | 1.57E-04 | 4.06E-04 | 1.38E-04 | 8.30E-07 | 5.50E-04 | 9.32E-05 |
| **Treatment of waste polyethylene terephthalate, municipal incineration** | **treatment of waste polyethylene terephthalate, municipal incineration, RoW** | **1** | **kg** | 2.06E+00 | 2.96E-01 | 3.40E-04 | 2.07E+00 | 7.79E-03 | 6.56E-02 | 2.81E-06 | 4.83E-01 | 5.12E-04 | 6.47E-02 | 9.27E-05 | 1.16E-03 | -1.37E-06 | 2.05E-09 | 1.29E-04 | 5.09E-04 | 3.06E-04 | 2.18E-04 | 2.06E-04 | 1.55E-03 |
| **Treatment of waste polypropylene, municipal incineration** | **treatment of waste polypropylene, municipal incineration, RoW** | **1** | **kg** | 2.55E+00 | 2.64E-01 | 4.33E-04 | 3.02E+00 | 7.86E-03 | 1.06E-01 | 3.42E-06 | 6.78E-01 | 6.12E-04 | 1.07E-01 | 3.43E-05 | 1.34E-03 | -1.54E-06 | 2.27E-09 | 1.24E-04 | 4.85E-04 | 2.99E-04 | 3.02E-04 | 2.75E-04 | 2.03E-03 |
| **Treatment of waste polyethylene, municipal incineration** | **treatment of waste polyethylene, municipal incineration, RoW** | **1** | **kg** | 3.02E+00 | 3.04E-01 | 3.84E-04 | 2.55E+00 | 6.79E-03 | 8.93E-02 | 3.09E-06 | 5.72E-01 | 5.45E-04 | 9.03E-02 | 2.91E-05 | 1.24E-03 | -1.45E-06 | 1.96E-09 | 1.07E-04 | 4.13E-04 | 2.55E-04 | 2.55E-04 | 2.45E-04 | 2.02E-03 |
| **HCl** | **hydrochloric acid production, from the reaction of hydrogen with chlorine, RoW** | **1** | **kg** | 1.47E+00 | 2.21E+01 | 7.91E-02 | 1.45E+00 | 4.44E-01 | 7.50E-02 | 7.31E-04 | 1.12E+00 | 1.45E-01 | 7.34E-02 | 3.66E-04 | 1.27E-01 | -1.11E-04 | 5.88E-07 | 3.93E-03 | 4.30E-03 | 6.35E-03 | 9.36E-04 | 1.22E-02 | 2.71E-02 |

| | | | | | | | | | | | | | | | | | | | | | | | |
|---|---|---|---|---|---|---|---|---|---|---|---|---|---|---|---|---|---|---|---|---|---|---|---|
| **Fe** | **pig iron production, RoW** | **1** | **kg** | 1.75E+00 | 1.81E+01 | 3.21E-02 | 1.58E+00 | 4.04E-01 | 1.34E-02 | 6.42E-04 | 5.21E-01 | 2.42E-02 | 1.27E-02 | 3.96E-04 | 1.20E-02 | -1.12E-04 | 3.01E-08 | 7.22E-03 | 8.86E-03 | 5.67E-03 | 5.21E-05 | 1.25E-02 | 4.15E-03 |
| **$NaClO_3$** | **sodium chlorate production, powder, RoW** | **1** | **kg** | 4.66E+00 | 6.43E+01 | 1.77E-01 | 4.57E+00 | 1.27E+00 | 1.29E-01 | 2.15E-03 | 1.78E+00 | 4.57E-01 | 1.15E-01 | 1.23E-03 | 1.31E-01 | -2.62E-04 | 1.82E-07 | 1.23E-02 | 1.25E-02 | 1.82E-02 | 6.06E-04 | 2.57E-02 | 1.17E-01 |
| **$Na_2CO_3$** | **soda production, solvay process, RoW** | **1** | **kg** | 4.76E-01 | 5.51E+00 | 8.79E-02 | 4.62E-01 | 1.04E-01 | 4.07E-02 | 2.39E-04 | 3.15E-01 | 2.02E-02 | 3.63E-02 | 2.33E-04 | 2.01E-01 | -4.49E-05 | 1.41E-08 | 1.44E-03 | 1.55E-03 | 4.78E-03 | 5.00E-05 | 5.24E-03 | 1.70E-02 |
| **Granulated blast furnace slag** | **granulated blast furnace slag production, RoW** | **1** | **kg** | 4.85E-03 | 7.64E-02 | 1.14E-04 | 4.78E-03 | 1.60E-03 | 6.93E-05 | 1.29E-06 | 1.63E-03 | 5.08E-04 | 7.01E-05 | 1.22E-06 | 1.88E-04 | -4.34E-07 | 6.62E-10 | 2.09E-05 | 2.69E-05 | 2.18E-05 | 1.39E-06 | 2.18E-04 | 4.97E-04 |
| **Iron mine operation** | **iron mine operation, crude ore, 63% Fe, IN** | **1** | **kg** | 2.38E-02 | 3.50E-01 | 1.47E-03 | 2.35E-02 | 7.89E-03 | 1.84E-04 | 2.61E-06 | 2.63E-03 | 1.39E-03 | 1.69E-04 | 1.57E-05 | 4.64E-04 | 1.77E-05 | 3.56E-09 | 1.55E-03 | 3.72E-04 | 2.48E-04 | 2.13E-06 | 2.12E-04 | 5.94E-05 |
| **HF** | **hydrogen fluoride production, RoW** | **1** | **kg** | 1.29E+00 | 2.50E+01 | 1.18E-01 | 1.27E+00 | 5.86E-01 | 1.93E-01 | 6.98E-04 | 1.21E+00 | 9.45E-02 | 1.70E-01 | 3.51E-04 | 1.69E+00 | -1.47E-04 | 9.66E-08 | 7.02E-03 | 8.03E-03 | 2.30E-02 | 2.88E-04 | 2.09E-02 | 5.14E-02 |
| **Glucose** | **glucose production, GLO** | **1** | **kg** | 1.22E+00 | 3.55E+01 | 7.19E-01 | 1.22E+00 | 3.75E-01 | 5.95E-02 | 4.54E-04 | 4.01E-01 | 1.01E-01 | 4.58E-02 | 4.70E-03 | 1.69E-01 | -9.21E-05 | 7.87E-08 | 3.38E-03 | 3.44E-03 | 1.26E-02 | 1.21E-02 | 9.12E-02 | 2.81E-02 |
| **electricity, low voltage, US-NPCC** | **market for electricity, low voltage, US-NPCC** | 1 | kWh | 2.70E-01 | 4.26E-01 | 3.90E+00 | 5.07E-04 | 5.22E+00 | 2.01E-03 | 3.91E-02 | 7.26E-01 | 1.60E-01 | 1.05E+01 | 6.20E-02 | 2.65E-01 | 1.10E-01 | 2.37E-02 | 3.79E-05 | 4.90E-02 | 3.64E-01 | 1.95E-02 | 6.96E-05 | 1.26E-02 |
| **electricity, low voltage, US-WECC** | **market for electricity, low voltage, US-WECC** | 1 | kWh | 4.14E-01 | 1.69E-01 | 5.23E+00 | 1.58E-01 | 1.14E+00 | 2.08E-04 | 3.29E-01 | 1.02E+00 | 2.98E-01 | 8.34E+00 | 2.63E-02 | 4.10E-01 | 1.34E-01 | 2.92E-02 | 4.35E-04 | 2.80E-01 | 7.43E-02 | 2.51E-02 | 1.23E-04 | 1.14E-02 |
| **electricity, low voltage, US-TRE** | **market for electricity, low voltage, US-TRE** | 1 | kWh | 5.57E-01 | 3.75E-02 | 7.08E+00 | 9.39E-04 | 1.49E+00 | 6.52E-05 | 5.03E-02 | 2.85E-02 | 7.18E-01 | 9.41E+00 | 6.33E-03 | 5.52E-01 | 1.86E-01 | 3.36E-02 | 7.01E-04 | 4.35E-01 | 9.70E-02 | 2.88E-02 | 1.70E-04 | 1.10E-02 |
| **electricity, low voltage, US-SERC** | **market for electricity, low voltage, US-SERC** | 1 | kWh | 6.19E-01 | 4.12E-01 | 7.64E+00 | 4.67E-04 | 3.71E+00 | 1.94E-04 | 5.90E-02 | 1.75E-01 | 2.11E-02 | 1.20E+01 | 5.89E-02 | 6.13E-01 | 1.95E-01 | 2.94E-02 | 4.41E-04 | 2.97E-01 | 2.41E-01 | 2.50E-02 | 1.37E-04 | 1.15E-02 |
| **electricity, low voltage, US-RFC** | **market for electricity, low voltage, US-RFC** | 1 | kWh | 6.12E-01 | 9.41E-02 | 6.78E+00 | 4.78E-04 | 4.37E+00 | 5.32E-04 | 1.59E-02 | 7.24E-02 | 1.28E-01 | 1.15E+01 | 1.62E-02 | 6.06E-01 | 1.69E-01 | 2.80E-02 | 3.53E-04 | 2.47E-01 | 2.84E-01 | 2.39E-02 | 1.28E-04 | 1.31E-02 |
| **electricity, low voltage, US-MRO** | **market for electricity, low voltage, US-MRO** | 1 | kWh | 6.94E-01 | 1.83E-01 | 7.60E+00 | 5.62E-04 | 1.11E+00 | 2.23E-04 | 2.31E-02 | 3.25E-01 | 9.61E-01 | 1.02E+01 | 2.92E-02 | 6.89E-01 | 1.84E-01 | 3.88E-02 | 1.06E-03 | 6.71E-01 | 7.23E-02 | 3.45E-02 | 2.67E-04 | 1.16E-02 |
| **electricity, low voltage, US-HICC** | **market for electricity, low voltage, US-HICC** | 1 | kWh | 9.68E-01 | 1.12E-01 | 1.17E+01 | 9.19E-02 | 3.10E-01 | 4.55E-03 | 2.42E-02 | 8.76E-02 | 2.79E-01 | 1.26E+01 | 2.04E-02 | 9.64E-01 | 2.69E-01 | 2.99E-02 | 4.20E-04 | 3.43E-01 | 5.24E-02 | 2.71E-02 | 3.29E-04 | 1.47E-02 |
| **electricity, low voltage, US-FRCC** | **market for electricity, low voltage, US-FRCC** | 1 | kWh | 5.41E-01 | 2.86E-01 | 7.43E+00 | 5.30E-04 | 1.94E+00 | 1.44E-03 | 6.06E-02 | 2.47E-02 | 5.12E-03 | 9.75E+00 | 4.07E-02 | 5.33E-01 | 2.03E-01 | 2.52E-02 | 9.77E-05 | 8.50E-02 | 1.26E-01 | 2.03E-02 | 7.62E-05 | 1.17E-02 |
| **electricity, low voltage, US-ASCC** | **market for electricity, low voltage, US-ASCC** | 1 | kWh | 5.67E-01 | 1.35E-02 | 7.74E+00 | 2.81E-04 | 4.26E-02 | 4.80E-05 | 1.95E-04 | 1.14E+00 | 8.45E-02 | 9.02E+00 | 6.33E-03 | 5.61E-01 | 2.03E-01 | 2.84E-02 | 3.40E-04 | 2.48E-01 | 9.48E-03 | 2.38E-02 | 1.28E-04 | 1.02E-02 |
| **electricity, low voltage, US-SPP** | **market for electricity, low voltage, US-NPCC** | 1 | kWh | 6.81E-01 | 2.03E-01 | 7.58E+00 | 1.04E-03 | 1.40E+00 | 2.16E-04 | 2.86E-02 | 3.03E-01 | 8.52E-01 | 1.04E+01 | 3.17E-02 | 6.77E-01 | 1.85E-01 | 3.77E-02 | 9.81E-04 | 6.24E-01 | 9.09E-02 | 3.33E-02 | 2.50E-04 | 1.16E-02 |

## REFERENCES AND NOTES


1. Department of Energy, *Vehicle Technologies Office's Research Plan to Reduce, Recycle, and Recover Critical Materials in Lithium-Ion Batteries* (U.S. Department of Energy, 2019).

2. G. Harper, R. Sommerville, E. Kendrick, L. Driscoll, P. Slater, R. Stolkin, A. Walton, P. Christensen, O. Heidrich, S. Lambert, A. Abbott, K. Ryder, L. Gaines, P. Anderson, Recycling lithium-ion batteries from electric vehicles. *Nature* **575**, 75–86 (2019).

3. W. Li, E. M. Erickson, A. Manthiram, High-nickel layered oxide cathodes for lithium-based automotive batteries. *Nat. Energy* **5**, 26–34 (2020).

4. International Energy Agency, *Global EV Outlook 2019* (International Energy Agency, 2019).

5. T. P. Narins, The battery business: Lithium availability and the growth of the global electric car industry. *Extr. Ind. Soc.* **4**, 321–328 (2017).

6. L. A. Gil-Alana, M. Monge, Lithium: Production and estimated consumption. Evidence of persistence. *Resour. Policy* **60**, 198–202 (2019).

7. S. van den Brink, R. Kleijn, B. Sprecher, A. Tukker, Identifying supply risks by mapping the cobalt supply chain. *Resour. Conserv. Recycl.* **156**, 104743 (2020).

8. E. A. Olivetti, G. Ceder, G. G. Gaustad, X. Fu, Lithium-ion battery supply chain considerations: Analysis of potential bottlenecks in critical metals. *Joule* **1**, 229–243 (2017).

9. The White House, *Building Resilient Supply Chains, Revitalizing American Manufacturing, and Fostering Broad-Based Growth* (The White House, 2021).

10. X. Wang, G. Gaustad, C. W. Babbitt, K. Richa, Economies of scale for future lithium-ion battery recycling infrastructure. *Resour. Conserv. Recycl.* **83**, 53–62 (2014).

11. A. Manthiram, A reflection on lithium-ion battery cathode chemistry. *Nat. Commun.* **11**, 1550 (2020).

12. D. Kamath, R. Arsenault, H. C. Kim, A. Anctil, Economic and environmental feasibility of second-life lithium-ion batteries as fast-charging energy storage. *Environ. Sci. Technol.* **54**, 6878–6887 (2020).

13. D. Kamath, S. Shukla, R. Arsenault, H. C. Kim, A. Anctil, Evaluating the cost and carbon footprint of second-life electric vehicle batteries in residential and utility-level applications. *Waste Manag.* **113**, 497–507 (2020).

14. S. Bobba, F. Mathieux, G. A. Blengini, How will second-use of batteries affect stocks and flows in the EU? A model for traction Li-ion batteries *Resour. Conserv. Recycl.* **145**, 279–291 (2019).

15. M. Bielewski, D. Blagoeva, M. Cordella, F. Di Persio, P. Gaudillat, S. Hildebrand, L. Mancini, F. Mathieux, P. Moretto, E. Paffumi, D. Paraskevas, V. Ruiz Ruiz, J. V. Sanfelix Forner, A. Villanueva Krzyzaniak, L. Zampori, *Analysis of Sustainability Criteria for Lithium-Ion Batteries Including Related Standards and Regulations* (Publications Office of the European Union, 2021).

16. J. Neubauer, A. Pesaran, The ability of battery second use strategies to impact plug-in electric vehicle prices and serve utility energy storage applications. *J. Power Sources* **196**, 10351–10358 (2011).

17. W. Lih, J. Yen, F. Shieh, Y. Liao, Second Use of Retired Lithium-Ion Battery Packs from Electric Vehicles: Technological Challenges, Cost Analysis and Optimal Business Model, in *2012 International Symposium on Computer, Consumer and Control* (2012), pp. 381–384.

18. R. Faria, P. Marques, R. Garcia, P. Moura, F. Freire, J. Delgado, A. T. de Almeida, Primary and secondary use of electric mobility batteries from a life cycle perspective. *J. Power Sources* **262**, 169–177 (2014).

19. L. Ahmadi, M. Fowler, S. B. Young, R. A. Fraser, B. Gaffney, S. B. Walker, Energy efficiency of Li-ion battery packs re-used in stationary power applications. *Sustain. Energy Technol. Assess.* **8**, 9–17 (2014).

20. L. Ahmadi, S. B. Young, M. Fowler, R. A. Fraser, M. A. Achachlouei, A cascaded life cycle: Reuse of electric vehicle lithium-ion battery packs in energy storage systems. *Int. J. Life Cycle Assess.* **22**, 111–124 (2017).

21. K. Richa, C. W. Babbitt, N. G. Nenadic, G. Gaustad, Environmental tradeoffs across cascading lithium-ion battery life cycles. *Int. J. Life Cycle Assess.* **22**, 66–81 (2017).

22. L. C. Casals, B. Amante García, C. Canal, Second life batteries lifespan: Rest of useful life and environmental analysis. *J. Environ. Manage.* **232**, 354–363 (2019).

23. E. Hossain, D. Murtaugh, J. Mody, H. M. R. Faruque, M. S. H. Sunny, N. Mohammad, A comprehensive review on second-life batteries: Current state, manufacturing considerations, applications, impacts, barriers & potential solutions, business strategies, and policies. *IEEE Access* **7**, 73215–73252 (2019).

24. L. C. Casals, B. A. García, F. Aguesse, A. Iturrondobeitia, Second life of electric vehicle batteries: Relation between materials degradation and environmental impact. *Int. J. Life Cycle Assess.* **22**, 82–93 (2017).

25. M. Hiremath, K. Derendorf, T. Vogt, Comparative life cycle assessment of battery storage systems for stationary applications. *Environ. Sci. Technol.* **49**, 4825–4833 (2015).

26. R. Sommerville, P. Zhu, M. A. Rajaeifar, O. Heidrich, V. Goodship, E. Kendrick, A qualitative assessment of lithium ion battery recycling processes. *Resour. Conserv. Recycl.* **165**, 105219 (2021).

27. D. A. Notter, M. Gauch, R. Widmer, P. Wäger, A. Stamp, R. Zah, H.-J. Althaus, Contribution of Li-ion batteries to the environmental impact of electric vehicles. *Environ. Sci. Technol.* **44**, 7744–7744 (2010).

28. H. C. Kim, T. J. Wallington, R. Arsenault, C. Bae, S. Ahn, J. Lee, Cradle-to-gate emissions from a commercial electric vehicle Li-ion battery: A comparative analysis. *Environ. Sci. Technol.* **50**, 7715–7722 (2016).

29. G. Majeau-Bettez, T. R. Hawkins, A. H. Strømman, Life cycle environmental assessment of lithium-ion and nickel metal hydride batteries for plug-in hybrid and battery electric vehicles. *Environ. Sci. Technol.* **45**, 4548–4554 (2011).

30. Environmental Protection Agency, *Application of LCA to Nanoscale Technology: Li-ion Batteries for Electric Vehicles* (United States Environmental Protection Agency, 2013).

31. R. E. Ciez, J. F. Whitacre, Examining different recycling processes for lithium-ion batteries. *Nat. Sustain.* **2**, 148–156 (2019).

32. S. Bobba, A. Podias, F. Di Persio, M. Messagie, P. Tecchio, M. A. Cusenza, U. Eynard, F. Mathieux, A. Pfrang, *Sustainability Assessment of Second Life Application of Automotive Batteries (SASLAB): JRC Exploratory Research (2016–2017): Final technical report: August 2018* (Publications Office of the European Union, 2018).

33. M. J. Goedkoop, R. Heijungs, M. A. J. Huijbregts, A. De Schryver, J. Struijs, R. Van Zelm, *ReCiPe 2008: A Life Cycle Impact Assessment Method which Comprises Harmonised Category Indicators at the Midpoint and the Endpoint Level* (PRé Sustainability, 2008).

34. P. A. Nelson, S. Ahmed, K. G. Gallagher, D. W. Dees, *Modeling the Performance and Cost of Lithium-Ion Batteries for Electric-Drive Vehicles, Third Edition* (National Renewable Energy Laboratory, 2019).

35. International Energy Agency, *World Energy Outlook 2019* (International Energy Agency, 2019).

36. A. Clerjon, F. Perdu, Matching intermittency and electricity storage characteristics through time scale analysis: An energy return on investment comparison. *Energ. Environ. Sci.* **12**, 693–705 (2019).

37. D.-S. Kourkoumpas, G. Benekos, N. Nikolopoulos, S. Karellas, P. Grammelis, E. Kakaras, A review of key environmental and energy performance indicators for the case of renewable energy systems when integrated with storage solutions. *Appl. Energy* **231**, 380–398 (2018).

38. G. Limpens, H. Jeanmart, Electricity storage needs for the energy transition: An EROI based analysis illustrated by the case of Belgium. *Energy* **152**, 960–973 (2018).

39. S. U. Muzayanha, C. S. Yudha, A. Nur, H. Widiyandari, H. Haerudin, H. Nilasary, F. Fathoni, A. Purwanto, A fast metals recovery method for the synthesis of lithium nickel cobalt aluminum oxide material from cathode waste. *Metals* **9**, 615 (2019).

40. Y. Shi, G. Chen, Z. Chen, Effective regeneration of $LiCoO_2$ from spent lithium-ion batteries: A direct approach towards high-performance active particles. *Green Chem.* **20**, 851–862 (2018).

41. M. Grützke, X. Mönnighoff, F. Horsthemke, V. Kraft, M. Winter, S. Nowak, Extraction of lithium-ion battery electrolytes with liquid and supercritical carbon dioxide and additional solvents. *RSC Adv.* **5**, 43209–43217 (2015).

42. X. Song, T. Hu, C. Liang, H. L. Long, L. Zhou, W. Song, L. You, Z. S. Wu, J. W. Liu, Direct regeneration of cathode materials from spent lithium iron phosphate batteries using a solid phase sintering method. *RSC Adv.* **7**, 4783–4790 (2017).

43. F. Piccinno, R. Hischier, S. Seeger, C. Som, From laboratory to industrial scale: A scale-up framework for chemical processes in life cycle assessment studies. *J. Clean. Prod.* **135**, 1085–1097 (2016).

44. J.-P. Skeete, P. Wells, X. Dong, O. Heidrich, G. Harper, Beyond the EVent horizon: Battery waste, recycling, and sustainability in the United Kingdom electric vehicle transition. *Energy Res. Soc. Sci.* **69**, 101581 (2020).

45. J. Baars, T. Domenech, R. Bleischwitz, H. E. Melin, O. Heidrich, Circular economy strategies for electric vehicle batteries reduce reliance on raw materials. *Nat. Sustain.* **4**, 71–79 (2021).

46. T. S. Schmidt, M. Beuse, X. Zhang, B. Steffen, S. F. Schneider, A. Pena-Bello, C. Bauer, D. Parra, Additional emissions and cost from storing electricity in stationary battery systems. *Environ. Sci. Technol.* **53**, 3379–3390 (2019).

47. L. Gaines, Q. Dai, J. T. Vaughey, S. Gillard, Direct recycling R&D at the ReCell center. *Dent. Rec.* **6**, 31 (2021).

48. D. L. Thompson, J. M. Hartley, S. M. Lambert, M. Shiref, G. D. J. Harper, E. Kendrick, P. Anderson, K. S. Ryder, L. Gaines, A. P. Abbott, The importance of design in lithium ion battery recycling – a critical review. *Green Chem.* **22**, 7585–7603 (2020).

49. D. Thompson, C. Hyde, J. M. Hartley, A. P. Abbott, P. A. Anderson, G. D. J. Harper, To shred or not to shred: A comparative techno-economic assessment of lithium ion battery hydrometallurgical recycling retaining value and improving circularity in LIB supply chains. *Resour. Conserv. Recycl.* **175**, 105741 (2021).

50. Environmental Protection Agency, *Emissions & Generation Resource Integrated Database (eGRID)* (United States Environmental Protection Agency, 2021).

51. International Energy Agency, *World Energy Statistics and Balances: Balances* (International Energy Agency, 2021).

52. Q. Dai, J. C. Kelly, L. Gaines, M. Wang, Life cycle analysis of lithium-ion batteries for automotive applications. *Batteries* **5**, 48 (2019).

53. H. E. Melin, M. A. Rajaeifar, A. Y. Ku, A. Kendall, G. Harper, O. Heidrich, Global implications of the EU battery regulation. *Science* **373**, 384-387 (2021).

54. Energy Information Administration, *International Energy Outlook 2019 with projections to 2050* (U.S. Energy Information Administration, 2019).

55. Energy Information Administration, *Annual Energy Outlook 2020 with projections to 2050* (U.S. Energy Information Administration, 2020).

56. N. Lebedeva, F. Di Persio, L. Brett, *Lithium ion battery value chain and related opportunities for Europe* (Joint Research Centre, European Commission, 2016).

57. Department of Energy, *Energy Storage Grand Challenge: Energy Storage Market Report* (U.S. Department of Energy, 2020).

58. International Energy Agency, *Global EV Outlook 2021: Trends and Developments in Electric Vehicle Markets* (International Energy Agency, 2021).

59. W. Shen, W. Han, T. J. Wallington, S. L. Winkler, China electricity generation greenhouse gas emission intensity in 2030: Implications for electric vehicles. *Environ. Sci. Technol.* **53**, 6063–6072 (2019).

60. M. Li, D. Patiño-Echeverri, J. Zhang, Policies to promote energy efficiency and air emissions reductions in China's electric power generation sector during the 11th and 12th five-year plan periods: Achievements, remaining challenges, and opportunities. *Energy Policy* **125**, 429–444 (2019).

61. Energy Information Administration, *Form EIA-923* (U.S. Energy Information Administration, 2020).

62. S.-T. Myung, F. Maglia, K.-J. Park, C. S. Yoon, P. Lamp, S.-J. Kim, Y.-K. Sun, Nickel-rich layered cathode materials for automotive lithium-ion batteries: Achievements and perspectives. *ACS Energy Letters* **2**, 196–223 (2017).

63. H.-J. Noh, S. Youn, C. S. Yoon, Y.-K. Sun, Comparison of the structural and electrochemical properties of layered $Li[Ni_xCo_yMn_z]O_2$ ($x$ = 1/3, 0.5, 0.6, 0.7, 0.8 and 0.85) cathode material for lithium-ion batteries. *J. Power Sources* **233**, 121–130 (2013).

64. J. E. Harlow, X. Ma, J. Li, E. Logan, Y. Liu, N. Zhang, L. Ma, S. L. Glazier, M. M. E. Cormier, M. Genovese, S. Buteau, A. Cameron, J. E. Stark, J. R. Dahn, A wide range of testing results on an excellent lithium-ion cell chemistry to be used as benchmarks for new battery technologies. *J. Electrochem. Soc.* **166**, A3031–A3044 (2019).

65. J. Li, A. R. Cameron, H. Li, S. Glazier, D. Xiong, M. Chatzidakis, J. Allen, G. A. Botton, J. R. Dahn, Comparison of single crystal and polycrystalline $LiNi_{0.5}Mn_{0.3}Co_{0.2}O_2$ positive electrode materials for high voltage Li-ion cells. *J. Electrochem. Soc.* **164**, A1534–A1544 (2017).

66. X.-G. Yang, T. Liu, C.-Y. Wang, Thermally modulated lithium iron phosphate batteries for mass-market electric vehicles. *Nat. Energy* **6**, 176–185 (2021).

67. H. Wang, S. Wang, X. Feng, X. Zhang, K. Dai, J. Sheng, Z. Zhao, Z. Du, Z. Zhang, K. Shen, C. Xu, Q. Wang, X. Sun, Y. Li, J. Ling, J. Feng, H. Wang, M. Ouyang, An experimental study on the thermal characteristics of the cell-to-pack system. *Energy* **227**, 120338 (2021).

68. R. Schmuch, R. Wagner, G. Hörpel, T. Placke, M. Winter, Performance and cost of materials for lithium-based rechargeable automotive batteries. *Nat. Energy* **3**, 267–278 (2018).

69. P. Xu, Z. Yang, X. Yu, J. Holoubek, H. Gao, M. Li, G. Cai, I. Bloom, H. Liu, Y. Chen, K. An, K. Z. Pupek, P. Liu, Z. Chen, Design and optimization of the direct recycling of spent Li-ion battery cathode materials. *ACS Sustain. Chem. Eng.* **9**, 4543–4553 (2021).

70. D. Bresser, D. Buchholz, A. Moretti, A. Varzi, S. Passerini, Alternative binders for sustainable electrochemical energy storage – the transition to aqueous electrode processing and bio-derived polymers. *Energ. Environ. Sci.* **11**, 3096–3127 (2018).

71. F. Zou, A. Manthiram, A review of the design of advanced binders for high-performance batteries. *Advanced Energy Materials* **10**, 2002508 (2020).

72. R. Wang, L. Feng, W. Yang, Y. Zhang, Y. Zhang, W. Bai, B. Liu, W. Zhang, Y. Chuan, Z. Zheng, H. Guan, Effect of different binders on the electrochemical performance of metal oxide anode for lithium-ion batteries. *Nanoscale Res. Lett.* **12**, 575 (2017).

73. Z. Chen, G.-T. Kim, D. Chao, N. Loeffler, M. Copley, J. Lin, Z. Shen, S. Passerini, Toward greener lithium-ion batteries: Aqueous binder-based $LiNi_{0.4}Co_{0.2}Mn_{0.4}O_2$ cathode material with superior electrochemical performance. *J. Power Sources* **372**, 180–187 (2017).

74. C.-Y. Wu, J.-G. Duh, Ionic network for aqueous-polymer binders to enhance the electrochemical performance of Li-ion batteries. *Electrochim. Acta* **294**, 22–27 (2019).

75. S. Rothermel, M. Evertz, J. Kasnatscheew, X. Qi, M. Grützke, M. Winter, S. Nowak, Graphite recycling from spent lithium-ion batteries. *ChemSusChem* **9**, 3473–3484 (2016).

76. Y. Yang, S. Song, S. Lei, W. Sun, H. Hou, F. Jiang, X. Ji, W. Zhao, Y. Hu, A process for combination of recycling lithium and regenerating graphite from spent lithium-ion battery. *Waste Manag.* **85**, 529–537 (2019).

77. L. Gaines, Lithium-ion battery recycling processes: Research towards a sustainable course. *Sustain. Mater. Technol.* **17**, e00068 (2018).

78. J. B. Dunn, L. Gaines, J. C. Kelly, C. James, K. G. Gallagher, The significance of Li-ion batteries in electric vehicle life-cycle energy and emissions and recycling's role in its reduction. *Energ. Environ. Sci.* **8**, 158–168 (2015).

79. L. A.-W. Ellingsen, G. Majeau-Bettez, B. Singh, A. K. Srivastava, L. O. Valøen, A. H. Strømman, Life cycle assessment of a lithium-ion battery vehicle pack. *J. Ind. Ecol.* **18**, 113–124 (2014).

80. G. Wernet, C. Bauer, B. Steubing, J. Reinhard, E. Moreno-Ruiz, B. Weidema, The ecoinvent database version 3 (part I): Overview and methodology. *Int. J. Life Cycle Assess.* **21**, 1218–1230 (2016).

81. R. Frischknecht, LCI modelling approaches applied on recycling of materials in view of environmental sustainability, risk perception and eco-efficiency. *Int. J. Life Cycle Assess.* **15**, 666–671 (2010).

82. J. B. Dunn, L. Gaines, J. Sullivan, M. Q. Wang, Impact of recycling on cradle-to-gate energy consumption and greenhouse gas emissions of automotive lithium-ion batteries. *Environ. Sci. Technol.* **46**, 12704–12710 (2012).

83. T. R. Hawkins, B. Singh, G. Majeau-Bettez, A. H. Strømman, Comparative environmental life cycle assessment of conventional and electric vehicles. *J. Ind. Ecol.* **17**, 53–64 (2013).

84. B. Li, X. Gao, J. Li, C. Yuan, Life cycle environmental impact of high-capacity lithium ion battery with silicon nanowires anode for electric vehicles. *Environ. Sci. Technol.* **48**, 3047–3055 (2014).

85. L. Ahmadi, A. Yip, M. Fowler, S. B. Young, R. A. Fraser, Environmental feasibility of re-use of electric vehicle batteries. *Sustain. Energy Technol. Assess.* **6**, 64–74 (2014).

86. Energy Information Administration, *How much electricity does an American home use?* (U.S. Energy Information Administration, 2020).

87. J. Neubauer, K. Smith, E. Wood, A. Pesaran, *Identifying and Overcoming Critical Barriers to Widespread Second Use of PEV Batteries* (National Renewable Energy Laboratory, 2015).

88. P. T. Benavides, Q. Dai, J. C. Kelly, J. B. Dunn, *Addition of Nickel Cobalt Aluminum (NCA) Cathode Material to GREET2* (Argonne National Laboratory, 2016).

89. Q. Dai, J. C. Kelly, J. B. Dunn, P. T. Benavides, *Update of Bill-of-materials and Cathode Materials Production for Lithium-ion Batteries in the GREET® Model* (Argonne National Laboratory, 2018).

90. M. Mistry, J. Gediga, S. Boonzaier, Life cycle assessment of nickel products. *Int. J. Life Cycle Assess.* **21**, 1559–1572 (2016).

91. Q. Dai, J. C. Kelly, A. Elgowainy, *Cobalt Life Cycle Analysis Update for the GREET® Model* (Argonne National Laboratory, 2018).

92. R. Hischier, *Treatment of Scrap Printed Wiring Boards, Shredding and Separation, RoW, Allocation at the Point of Substitution* (Ecoinvent database version 3.6).

93. J. B. Dunn, L. Gaines, M. Barnes, M. Wang, J. Sullivan, *Material and Energy Flows in the Materials Production, Assembly, and End-of-Life Stages of the Automotive Lithium-Ion Battery Life Cycle* (Office of Scientific and Technical Information, 2012).

94. G. Geisler, T. B. Hofstetter, K. Hungerbühler, Production of fine and speciality chemicals: Procedure for the estimation of LCIs. *Int. J. Life Cycle Assess.* **9**, 101–113 (2004).

95. D. L. Wood, J. D. Quass, J. Li, S. Ahmed, D. Ventola, C. Daniel, Technical and economic analysis of solvent-based lithium-ion electrode drying with water and NMP. *Drying Technol.* **36**, 234–244 (2018).

96. Department of Energy, National Energy Technology Laboratory, *Final Environmental Assessment for Compact Power, Inc. Electric Drive Vehicle Battery and Component Manufacturing Initiative Application* (U.S. Department of Energy, National Energy Technology Laboratory, 2010).

97. L.-P. He, S.-Y. Sun, X.-F. Song, J.-G. Yu, Leaching process for recovering valuable metals from the $LiNi_{1/3}Co_{1/3}Mn_{1/3}O_2$ cathode of lithium-ion batteries. *Waste Manag.* **64**, 171–181 (2017).

98. D. Bian, Y. Sun, S. Li, Y. Tian, Z. Yang, X. Fan, W. Zhang, A novel process to recycle spent $LiFePO_4$ for synthesizing $LiFePO_4$/C hierarchical microflowers. *Electrochim. Acta* **190**, 134–140 (2016).

99. Q. Dai, J. B. Dunn, J. C. Kelly, A. Elgowainy, *Update of Life Cycle Analysis of Lithium-ion Batteries in the GREET Model* (Argonne National Laboratory, 2017).

100. R. Zheng, L. Zhao, W. Wang, Y. Liu, Q. Ma, D. Mu, R. Li, C. Dai, Optimized Li and Fe recovery from spent lithium-ion batteries via a solution-precipitation method. *RSC Adv.* **6**, 43613–43625 (2016).

101. Y. H. Xie, H. J. Yu, Y. N. Ou, C. D. Li, Environmental impact assessment of recycling waste traction battery. *Inorganic Chemicals Industry* **47**, 43–46 (2015).

102. J. Bowyer, S. Bratkovich, K. Fernholz, M. Frank, H. Groot, J. Howe, E. Pepke, *Understanding Steel Recovery and Recycling Rates and Limitations to Recycling* (DOVETAIL PARTNERS INC., 2015).

103. International Aluminium Institute, *Global Aluminium Recycling: A Cornerstone of Sustainable Development* (International Aluminium Institute, 2009).

104. International Copper Association: Copper Alliance, *Copper Recycling* (International Copper Association: Copper Alliance, 2017).

105. D. Lee, B. Koo, C. B. Shin, S.-Y. Lee, J. Song, I.-C. Jang, J.-J. Woo, Modeling the effect of the loss of cyclable lithium on the performance degradation of a lithium-ion battery. *Energies* **12**, 4386 (2019).

106. D. Pritzl, T. Teufl, A. T. S. Freiberg, B. Strehle, J. Sicklinger, H. Sommer, P. Hartmann, H. A. Gasteiger, Editors' choice—Washing of nickel-rich cathode materials for lithium-ion batteries: Towards a mechanistic understanding. *J. Electrochem. Soc.* **166**, A4056–A4066 (2019).

107. U. Lee, S. Yang, Y. S. Jeong, Y. Lim, C. S. Lee, C. Han, Carbon dioxide liquefaction process for ship transportation. *Ind. Eng. Chem. Res.* **51**, 15122–15131 (2012).

108. C. Daniel, S. Sven, Battery recycling (Umicore, 2007).

109. C. L. Campion, W. Li, B. L. Lucht, Thermal decomposition of $LiPF_6$-based electrolytes for lithium-ion batteries. *J. Electrochem. Soc.* **152**, A2327 (2005).

110. D. Gielen, $CO_2$ removal in the iron and steel industry. *Energ. Conver. Manage.* **44**, 1027–1037 (2003).

111. Y.-F. Shen, W.-Y. Xue, W.-Y. Niu, Recovery of Co(II) and Ni(II) from hydrochloric acid solution of alloy scrap. *Trans. Nonferrous Met. Soc.* **18**, 1262–1268 (2008).

112. X. Zhou, W. He, G. Li, X. Zhang, S. Zhu, J. Huang, S. Zhu, in *2010 4th International Conference on Bioinformatics and Biomedical Engineering* (2010), pp. 1–4.

113. Department of Energy, Energy Information Administration, *Form EIA-923* (U.S. Department of Energy, Energy Information Administration, 2018).

114. R. E. Ciez, J. F. Whitacre, Comparison between cylindrical and prismatic lithium-ion cell costs using a process based cost model. *J. Power Sources* **340**, 273–281 (2017).
